# *N*-Acylated and *N*-Alkylated 2-Aminobenzothiazoles Are Novel Agents That Suppress the Generation of Prostaglandin E_2_

**DOI:** 10.3390/biom12020267

**Published:** 2022-02-07

**Authors:** Maria A. Theodoropoulou, Anastasia Psarra, Martin Erhardt, Aikaterini Nikolaou, Anna-Dimitra D. Gerogiannopoulou, Dimitra Hadjipavlou-Litina, Daiki Hayashi, Edward A. Dennis, Andrea Huwiler, George Kokotos

**Affiliations:** 1 Department of Chemistry, National and Kapodistrian University of Athens, Panepistimiopolis, 15771 Athens, Greece; martheod@chem.uoa.gr (M.A.T.); apsarra@chem.uoa.gr (A.P.); anikolao@chem.uoa.gr (A.N.); dgerogiann@chem.uoa.gr (A.-D.D.G.); 2 Center of Excellence for Drug Design and Discovery, National and Kapodistrian University of Athens, Panepistimiopolis, 15771 Athens, Greece; 3 Institute of Pharmacology, University of Bern, CH-3010 Bern, Switzerland; martinerhardt@gmx.net (M.E.); huwiler@pki.unibe.ch (A.H.); 4 Department of Pharmaceutical Chemistry, School of Pharmacy, Aristotelian University of Thessaloniki, 54124 Thessaloniki, Greece; hadjipav@pharm.auth.gr; 5 Department of Chemistry and Biochemistry and Department of Pharmacology, School of Medicine, University of California, San Diego, CA 92093-0601, USA; dhayashi@port.kobe-u.ac.jp (D.H.); edennis@ucsd.edu (E.A.D.)

**Keywords:** *N*-acylated 2-aminobenzothiazoles, *N*-alkylated 2-aminobenzothiazoles, anti-inflammatory agents, mesangial cells, prostaglandin E_2_

## Abstract

The quest for novel agents to regulate the generation of prostaglandin E_2_ (PGE_2_) is of high importance because this eicosanoid is a key player in inflammatory diseases. We synthesized a series of *N*-acylated and *N*-alkylated 2-aminobenzothiazoles and related heterocycles (benzoxazoles and benzimidazoles) and evaluated their ability to suppress the cytokine-stimulated generation of PGE_2_ in rat mesangial cells. 2-Aminobenzothiazoles, either acylated by the 3-(naphthalen-2-yl)propanoyl moiety (GK510) or *N*-alkylated by a chain carrying a naphthalene (GK543) or a phenyl moiety (GK562) at a distance of three carbon atoms, stand out in inhibiting PGE_2_ generation, with EC_50_ values ranging from 118 nM to 177 nM. Both GK510 and GK543 exhibit in vivo anti-inflammatory activity greater than that of indomethacin. Thus, *N*-acylated or *N*-alkylated 2-aminobenzothiazoles are novel leads for the regulation of PGE_2_ formation.

## 1. Introduction

The release of arachidonic acid (AA) from membrane glycerophospholipids, catalyzed by the action of phospholipases A_2_ (PLA_2_s), initiates the formation of a variety of bioactive lipids, which act as potent signaling mediators and are collectively referred to as eicosanoids [1,2]. Prostaglandins constitute a major and important class of such lipid signaling molecules [3]. In particular, prostaglandin E_2_ (PGE_2_) has attracted great interest because of its participation in both physiological and pathological processes. PGE_2_ plays a key role in inflammatory diseases [4]; however, it is also involved in tumorigenesis and cancer [5,6,7] as well as in the pathogenesis of Alzheimer’s disease (AD) [8]. As a consequence, the quest for agents able to inhibit and regulate the formation of PGE_2_ has been a highly active field during recent decades [9].

In addition to PLA_2_, which is the rate-limiting enzyme for the release of free arachidonic acid [10], a number of enzymes are involved in the biosynthesis of PGE_2_, and all of these enzymes have been targeted in the effort to produce anti-inflammatory agents. Cyclooxygenase-1 (COX-1) and cyclooxygenase-2 (COX-2) catalyze the oxidation of AA to prostaglandin H_2_ (PGH_2_) [1]. Then, prostaglandin synthases, such as microsomal prostaglandin E synthase-1 (mPGES-1), catalyze the formation of PGE_2_ [11]. Once this lipid is formed, it may interact with four distinct receptors (EP1 to EP4) to exert its action [12]. The widely used non-steroidal anti-inflammatory drugs (NSAIDs) are non-selective COX inhibitors, which usually exhibit gastrointestinal side effects, while selective COX-2 inhibitors, such as celecoxib, overcome the side effects of NSAIDs; however, they present potential cardiovascular toxicity [13]. PLA_2_ inhibitors have been developed and studied for decades [10,14]; however, none of them have reached the market. Currently, there is a great interest in mPGES-1 inhibitors [11,15,16] because they are considered a safer alternative to COX-2 inhibitors, as they lack cardiovascular toxicity, although further research is required to prove their clinical efficiency and safety.

**Figure 1:**
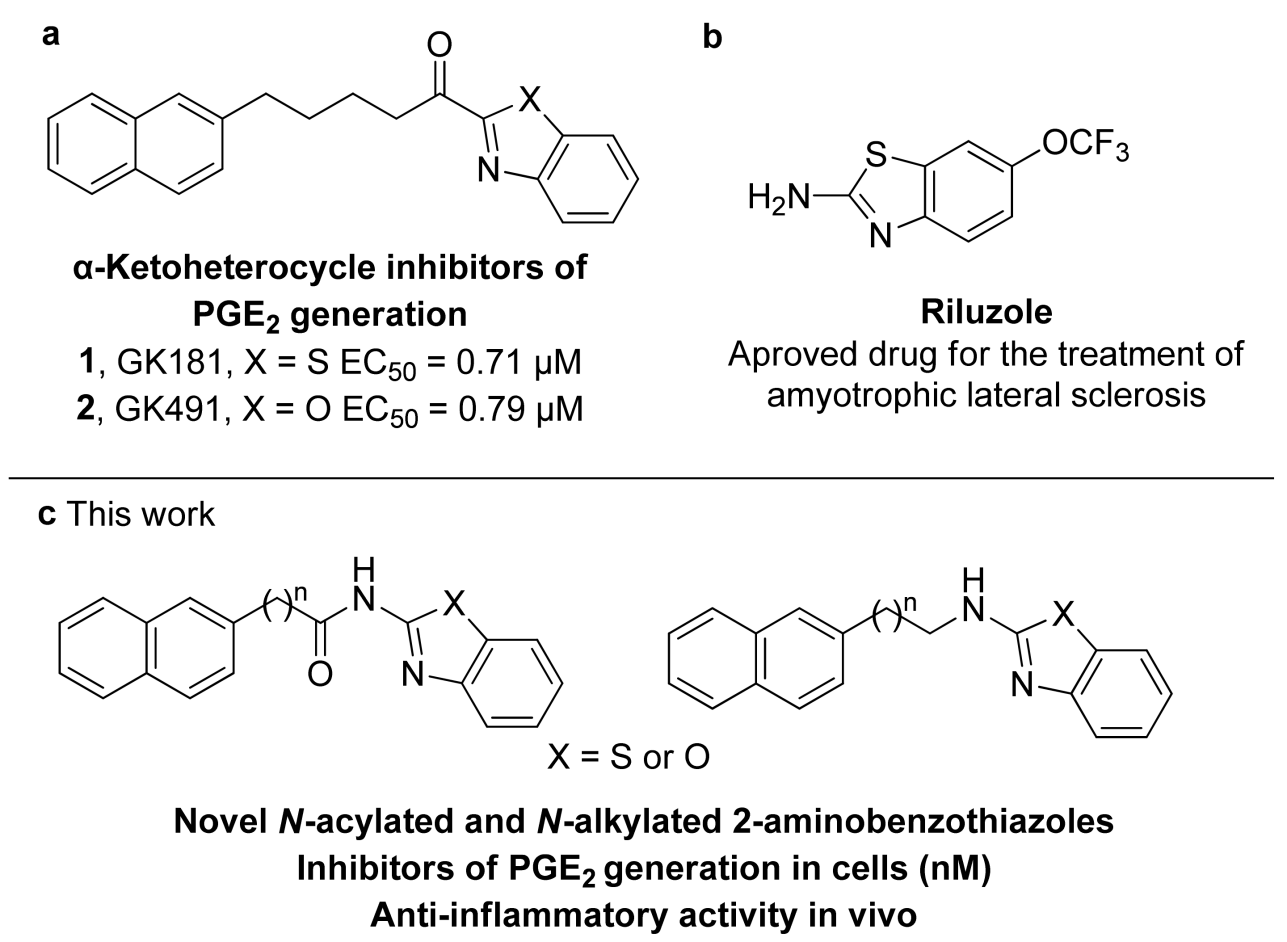
Bioactive 2-substituted benzothiazoles and benzoxazoles. (**a**) Previously developed α-ketoheterocycle inhibitors of PGE_2_ generation; (**b**) riluzole; (**c**) *N*-acylated and *N*-alkylated 2-aminobenzothiazole and benzoxazole inhibitors of PGE_2_ generation designed in the present study.

As part of our effort to develop PLA_2_ inhibitors as novel anti-inflammatory agents [17,18,19], we have shown that inhibitors of secreted PLA_2_ are able to suppress the production of PGE_2_ in mesangial cells [20]. Inspired by thiazolyl ketones exhibiting an interesting ability to inhibit PGE_2_ formation and in vivo anti-inflammatory properties [19], we have most recently presented a series of α-ketoheterocycles and we have demonstrated that the α-ketobenzothiazole derivative GK181 (**1**, Figure 1a) and α-ketobenzoxazole derivative GK491 (**2**, Figure 1a) inhibit PGE_2_ formation in rat mesangial cells with half maximal effective concentrations (EC_50_) values of 0.71 μM and 0.79 μM, respectively [21]. Benzothiazole is indeed an important heterocyclic skeleton that plays an important role in medicinal chemistry as a key template for the development of various therapeutic agents [22,23,24,25]. Among the various derivatives based on the privileged benzothiazole molecular scaffold, 2-aminobenzothiazoles exhibit diverse biological properties; for example, riluzole is a marketed drug (Figure 1b) for treating amyotrophic lateral sclerosis [26]. In 2012, a series of 2-aminothiazoles were reported and their evaluation concluded that disubstituted 2-*N*-arylaminothiazoles may inhibit PGE_2_ production in cells [27]. Recently, Chini et al., employing a combinatorial virtual screening, have identified substituted 2-benzoylaminobenzothiazoles able to suppress PGE_2_ levels [28].

The promising properties of α-ketobenzothiazole **1** and α-ketobenzoxazole **2** in inhibiting PGE_2_ generation at a cellular level, observed by our group [21], prompted us to further explore compounds based on the privileged benzothiazole scaffold. We present, herein, routes for the synthesis of a variety of *N*-acylated and *N*-alkylated 2-aminobenzothiazoles and the corresponding benzoxazoles and benzimidazoles. A structure-activity relationship study for the ability of such compounds to inhibit the cytokine-stimulated generation of PGE_2_ in rat mesangial cells resulted in the discovery of novel naphthalene-containing *N*-acylated or *N*-alkylated 2-aminobenzothiazoles (Figure 1c), which inhibited PGE_2_ generation at a nanomolar level and presented anti-inflammatory in vivo activity in a rat-paw carrageenan-edema assay.

## 2. Materials and Methods

### 2.1. General Chemistry Methods

The chromatographic purification of products was accomplished using forced-flow chromatography on a Merck^®^ (Merck, Darmstadt, Germany) Kieselgel 60 F_254_ 230–400 mesh. Thin-layer chromatography (TLC) was performed on aluminum-backed silica plates (0.2 mm, 60 F_254_). The visualization of the developed chromatograms was performed by fluorescence quenching using phosphomolybdic acid, ninhydrin or potassium permanganate stains. The melting points were determined on a Buchi^®^ 530 apparatus (Buchi, Flawil, Switzerland) and were uncorrected. ^1^H and ^13^C NMR spectra were recorded on a Varian^®^ Mercury (Varian, Palo Alto, CA, USA) (200 MHz and 50 MHz, respectively) or a Bruker Avance Neo (Bruker, Faellanden, Switzerland) (400 MHz and 100 MHz, respectively) and were internally referenced to residual solvent signals. The data for ^1^H NMR are reported as follows: chemical shift (δ ppm), multiplicity (s = singlet, d = doublet, t = triplet, quint = quintet, m = multiplet, and br = broad signal), coupling constant, integration and peak assignment. The data for ^13^C NMR are reported in terms of the chemical shift (δ ppm). High-resolution mass spectrometry (HRMS) spectra were recorded on a Bruker^®^ Maxis Impact QTOF (Bruker Daltonics, Bremen, Germany) spectrometer. The purity of all the compounds subjected to biological tests was determined using analytical HPLC and was found to be ≥95%. The HPLC analyses were carried out on a Shimadzu LC-2010AHT system and a Phenomenex, Luna C18(2) 100A (150 × 2 mm, 5 μm) analytical column, using H_2_O/acetonitrile 65/35 *v*/*v*, with a gradient to 40:60 *v*/*v*, at a flow rate of 1.0 mL/min.

### 2.2. General Procedure for the Synthesis of Amides ***5a–f**, **7**, **9a,b**, **14***

To a stirred solution of the carboxylic acid **4a,b**, **6**, **8a,b**, or **13** (1.0 mmol) in dry CH_2_Cl_2_ (10 mL) and Et_3_N (0.1 mL), cooled to 0 °C, 1-hydroxybenzotriazole (HOBt, 135 mg, 1.0 mmol), amine **3a–c** or **12** (1.2 mmol) and 1-ethyl-3-(3-dimethylaminopropyl)carbodiimide hydrochloride (EDCI∙HCl, 230 mg, 1.2 mmol) were added consecutively. The reaction mixture was left stirring for 1 h at 0 °C and for 16 h at room temperature. The solvent was then evaporated under reduced pressure; the residue was diluted in ethyl acetate and washed with water (10 mL), an aqueous solution of 1N HCl (10 mL), water (10 mL), an aqueous solution of 5% NaHCO_3_ (10 mL) and brine (10 mL), consecutively. The organic layer was dried over Na_2_SO_4_ and concentrated under reduced pressure. The resulting amide was purified by flash chromatography, eluting with the appropriate mixture of solvents.

#### 2.2.1. N-(Benzo[d]Thiazol-2-yl)-4-(Naphthalen-2-yl)Butanamide (**5a**)

Elution solvent system: EtOAc:petroleum ether (40–60 °C) 3:7; Yield 30% (104 mg); White solid; mp: 53–55 °C; ^1^H NMR (200 MHz, CDCl_3_): *δ* = 7.75–7.43 (m, 6H, 6 × ArH), 7.33–7.06 (m, 5H, 5 × ArH) 4.13 (br, 1H, NH), 2.73 (t, *J* = 7.5 Hz, 2H, PhCH_2_), 2.42 (t, *J* = 7.4 Hz, 2H, CH_2_CO), 2.02 (quint, *J* = 7.2 Hz, 2H, CH_2_); ^13^C NMR (50 MHz, CDCl_3_ and CD_3_OD): *δ* = 172.1, 152.9, 147.5, 138.3, 133.3, 131.8, 131.4, 127.7, 127.2, 127.1, 126.8, 126.3, 126.0, 125.6, 124.9, 123.6, 121.1, 120.0, 34.8, 25.9; HRMS (ESI) [M − H]^−^ *m*/*z*: 345.1058 (calculated for [C_21_H_17_N_2_OS]^−^ 345.1067).

#### 2.2.2. N-(Benzo[d]Thiazol-2-yl)-3-(Naphthalen-2-yl)Propanamide (**5b**, GK510)

Elution solvent system: EtOAc:petroleum ether (40–60 °C) 3:7; Yield 35% (116 mg); White solid; mp: 46–48 °C; ^1^H NMR (200 MHz, CDCl_3_): *δ* = 7.79–7.47 (m, 6H, 6 × ArH), 7.39–7.15 (m, 5H, 5 × ArH), 3.79 (br, 1H, NH), 3.14 (t, *J* = 7.6 Hz, 2H, PhCH_2_), 2.81 (t, *J* = 7.7 Hz, 2H, CH_2_CO); ^13^C NMR (50 MHz, CDCl_3_ and CD_3_OD): *δ* = 171.4, 165.7, 147.5, 137.4, 133.3, 131.9, 131.5, 128.0, 127.4, 127.3, 126.6, 126.3, 126.1, 125.9, 125.3, 123.8, 121.3, 120.1, 37.4, 30.8; HRMS (ESI) [M − H]^−^ *m*/*z*: 331.0900 (calculated for [C_20_H_15_N_2_OS]^−^ 331.0911).

#### 2.2.3. N-(Benzo[d]Oxazol-2-yl)-3-(Naphthalen-2-yl)Propanamide (**5c**)

Elution solvent system: EtOAc:petroleum ether (40–60 °C) 5:5; Yield 50% (158 mg); White solid; mp: 49–51 °C; ^1^H NMR (200 MHz, DMSO-d_6_): *δ* = 7.90–7.20 (m, 11H, 11 × ArH), 3.13–3.06 (m, 2H, PhCH_2_), 2.98–2.90 (m, 2H, CH_2_CO); ^13^C NMR (50 MHz, DMSO-d_6_): *δ* = 170.4, 155.2, 147.6, 140.7, 138.5, 133.1, 131.7, 127.9, 127.5, 127.4, 127.3, 126.3, 126.1, 125.4, 124.5, 123.5, 118.1, 110.0, 37.5, 30.3; HRMS (ESI) [M + Na]^+^ *m*/*z*: 339.1106 (calculated for [C_20_H_16_N_2_NaO_2_]^+^ 339.1104).

#### 2.2.4. N-(Benzo[d]Oxazol-2-yl)-4-(Naphthalen-2-yl)Butanamide (**5d**)

Elution solvent system: EtOAc:petroleum ether (40–60 °C) 6:4; Yield 32% (106 mg); White solid; mp: 52–55 °C; ^1^H NMR (200 MHz, DMSO-d_6_): *δ* = 11.64 (br, 1H, NH), 7.89–7.22 (m, 11H, 11 × ArH), 2.81 (t, 2H, *J* = 7.6 Hz, PhCH_2_), 2.59 (t, *J* = 7.3 Hz, 2H, CH_2_CO), 2.01 (quint, *J* = 7.5 Hz, 2H, CH_2_); ^13^C NMR (50 MHz, DMSO-d_6_): *δ* = 170.3, 154.8, 147.2, 140.3, 138.8, 132.8, 131.2, 127.4, 127.1, 126.9, 125.8, 125.6, 124.8, 124.1, 123.0, 117.7, 109.6, 34.9, 34.1, 25.5; HRMS (ESI) [M − H]^−^ *m*/*z*: 329.1298 (calculated for [C_21_H_17_N_2_O_2_]^−^ 329.1296).

#### 2.2.5. N-(1H-Benzo[d]Imidazol-2-yl)-3-(Naphthalen-2-yl)Propanamide (**5e**)

Elution solvent system: EtOAc:petroleum ether (40–60 °C) 6:4; Yield 19% (60 mg); White solid; mp: 47–49 °C; ^1^H NMR (200 MHz, DMSO-d_6_): *δ* = 12.06 (br, 1H, NH), 11.58 (br, 1H, NH), 7.87–7.68 (m, 5H, 5 × ArH), 7.51–7.36 (m, 4H, 4 × ArH), 7.10–7.02 (m, 2H, 2 × ArH), 3.13 (t, *J* = 7.5 Hz, 2H, PhCH_2_), 2.93–2.82 (m, 2H, CH_2_CO); ^13^C NMR (50 MHz, DMSO-d_6_): *δ* = 171.7, 146.6, 138.5, 133.2, 131.7, 127.9, 127.6, 127.4, 127.3, 126.3, 126.1, 125.4, 37.0, 30.7; HRMS (ESI) [M + Na]^+^ *m*/*z*: 338.1263 (calculated for [C_20_H_17_N_3_NaO]^+^ 338.1264).

#### 2.2.6. N-(1H-Benzo[d]Imidazol-2-yl)-4-(Naphthalen-2-yl)Butanamide (**5f**)

Elution solvent system: Et_2_O; Yield 21% (69 mg); White solid; mp: 56–58 °C; ^1^H NMR (200 MHz, DMSO-d_6_): *δ* = 12.06 (br, 1H, NH), 11.51 (br, 1H, NH), 7.89–7.81 (m, 4H, 4 × ArH), 7.51–7.44 (m, 4H, 4 × ArH), 7.11–7.04 (m, 3H, 3 × ArH), 2.85–2.68 (m, 2H, PhCH_2_), 2.60–2.52 (m, 2H, CH_2_O), 2.13–1.93 (m, 2H, CH_2_); ^13^C NMR (50 MHz, DMSO-d_6_): *δ* = 171.8, 146.1, 138.7, 132.7, 131.2, 131.1, 127.3, 127.3, 127.0, 126.89, 126.86, 126.8, 125.74, 125.68, 125.6, 125.5, 124.8, 124.7, 120.5, 34.4, 25.8, 19.1; HRMS (ESI) [M − H]^−^ *m*/*z*: 328.1443 (calculated for [C_21_H_18_N_3_O]^−^ 328.1455).

#### 2.2.7. N-(Benzo[d]Thiazol-2-yl)-2-(Naphthalen-2-yloxy)Acetamide (**7**)

Elution solvent system: Et_2_O:petroleum ether (40–60 °C) 3:7 to 4:6; Yield 44% (147 mg); White solid; mp: 153–155 °C; ^1^H NMR (200 MHz, CDCl_3_ and CD_3_OD): *δ* = 7.93–7.68 (m, 5H, 5 × ArH), 7.52–7.14 (m, 6H, 6 × ArH), 4.86 (s, 2H, CH_2_); ^13^C NMR (50 MHz, CDCl_3_ and CD_3_OD): *δ* = 167.4, 157.5, 154.8, 147.6, 134.2, 131.8, 130.1, 129.7, 127.7, 127.0, 126.8, 126.6, 124.54, 124.48, 121.5, 120.9, 118.1, 107.5, 66.8; HRMS (ESI) [M − H]^−^ *m*/*z*: 333.0697 (calculated for [C_19_H_13_N_2_O_2_S]^−^ 333.0703).

#### 2.2.8. (E)-N-(Benzo[d]Thiazol-2-yl)-3-(Naphthalen-2-yl)Acrylamide (**9a**)

Elution solvent system: EtOAc:petroleum ether (40–60 °C) 3:7; Yield 17% (56 mg); Pale white solid; mp: 228–230 °C; ^1^H NMR (200 MHz, CDCl_3_ and CD_3_OD): *δ* = 8.02–7.61 (m, 7H, 7 × ArH), 7.51–7.23 (m, 5H, 4 × ArH, CHPh), 6.81 (d, *J* = 15.6 Hz, 1H, CHCO); ^13^C NMR (50 MHz, CDCl_3_ and CD_3_OD): *δ* = 164.9, 159.7, 148.1, 145.2, 134.6, 133.5, 132.1, 131.9, 130.8, 129.0, 128.8, 128.0, 127.7, 127.0, 126.6, 124.2, 123.5, 121.7, 120.5, 118.5; HRMS (ESI) [M − H]^−^ *m*/*z*: 329.0734 (calculated for [C_20_H_13_N_2_OS]^−^ 329.0754).

#### 2.2.9. (E)-N-(Benzo[d]Thiazol-2-yl)-3-(4-Methoxyphenyl)Acrylamide (**9b**)

Elution solvent system: EtOAc:petroleum ether (40–60 °C) 3:7; Yield 16% (50 mg); White solid; mp: 252–254 °C; lit. [29] 243–245 °C; ^1^H NMR (200 MHz, CDCl_3_ and CD_3_OD): *δ* = 7.85–7.66 (m, 3H, 3 × ArH), 7.49–7.25 (m, 4H, 3 × ArH, CHPh), 6.87 (d, *J* = 8.5 Hz, 2H, 2 × ArH), 6.56 (d, *J* = 15.7 Hz, 1H, CHCO), 3.81 (s, 3H, CH_3_); ^13^C NMR (50 MHz, CDCl_3_ and CD_3_OD): *δ* = 164.9, 161.5, 147.8, 144.5, 132.3, 129.9, 126.8, 126.1, 123.7, 121.2, 120.1, 115.5, 114.2, 113.5, 55.2; HRMS (ESI) [M − H]^−^ *m*/*z*: 309.0690 (calculated for [C_17_H_13_N_2_O_2_S]^−^ 309.0703).

#### 2.2.10. N-(3-(Naphthalen-2-yl)Propyl)Benzo[d]Thiazole-2-Carboxamide (**14**)

Elution solvent system: EtOAc:petroleum ether (40–60 °C) 15:85; Yield 21% (73 mg); White solid; mp: 48–50 °C; ^1^H NMR (200 MHz, CDCl_3_): *δ* = 8.06–7.23 (m, 12H, 12 × ArH, NH), 3.58 (q, *J* = 7.0 Hz, 2H, CH_2_N), 2.92 (t, *J* = 7.9 Hz, 2H, PhCH_2_), 2.11 (quint, *J* = 7.2 Hz, 2H, CH_2_); ^13^C NMR (50 MHz, CDCl_3_): *δ* = 163.9, 159.9, 152.8, 138.6, 137.1, 133.6, 128.1, 127.6, 127.4, 127.0, 126.7, 126.6, 126.5, 126.0, 125.2, 124.2, 122.4, 39.5, 33.4, 30.9; HRMS (ESI) [M + H]^+^ *m*/*z*: 347.1216 (calculated for [C_21_H_19_N_2_OS]^+^ 347.1213); HRMS (ESI) [M + Na]^+^ *m*/*z*: 369.1037 (calculated for [C_21_H_18_N_2_NaOS]^+^ 369.1032).

### 2.3. N-(Benzo[d]Thiazol-2-yl)-3-(4-Methoxyphenyl)Propanamide *(**11**)*

Carboxylic acid **10** (180 mg, 1.0 mmol) was stirred with SOCl_2_ (0.7 mL, 10 mmol) under reflux for 4 h. After the concentration of the mixture, the residue was dissolved in CH_2_Cl_2_ and the solvent was removed under reduced pressure (three times). Then, the residue was dissolved in dry CH_2_Cl_2_ (5 mL), and **3a** (75 mg, 0.5 mmol) and Et_3_N (0.08 mL, 0.5 mmol) were added. The reaction mixture was left stirring, under argon, for 16 h at room temperature. Upon the completion of the reaction, the organic layer was washed with water and dried over Na_2_SO_4_ and the solvent was evaporated under reduced pressure. Purification by flash chromatography eluting with a mixture of EtOAc:petroleum ether (40–60 °C) 3:7 afforded the desired product. Yield 59% (184 mg); White solid; mp: 172–174 °C; ^1^H NMR (200 MHz, CDCl_3_): *δ* = 11.76 (br, 1H, NH), 7.97–7.76 (m, 1H, ArH), 7.69–7.59 (m, 1H, ArH), 7.45–7.29 (m, 2H, 2 × ArH), 6.90 (d, *J* = 8.8 Hz, 2H, 2 × ArH), 6.71 (d, *J* = 8.8 Hz, 2H, 2 × ArH), 3.74 (s, 3H, CH_3_), 2.94 (t, *J* = 7.1 Hz, 2H, PhCH_2_), 2.71 (t, *J* = 7.3 Hz, 2H, CH_2_CO); ^13^C NMR (50 MHz, CDCl_3_): *δ* = 171.3, 159.9, 158.0, 147.5, 131.7, 129.1, 126.4, 124.0, 121.6, 120.3, 113.8, 55.2, 38.3, 30.1; HRMS (ESI) [M − H]^−^ *m*/*z*: 311.0880 (calculated for [C_17_H_15_N_2_O_2_S]^−^ 311.0860).

### 2.4. General Procedure for the Synthesis of Carbamates ***16a,b***

To a stirred solution of alcohol **15a,b** (1.7 mmol) in dry CH_2_Cl_2_ (17 mL), cooled to 0 °C and under argon, triphosgene (297 mg, 1.0 mmol) and Et_3_N (0.25 mL, 1.7 mmol) were added and the mixture was stirred for 15 min at 0 °C and for 15 min at room temperature. Then, a cold saturated aqueous solution of NaHCO_3_ (20 mL) was added dropwise, the mixture was extracted with CH_2_Cl_2_ (20 mL), and the organic layer was washed with brine (15 mL). The organic layer was dried over Na_2_SO_4_, and the solvent was evaporated under reduced pressure. The residue was dissolved in dry tetrahydrofuran (THF, 2.8 mL) and Et_3_N (0.55 mL) and placed in a pressure vessel. **3a** (255 mg, 1.7 mmol) and a catalytic amount (12 mg, 0.1 mmol) of 4-(dimethylamino)pyridine (4-DMAP) were added, and the reaction mixture was left stirring for 48 h. Then, the mixture was concentrated under reduced pressure, and purification by flash chromatography, eluting with the appropriate mixture of solvents, provided the desired product.

#### 2.4.1. 4-Methoxybenzyl Benzo[d]Thiazol-2-ylcarbamate (**16a**)

Elution solvent system: EtOAc:petroleum ether (40–60 °C) 3:7; Yield 18% (57 mg); White solid; mp: 141–143 °C; ^1^H NMR (200 MHz, CDCl_3_): *δ* = 7.56 (d, *J* = 7.9 Hz, 1H, ArH), 7.46 (d, *J* = 8.1 Hz, 1H, ArH), 7.34–7.22 (m, 3H, 3 × ArH), 7.11–7.02 (m, 1H, ArH), 6.87 (d, *J* = 8.8 Hz, 2H, 2 × ArH), 6.33 (br, 1H, NH), 4.53 (s, 2H, CH_2_), 3.79 (s, 3H, CH_3_); ^13^C NMR (50 MHz, CDCl_3_): *δ* = 167.7, 159.3, 152.1, 139.9, 129.3, 129.0, 128.6, 125.9, 121.6, 120.8, 118.8, 114.1, 55.3, 48.9; HRMS (ESI) [M + Na]^+^ *m*/*z*: 337.0614 (calculated for [C_16_H_14_N_2_NaO_3_S]^+^ 337.0617).

#### 2.4.2. Naphthalen-2-ylmethyl Benzo[d]Thiazol-2-ylcarbamate (**16b**)

Elution solvent system: EtOAc:petroleum ether (40–60 °C) 1:9 to 3:7; Yield 13% (43 mg); White solid; mp: 209–211 °C; ^1^H NMR (200 MHz, CDCl_3_): *δ* = 7.93–7.77 (m, 4H, 4 × ArH), 7.73–7.62 (m, 2H, 2 × ArH), 7.55–7.42 (m, 3H, 3 × ArH), 7.14 (t, *J* = 7.6 Hz, 1H, ArH), 6.95 (t, *J* = 7.6 Hz, 1H, ArH), 5.47 (s, 2H, CH_2_); ^13^C NMR (50 MHz, CDCl_3_): *δ* = 161.1, 153.8, 148.3, 133.2, 133.1, 132.2, 131.3, 128.5, 128.1, 128.0, 127.6, 126.5, 126.4, 126.1, 125.9, 123.5, 121.0, 120.4, 68.5; HRMS (ESI) [M + Na]^+^ *m*/*z*: 357.0675 (calculated for [C_19_H_14_N_2_NaO_2_S]^+^ 357.0668); HRMS (ESI) [M − H]^−^ *m*/*z*: 333.0696 (calculated for [C_19_H_13_N_2_O_2_S]^−^ 333.0703).

### 2.5. General Procedure for the Synthesis of Hemiaminal Ethers ***18a–h***

To a stirred solution of alcohol **17a–d** (1.0 mmol) in dry CH_2_Cl_2_ (2 mL), (2,2,6,6-τetramethylpiperidin-1-yl)oxyl (TEMPO, 16 mg, 0.1 mmol) and iodobenzene diacetate (360 mg, 1.2 mmol) were added consecutively, and the reaction mixture was left stirring at room temperature for 1 h. The mixture was transferred to a separatory funnel and washed with an aqueous solution of 10% Na_2_S_2_O_3_ (5 mL), an aqueous solution of 5% NaHCO_3_ (5 mL), and brine (5 mL), consecutively. The organic layer was dried over Na_2_SO_4_, and the solvent was evaporated under reduced pressure. The crude aldehyde was used directly in the next step.

To a stirred solution of **3a** (150 mg, 1.0 mmol) in absolute MeOH (1 mL), Na_2_SO_4_ (284 mg, 2.0 mmol) and the crude aldehyde (1.0 mmol) were added, and the mixture was left stirring for 16 h. It was then cooled to 0 °C, and NaBH_4_ (61 mg, 1.6 mmol) was added in small portions. The mixture was left stirring for at least 4 h, being TLC-monitored. Upon completion, the reaction was quenched with H_2_O (2 mL), and MeOH was evaporated under reduced pressure. After filtration, the filtrate was extracted with ethyl acetate (3 × 5 mL). Purification by flash chromatography, eluting with an appropriate mixture of EtOAc:petroleum ether (40–60 °C), afforded the desired hemiaminal ether.

#### 2.5.1. N-(1-Methoxy-3-(Naphthalen-2-yl)Propyl)Benzo[d]Thiazol-2-Amine (**18a**)

Elution solvent system: EtOAc:petroleum ether (40–60 °C) 3:7; Yield 63% (219 mg); Colorless syrup; ^1^H NMR (200 MHz, CDCl_3_): *δ* = 8.31 (br, 1H, NH), 7.90–7.17 (m, 11H, 11 × ArH), 4.98 (t, *J* = 6.0 Hz, 1H, CH), 3.55 (s, 3H, OCH_3_), 3.03 (t, *J* = 7.6 Hz, 2H, PhCH_2_), 2.47–2.19 (m, 2H, CH_2_); ^13^C NMR (50 MHz, CDCl_3_): *δ* = 167.1, 151.3, 137.9, 133.2, 131.7, 129.8, 127.7, 127.2, 127.1, 126.7, 126.2, 125.8, 125.6, 124.9, 121.5, 120.5, 118.6, 87.3, 54.7, 36.4, 31.0; HRMS (ESI) [M − H]^−^ *m*/*z*: 347.1219 (calculated for [C_21_H_19_N_2_OS]^−^ 347.1224).

#### 2.5.2. N-(1-Methoxy-3-Phenylpropyl)Benzo[d]Thiazol-2-Amine (**18b**)

Elution solvent system: EtOAc:petroleum ether (40–60 °C) 2:8; Yield 97% (289 mg); Orange oil; ^1^H NMR (200 MHz, CDCl_3_): *δ* = 7.61 (dd, *J* = 8.3, 2.8 Hz, 2H, 2 × ArH), 7.37–7.08 (m, 8H, 7 × ArH, NH), 4.88 (t, *J* = 6.3 Hz, 1H, CH), 3.45 (s, 3H, OCH_3_), 2.79 (t, *J* = 7.8 Hz, 2H, PhCH_2_), 2.26–2.04 (m, 2H, C*H_2_*CH); ^13^C NMR (50 MHz, CDCl_3_): *δ* = 167.0, 151.4, 140.8, 130.0, 128.4, 128.3, 126.1, 126.0, 121.9, 120.8, 118.9, 87.3, 55.1, 36.9, 31.1; HRMS (ESI) [M + H]^+^ *m*/*z*: 299.1200 (calculated for [C_17_H_19_N_2_OS]^+^ 299.1213).

#### 2.5.3. N-(1-Methoxy-3-(4-Methoxyphenyl)Propyl)Benzo[d]Thiazol-2-Amine (**18c**)

Elution solvent system: EtOAc:petroleum ether (40–60 °C) 2:8; Yield 70% (230 mg); Yellowish oil; ^1^H NMR (200 MHz, CDCl_3_): *δ* = 8.06 (br, 1H, NH), 7.64 (d, *J* = 8.1 Hz, 2H, 2 × ArH), 7.40–7.31 (m, 1H, ArH), 7.20–7.06 (m, 3H, 3 × ArH), 6.82 (d, *J* = 8.6 Hz, 2H, 2 × ArH), 4.87 (t, *J* = 6.3 Hz, 1H, CH), 3.79 (s, 3H, PhOCH_3_), 3.47 (s, 3H, OCH_3_), 2.75 (t, *J* = 7.9 Hz, 2H, PhCH_2_), 2.28–2.06 (m, 2H, C*H_2_*CH); ^13^C NMR (50 MHz, CDCl_3_): *δ* = 167.2, 157.7, 151.4, 132.7, 129.9, 129.2, 126.0, 121.7, 120.7, 118.7, 113.7, 87.3, 55.0, 54.9, 36.9, 30.2; HRMS (ESI) [M + H]^+^ *m*/*z*: 329.1309 (calculated for [C_18_H_21_N_2_O_2_S]^+^ 329.1318).

#### 2.5.4. N-(1-Methoxy-3-(Naphthalen-1-yl)Propyl)Benzo[d]Thiazol-2-Amine (**18d**)

Elution solvent system: EtOAc:petroleum ether (40–60 °C) 25:75; Yield 55% (191 mg); Yellowish oil; ^1^H NMR (200 MHz, CDCl_3_): *δ* = 8.05–7.98 (m, 1H, ArH), 7.90–7.83 (m, 1H, ArH), 7.78–7.63 (m, 3H, 3 × ArH), 7.52–7.13 (m, 7H, 6 × ArH, ΝH), 5.02 (t, *J* = 6.1 Hz, 1H, CH), 3.52 (s, 3H, OCH_3_), 3.29 (t, *J* = 7.8 Hz, 2H, PhCH_2_), 2.48–2.19 (m, 2H, C*H_2_*CH); ^13^C NMR (50 MHz, CDCl_3_): *δ* = 167.1, 151.6, 136.9, 133.8, 131.6, 130.1, 128.7, 126.8, 126.04, 125.96, 125.8, 125.4, 123.5, 121.8, 120.7, 118.9, 87.7, 55.1, 36.2, 28.3; HRMS (ESI) [M + Na]^+^ *m*/*z*: 371.1189 (calculated for [C_21_H_20_N_2_NaOS]^+^ 371.1189).

#### 2.5.5. N-(1-Methoxy-3-(Naphthalen-2-yl)Propyl)-6-(Trifluoromethoxy)Benzo[d]Thiazol-2-Amine (**18g**)

Elution solvent system: EtOAc:petroleum ether (40–60 °C) 25:75; Yield 38% (164 mg); White solid; mp: 78–80 °C; ^1^H NMR (200 MHz, CDCl_3_): *δ* = 7.85–7.14 (m, 11H, 10 × ArH, NH), 4.91 (t, *J* = 6.1 Hz, 1H, CH), 3.46 (s, 3H, OCH_3_), 2.96 (t, *J* = 7.8 Hz, 2H, PhCH_2_), 2.41–2.13 (m, 2H, C*H_2_*CH); ^13^C NMR (50 MHz, CDCl_3_): *δ* = 167.4, 150.3, 143.7 (q, *J* = 2.3 Hz), 138.0, 133.4, 132.0, 130.8, 128.1, 127.5, 127.3, 126.9, 126.5, 125.9, 125.3, 120.6 (q, *J* = 254.9 Hz), 119.7, 119.2, 113.9, 87.2, 55.2, 36.7, 31.2; ^19^F NMR (188 MHz, CDCl_3_): *δ* = −16.2 (OCF_3_).

#### 2.5.6. N-(1-Methoxy-3-(Naphthalen-1-yl)Propyl)-6-(Trifluoromethoxy)Benzo[d]Thiazol-2-Amine (**18h**)

Elution solvent system: EtOAc:petroleum ether (40–60 °C) 25:75; Yield 43% (186 mg); White solid; mp: 100–102 °C; ^1^H NMR (200 MHz, CDCl_3_): *δ* = 8.01–7.14 (m, 11H, 10 × ArH, NH), 4.95 (t, *J* = 6.2 Hz, 1H, CH), 3.48 (s, 3H, OCH_3_), 3.25 (t, *J* = 7.5 Hz, 2H, PhCH_2_), 2.44–2.12 (m, 2H, C*H_2_*CH); ^13^C NMR (50 MHz, CDCl_3_): *δ* = 167.6, 150.3, 143.8 (q, *J* = 2.3 Hz), 136.7, 133.8, 131.6, 130.8, 128.8, 127.0, 126.0, 125.9, 125.52, 125.47, 123.4, 120.6 (q, *J* = 254.9 Hz), 119.9, 119.1, 114.0, 87.7, 55.3, 36.2, 28.3; ^19^F NMR (188 MHz, CDCl_3_): *δ* = −16.2 (OCF_3_).

*N*-(1-Methoxy-3-(naphthalen-1-yl)propyl)thiazol-2-amine (**18e**) and *N*-(1-methoxy-3-phenylpropyl)thiazol-2-amine (**18f**) were isolated as a mixture of hemiaminal ether and aminothiazole derivative (**19e,f**), and used directly in the next step.

### 2.6. General Procedure for the Reduction of Hemiaminal Ethers to 2-Aminobenzothiazoles ***19a–h***

To a stirred solution of hemiaminal ethers **18a–h** (1.0 mmol) in absolute MeOH (1 mL), placed in a pressure vessel, NaBH_4_ (76 mg, 2.0 mmol) was added. The vessel was sealed, and the reaction mixture was left stirring at 80 °C for 1 h. The solvent was then evaporated under reduced pressure; the residue was dissolved in H_2_O (5 mL) and ethyl acetate (10 mL), transferred to a separating funnel and extracted with ethyl acetate (3 × 5 mL). Purification by flash chromatography, eluting with an appropriate mixture of EtOAc:petroleum ether (40–60 °C), afforded the desired product.

#### 2.6.1. N-(3-(Naphthalen-2-yl)Propyl)Benzo[d]Thiazol-2-Amine (**19a**, GK543)

Elution solvent system: EtOAc:petroleum ether (40–60 °C) 3:7; Yield 97% (308 mg); White solid; mp: 112–114 °C; ^1^H NMR (200 MHz, CDCl_3_): *δ* = 7.85–7.70 (m, 3H, 3 × ArH), 7.64–7.22 (m, 7H, 7 × ArH), 7.12–7.03 (m, 1H, ArH), 6.06 (br, 1H, NH), 3.47 (t, *J* = 7.0 Hz, 2H, CH_2_N), 2.90 (t, *J* = 7.6 Hz, 2H, PhCH_2_), 2.11 (quint, *J* = 7.3 Hz, 2H, CH_2_); ^13^C NMR (50 MHz, CDCl_3_): *δ* = 167.8, 152.4, 138.5, 133.5, 132.0, 130.3, 128.1, 127.6, 127.4, 127.0, 126.5, 126.0, 125.9, 125.3, 121.4, 120.8, 118.7, 45.0, 33.1, 30.9; HRMS (ESI) [M − H]^−^ *m*/*z*: 317.1112 (calculated for [C_20_H_17_N_2_S]^−^ 317.1118).

#### 2.6.2. N-(3-Phenylpropyl)Benzo[d]Thiazol-2-Amine (**19b**, GK562)

Elution solvent system: EtOAc:petroleum ether (40–60 °C) 2:8; Yield 90% (241 mg); White solid; mp: 99–102 °C; lit. [30] 101.2–102 °C; ^1^H NMR (200 MHz, CDCl_3_): *δ* = 7.63–7.48 (m, 2H, 2 × ArH), 7.42–6.99 (m, 7H, 7 × ArH), 6.76 (br, 1H, NH), 3.43 (t, *J* = 7.0 Hz, 2H, CH_2_N), 2.74 (t, *J* = 7.6 Hz, 2H, PhCH_2_), 2.04 (quint, *J* = 7.3 Hz, 2H, CH_2_); ^13^C NMR (50 MHz, CDCl_3_): *δ* = 168.2, 152.1, 140.9, 130.0, 128.4, 128.3, 126.0, 125.9, 121.2, 120.8, 118.3, 45.1, 32.9, 30.9; HRMS (ESI) [M + H]^+^ *m*/*z*: 269.112 (calculated for [C_16_H_17_N_2_S]^+^ 269.1107).

#### 2.6.3. N-(3-(4-Methoxyphenyl)Propyl)Benzo[d]Thiazol-2-Amine (**19c**)

Elution solvent system: EtOAc:petroleum ether (40–60 °C) 3:7; Yield 97% (289 mg); White solid; mp: 104–106 °C; ^1^H NMR (200 MHz, CDCl_3_): *δ* = 7.60–6.73 (m, 9H, 8 × ArH, NH), 3.76 (s, 3H, CH_3_), 3.39 (t, *J* = 7.0 Hz, 2H, CH_2_N), 2.65 (t, *J* = 7.6 Hz, 2H, PhCH_2_), 2.04–1.89 (m, 2H, CH_2_); ^13^C NMR (50 MHz, CDCl_3_): *δ* = 168.2, 157.8, 152.1, 132.9, 130.0, 129.2, 125.9, 121.2, 120.8, 118.3, 113.7, 55.1, 45.0, 32.0, 31.1; HRMS (ESI) [M + H]^+^ *m*/*z*: 299.1203 (calculated for [C_17_H_19_N_2_OS]^+^ 299.1213).

#### 2.6.4. N-(3-(Naphthalen-1-yl)Propyl)Benzo[d]Thiazol-2-Amine (**19d**)

Elution solvent system: EtOAc:petroleum ether (40–60 °C) 3:7; Yield 80% (254 mg); White solid; mp: 134–137 °C; ^1^H NMR (200 MHz, CDCl_3_): *δ* = 8.01–7.68 (m, 3H, 3 × ArH), 7.63–7.19 (m, 7H, 7 × ArH), 7.14–7.06 (m, 1H, ArH), 6.77 (br, 1H, NH), 3.51 (t, *J* = 7.0 Hz, 2H, CH_2_N), 3.19 (t, *J* = 7.7 Hz, 2H, PhCH_2_), 2.16 (quint, *J* = 7.3 Hz, 2H, CH_2_); ^13^C NMR (50 MHz, CDCl_3_): *δ* = 167.8, 151.5, 137.0, 133.9, 131.6, 129.8, 128.8, 126.9, 126.1, 126.0, 125.54, 125.51, 123.5, 121.6, 120.9, 118.5, 45.4, 30.2, 30.1; HRMS (ESI) [M + H]^+^ *m*/*z*: 319.1264 (calculated for [C_20_H_19_N_2_S]^+^ 319.1263).

#### 2.6.5. N-(3-(Naphthalen-1-yl)Propyl)Thiazol-2-Amine (**19e**)

Elution solvent system: EtOAc:petroleum ether (40–60 °C) 4:6; Yield 96% (257 mg); White solid; mp: 94–97 °C; ^1^H NMR (200 MHz, CDCl_3_): *δ* = 8.06–7.97 (m, 1H, ArH), 7.91–7.82 (m, 1H, ArH), 7.78–7.69 (m, 1H, ArH), 7.56–7.31 (m, 4H, 4 × ArH), 7.12 (d, *J* = 3.6 Hz, 1H, ArH), 6.47 (d, *J* = 3.6 Hz, 1H, ArH), 6.31 (br, 1H, NH), 3.36 (t, *J* = 7.0 Hz, 2H, CH_2_N), 3.20 (t, *J* = 7.7 Hz, 2H, PhCH_2_), 2.14 (quint, *J* = 7.3 Hz, 2H, CH_2_); ^13^C NMR (50 MHz, CDCl_3_): *δ* = 171.0, 138.8, 137.2, 133.8, 131.6, 128.7, 126.8, 126.0, 125.8, 125.5, 123.5, 105.9, 45.9, 30.1, 29.9; HRMS (ESI) [M + H]^+^ *m*/*z*: 269.1102 (calculated for [C_16_H_17_N_2_S]^+^ 269.1107).

#### 2.6.6. N-(3-Phenylpropyl)Thiazol-2-Amine (**19f**)

Elution solvent system: EtOAc:petroleum ether (40–60 °C) 3:7; Yield 52% (113 mg); Pale white solid; mp: 83–84 °C; ^1^H NMR (400 MHz, CDCl_3_): *δ* = 7.37–7.21 (m, 5H, 5 × ArH), 7.13 (d, *J* = 3.6 Hz, 1H, ArH), 6.68 (br, 1H, NH), 6.49 (d, *J* = 3.6 Hz, 1H, ArH), 3.31 (t, *J* = 6.9 Hz, 2H, CH_2_N), 2.77 (t, *J* = 7.6 Hz, 2H, PhCH_2_), 2.04 (quint, *J* = 7.3 Hz, 2H, CH_2_); ^13^C NMR (100 MHz, CDCl_3_): *δ* = 171.0, 141.1, 138.6, 128.4, 128.3, 125.9, 105.8, 45.6, 33.0, 30.6; HRMS (ESI) [M + H]^+^ *m*/*z*: 219.0952 (calculated for [C_12_H_15_N_2_S]^+^ 219.0950).

#### 2.6.7. N-(3-(Naphthalen-2-yl)Propyl)-6-(Trifluoromethoxy)Benzo[d]Thiazol-2-Amine (**19g**)

Elution solvent system: EtOAc:petroleum ether (40–60 °C) 25:75; Yield 96% (386 mg); White solid; mp: 150–152 °C; ^1^H NMR (400 MHz, CDCl_3_): *δ* = 7.86–7.73 (m, 3H, 3 × ArH), 7.62 (s, 1H, ArH), 7.52–7.40 (m, 4H, 4 × ArH), 7.32 (d, *J* = 8.4 Hz, 1H, ArH), 7.13 (d, *J* = 8.9 Hz, 1H, ArH), 6.01 (br, 1H, NH), 3.47 (t, *J* = 7.0 Hz, 2H, CH_2_N), 2.91 (t, *J* = 7.5 Hz, 2H, PhCH_2_), 2.13 (quint, *J* = 7.1 Hz, 2H, CH_2_); ^13^C NMR (100 MHz, CDCl_3_): *δ* = 168.3, 151.0, 143.6 (q, *J* = 1.6 Hz), 138.3, 133.6, 132.1, 130.9, 128.2, 127.6, 127.4, 126.9, 126.5, 126.1, 125.4, 120.6 (q, *J* = 254.9 Hz), 119.7, 118.9, 114.0, 45.1, 33.1, 30.8; ^19^F NMR (377 MHz, CDCl_3_): *δ* = −58.2 (OCF_3_); HRMS (ESI) [M + H]^+^ *m*/*z*: 403.1087 (calculated for [C_21_H_18_F_3_N_2_OS]^+^ 403.1086).

#### 2.6.8. N-(3-(Naphthalen-1-yl)Propyl)-6-(Trifluoromethoxy)Benzo[d]Thiazol-2-Amine (**19h**)

Elution solvent system: EtOAc:petroleum ether (40–60 °C) 25:75; Yield 35% (141 mg); White solid; mp: 115–116 °C; ^1^H NMR (400 MHz, CDCl_3_): *δ* = 8.02–7.97 (m, 1H, ArH), 7.90–7.84 (m, 1H, ArH), 7.74 (d, *J* = 8.2 Hz, 1H, ArH), 7.50–7.31 (m, 6H, 6 × ArH), 7.14 (d, *J* = 8.8 Hz, 1H, ArH), 6.14 (br, 1H, NH), 3.51 (t, *J* = 7.0 Hz, 2H, CH_2_N), 3.20 (t, *J* = 7.6 Hz, 2H, PhCH_2_), 2.17 (quint, *J* = 7.1 Hz, 2H, CH_2_); ^13^C NMR (100 MHz, CDCl_3_): *δ* = 168.4, 151.0, 143.5 (q, *J* = 2.0 Hz) 136.9, 134.0, 131.7, 130.9, 128.9, 127.0, 126.04, 125.96, 125.6, 125.5, 123.4, 120.6 (q, *J* = 254.9 Hz), 119.7, 118.8, 114.0, 45.4, 30.2, 30.1; ^19^F NMR (377 MHz, CDCl_3_): *δ* = −58.2 (OCF_3_); HRMS (ESI) [M + H]^+^ *m*/*z*: 403.1086 (calculated for [C_21_H_18_F_3_N_2_OS]^+^ 403.1086).

### 2.7. General Procedure for the Synthesis of 2-Aminobenzoxazoles ***23a–d***

To a stirred solution of amine **22a–d** (1.0 mmol) in acetonitrile (1 mL), acetic acid (150 mg, 2.5 mmol), *tert*-butyl hydroperoxide (TBHP) 70% (0.16 mL, 1.25 mmol), tetrabutylammonium iodide (TBAI, 15 mg, 0.04 mmol) and benzoxazole (99 mg, 0.83 mmol) were added. The solution was stirred at 40–50 °C, for 24 h, and then, the mixture was quenched by the addition of an aqueous solution of 10% Na_2_S_2_O_3_ (10 mL) and a saturated aqueous solution of NaHCO_3_ (15 mL). The aqueous layer was extracted with CH_2_Cl_2_ (3 × 20 mL). The combined organic layers were collected and dried over Na_2_SO_4_, and the solvent was evaporated under reduced pressure. Purification by flash chromatography, eluting with the appropriate mixture of solvents, provided the desired product.

#### 2.7.1. N-(3-(Naphthalen-2-yl)Propyl)Benzo[d]Oxazol-2-Amine (**23a**)

Elution solvent system: EtOAc:petroleum ether (40–60 °C) 2:8 to 3:7; Yield 24% (72 mg); White solid; mp: 128–130 °C; ^1^H NMR (200 MHz, CDCl_3_): *δ* = 7.86–7.69 (m, 3H, 3 × ArH), 7.62 (s, 1H, ArH), 7.50–6.98 (m, 7H, 7 × ArH), 5.29 (br, 1H, NH), 3.61–3.47 (m, 2H, CH_2_N), 2.90 (t, *J* = 7.4 Hz, 2H, PhCH_2_), 2.11 (quint, *J* = 7.2 Hz, 2H, CH_2_); ^13^C NMR (50 MHz, CDCl_3_): *δ* = 162.7, 142.9, 138.5, 133.5, 132.0, 128.1, 127.6, 127.4, 127.0, 126.5, 126.0, 125.3, 123.9, 120.8, 116.2, 108.7, 42.6, 33.1, 31.1; HRMS (ESI) [M + H]^+^ *m*/*z*: 303.1487 (calculated for [C_20_H_19_N_2_O]^+^ 303.1492).

#### 2.7.2. N-(3-(4-Methoxyphenyl)Propyl)Benzo[d]Oxazol-2-Amine (**23b**)

Elution solvent system: EtOAc:petroleum ether (40–60 °C) 1:9 to 3:7; Yield 16% (45 mg); White solid; mp: 85–87 °C; ^1^H NMR (200 MHz, CDCl_3_): *δ* = 7.40–6.93 (m, 6H, 6 × ArH), 6.83–6.79 (m, 2H, 2 × ArH), 5.68 (br, 1H, NH), 3.77 (s, 3H, CH_3_), 3.49 (t, *J* = 6.9 Hz, 2H, CH_2_N), 2.68 (t, *J* = 7.6 Hz, 2H, PhCH_2_), 1.98 (quint, *J* = 7.3 Hz, 2H, CH_2_); ^13^C NMR (50 MHz, CDCl_3_): *δ* = 161.8, 157.9, 148.2, 142.0, 133.0, 129.2, 124.0, 120.9, 116.0, 113.9, 108.8, 55.2, 42.5, 32.0, 31.4; HRMS (ESI) [M + H]^+^ *m*/*z*: 283.1450 (calculated for [C_17_H_19_N_2_O_2_]^+^ 283.1441); HRMS (ESI) [M + Na]^+^ *m*/*z*: 305.1263 (calculated for [C_17_H_18_N_2_NaO_2_]^+^ 305.1260).

#### 2.7.3. N-(2-(Naphthalen-2-yloxy)Ethyl)Benzo[d]Oxazol-2-Amine (**23c**)

Elution solvent system: CH_2_Cl_2_:MeOH 100:0 to 95:5; Yield 52% (158 mg); Pale white solid; mp: 152–153 °C; ^1^H NMR (200 MHz, CDCl_3_): *δ* = 7.81–7.64 (m, 3H, 3 × ArH), 7.47–7.01 (m, 8H, 8 × ArH), 4.31 (t, *J* = 4.9 Hz, 2H, OCH_2_), 3.96 (t, *J* = 4.9 Hz, 2H, CH_2_N); ^13^C NMR (50 MHz, CDCl_3_): *δ* = 156.2, 141.6, 134.3, 129.6, 129.1, 127.6, 126.8, 126.5, 124.2, 123.9, 121.3, 118.5, 116.2, 109.0, 106.8, 66.2, 42.6; HRMS (ESI) [M + H]^+^ *m*/*z*: 305.1284 (calculated for [C_19_H_17_N_2_O_2_]^+^ 305.1285).

#### 2.7.4. N-(2-(Naphthalen-1-yloxy)Ethyl)Benzo[d]Oxazol-2-Amine (**23d**)

Elution solvent system: CH_2_Cl_2_:MeOH 100:0 to 95:5; Yield 45% (137 mg); Pale white solid; mp: 164–166 °C; ^1^H NMR (200 MHz, CDCl_3_): *δ* = 8.23–8.19 (m, 1H, ArH), 7.82–7.77 (m, 1H, ArH), 7.53–7.01 (m, 8H, 8 × ArH), 6.81–6.77 (m, 1H, ArH), 4.36 (t, *J* = 5.0 Hz, 2H, OCH_2_), 4.03 (t, *J* = 5.0 Hz, 2H, CH_2_N); ^13^C NMR (50 MHz, CDCl_3_): *δ* = 153.9, 148.5, 142.3, 134.5, 127.5, 126.5, 125.7, 125.3, 124.1, 121.6, 121.2, 120.9, 116.4, 108.9, 104.8, 66.6, 42.7; HRMS (ESI) [M + H]^+^ *m*/*z*: 305.1278 (calculated for [C_19_H_17_N_2_O_2_]^+^ 305.1285); HRMS (ESI) [M + Na]^+^ *m*/*z*: 327.1115 (calculated for [C_19_H_16_N_2_NaO_2_]^+^ 327.1104).

### 2.8. General Procedure for the Synthesis of 2-Aminobenzothiazoles ***25a,b***

To a stirred solution of bromides **24a,b** (1.0 mmol) in *N,N*-dimethylformamide (DMF, 20 mL), **3a** (180 mg, 1.2 mmol) and K_2_CO_3_ (166 mg, 1.2 mmol) were added, and the reaction mixture was refluxed for 3 h at 150 °C. Then, water (20 mL) was added, the mixture was extracted with ethyl acetate (3 × 50 mL), and the combined organic layers were washed with H_2_O (3 × 75 mL). The organic layer was collected and dried over Na_2_SO_4_, and the solvent was evaporated under reduced pressure. Purification by gradient flash chromatography, eluting with a mixture of CH_2_Cl_2_:MeOH (100:0 to 95:5), provided the desired product.

#### 2.8.1. N-(2-(Naphthalen-2-yloxy)Ethyl)Benzo[d]Thiazol-2-Amine (**25a**)

Elution solvent system: CH_2_Cl_2_:MeOH 100:0 to 95:5; Yield 27% (86 mg); White solid; mp: 170–172 °C; ^1^H NMR (200 MHz, DMSO-d_6_): *δ* = 8.37 (t, *J* = 5.2 Hz, 1H, NH), 7.87–7.75 (m, 3H, 3 × ArH), 7.68 (d, *J* = 7.6 Hz, 1H, ArH), 7.49–7.17 (m, 6H, 6 × ArH), 7.07–6.99 (m, 1H, ArH), 4.31 (t, *J* = 5.4 Hz, 2H, OCH_2_), 3.90–3.78 (m, 2H, CH_2_N); ^13^C NMR (50 MHz, DMSO-d_6_): *δ* = 166.1, 156.3, 152.5, 134.3, 130.4, 129.4, 128. 6, 127.5, 126.7, 126.4, 125.6, 123.7, 121.1, 121.00, 118.7, 118.1, 106.8, 66.0, 43.2; HRMS (ESI) [M + H]^+^ *m*/*z*: 321.1050 (calculated for [C_19_H_17_N_2_OS]^+^ 321.1056).

#### 2.8.2. N-(2-(Naphthalen-1-yloxy)Ethyl)Benzo[d]Thiazol-2-Amine (**25b**)

Elution solvent system: CH_2_Cl_2_:MeOH 100:0 to 98:2; Yield 32% (102 mg); White solid; mp: 170–173 °C; ^1^H NMR (200 MHz, DMSO-d_6_): *δ* = 8.41 (t, *J* = 5.6 Hz, 1H, NH), 8.26 (d, *J* = 7.9 Hz, 1H, ArH), 7.85 (d, *J* = 7.9 Hz, 1H, ArH), 7.68 (d, *J* = 7.7 Hz, 1H, ArH), 7.54–7.20 (m, 6H, 6 × ArH), 7.07–6.98 (m, 2H, 2 × ArH), 4.34 (t, *J* = 5.2 Hz, 2H, OCH_2_), 4.00–3.86 (m, 2H, CH_2_N); ^13^C NMR (50 MHz, DMSO-d_6_): *δ* = 166.3, 154.0, 152.5, 134.0, 130.4, 127.4, 126.5, 126.2, 125.6, 125.1, 124.9, 122.0, 121.0, 121.0, 120.1, 118.1, 105.2, 66.6, 43.3; HRMS (ESI) [M + H]^+^ *m*/*z*: 321.1054 (calculated for [C_19_H_17_N_2_OS]^+^ 321.1056).

### 2.9. Biological Assays

#### 2.9.1. Cell Culture

Rat renal mesangial cells (clone MZ B1), isolated and characterized as previously described [31], were cultivated in RPMI 1640 medium supplemented with 10% fetal bovine serum; 10 mM 4-(2-hydroxyethyl)-1-piperazineethanesulfonic acid (HEPES), pH 7.4; 6 µg/mL of bovine insulin; 5 µg/mL of transferrin; 5 nM sodium selenite; 100 units/mL of penicillin; and 100 µg/mL of streptomycin. The cells were incubated for 4 h in Dulbecco’s modified Eagle’s medium (DMEM) containing 10 mM HEPES, pH 7.4, and 0.1 mg/mL of fatty acid-free bovine serum albumin (BSA), prior to stimulation.

#### 2.9.2. Quantification of PGE_2_

Confluent mesangial cells in 24-well plates were pretreated for 20 min with varying concentrations of inhibitors and then stimulated for 24 h in a total volume of 400 µL of DMEM containing 0.1 mg/mL of BSA, in the absence or presence of 1 nM interleukin 1β (IL-1) plus 5 μM forskolin (Fk). Thereafter, the supernatants were collected and centrifuged for 5 min at 1000× *g*. The PGE_2_ in the supernatant was quantified using an enzyme-linked immunoassay (Enzo Life Sciences, Lörrach, Germany) following the manufacturer’s recommendations.

#### 2.9.3. Statistical Analysis

Statistical analysis of the data was performed using one-way analysis of variance (ANOVA) followed by a Bonferroni’s post hoc test for multiple comparisons (GraphPad Prism 8.4.3., San Diego, CA, USA). The half maximal effective concentrations (EC_50_) of the inhibitors were calculated using the same software.

#### 2.9.4. In Vitro PLA_2_ Activity Assay

A previously described lipidomics-based mixed micelle assay was used to determine the activities of human recombinant group VIA phospholipase A_2_ (GVIA iPLA_2_), group IVA cytosolic phospholipase A_2_ (GIVA cPLA_2_) and group V secreted phospholipase A_2_ (GV sPLA_2_) [32,33]. The substrate for GVIA iPLA_2_ and GV sPLA_2_ consisted of 100 μM 1-palmitoyl-2-arachidonoyl-phosphatidylcholine (PAPC), 400 μM C12E8 surfactant and 2.5 μM 17:0 lysophosphatidylcholine (LPC) internal standard. For GIVA cPLA_2_, the substrate consisted of 97 μM PAPC, 3 μM porcine brain phosphatidylinositol 4,5-bisphosphate (PI(4,5)P_2_), 400 μM C12E8 surfactant and 2.5 μM 17:0 LPC internal standard. The buffer for GIVA cPLA_2_ contained 100 mM HEPES at pH 7.5, 90 μM CaCl_2_ and 2 mM dithiothreitol (DTT). For GVIA iPLA_2_, the buffer consisted of 100 mM HEPES at pH 7.5, 2 mM adenosine triphosphate (ATP) and 4 mM DTT. Finally, the buffer for GV sPLA_2_ contained 50 mM tris(hydroxymethyl)aminomethane hydrochloride (Tris-HCl) at pH 8.0 and 5 mM CaCl_2_. The enzymatic reaction was performed in a 96-well plate using a Benchmark Scientific H5000-H MultiTherm heating shaker for 30 min at 40 °C. Each reaction was quenched with 120 μL of MeOH/acetonitrile (80/20, *v*/*v*). The samples were analyzed using an HPLC−MS system, which was constituted of a Shimadzu SCL-10A system controller with two LC-10AD liquid pumps connected to an Analytical Sales & Products column controller instrument, a CTC Analytics PAL autosampler platform and an AB Sciex 4000 QTRAP triple quadrupole/linear ion trap hybrid mass spectrometer.

#### 2.9.5. Rat-Paw Carrageenan-Induced-Edema Assay

The anti-inflammatory activities of selected aminobenzothiazole derivatives were determined by employing the rat-paw carrageenan-induced-edema assay, as previously described [18]. Only male animals (180–220 g body weight) were used. Each group was composed of five animals. The animals were divided into three five-membered groups, and all the tested compounds were suspended in water (0.01 mmol/mL/kg body weight), with a few drops of Tween 80, and ground in a mortar before being administered intraperitoneally simultaneously. After treatment with the tested compounds in group 1, a 2nd group was used as a positive control (indomethacin at 0.01 mmol/mL/kg body weight, i.p.), and another group (3rd) served as the control in which water was administered (negative control). The compounds were injected intraperitoneally at the same time as carrageenan was given by intradermal injection. The rats were euthanized 3.5 h post-injection. The weight of the uninjected with carrageenan paw was subtracted from the weight of the injected paw for each animal. The change in paw weight for the treated animals was compared to that for the control animals and expressed as the percent inhibition of edema. The values of CPE % are the means from three different experiments with standard errors of the mean less than 10%. The statistical significance of the results was established with Student’s *t*-test, for * *p <* 0.01 and ** *p <* 0.05. All the animal experiments performed in the manuscript were conducted in compliance with institutional guidelines. Our studies were in accordance with recognized guidelines on animal experimentation (guidelines for the care and use of laboratory animals published by the Greek Government 160/1991, based on EU regulations 86/609). The rats were kept in the Centre of the School of Veterinary Medicine (EL54 BIO42), Aristotle University of Thessaloniki, which is registered by the official state veterinary authorities (presidential degree 56/2013, in harmonization with the European Directive 2010/63/EEC). The experimental protocols were approved by the Animal Ethics Committee of the Prefecture of Central Macedonia (no. 270079/2500).

## 3. Results and Discussion

### 3.1. Synthesis of Inhibitors

**Scheme 1:**
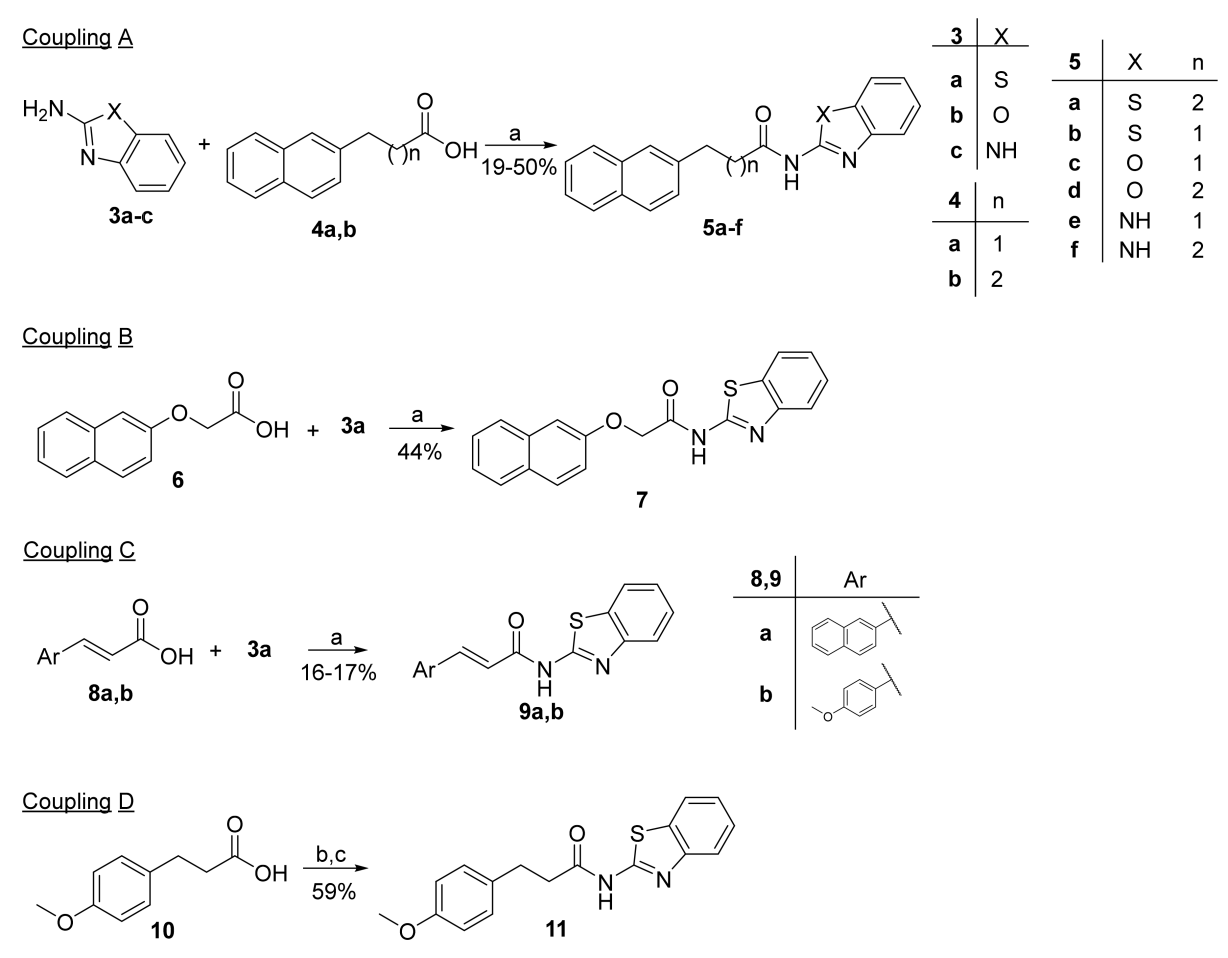
Coupling for the synthesis of *N*-acylated 2-aminobenzothiazoles and related heterocycles. (a) EDCI·HCl, Et_3_N, HOBt, dry CH_2_Cl_2_, 0 °C to rt; (b) SOCl_2_; (c) **3a**, Et_3_N, CH_2_Cl_2_.

A series of *N*-acylated 2-aminobenzothiazoles, benzoxazoles and benzimidazoles were synthesized by the general coupling procedures depicted in Scheme 1. *N*-Acylated 2-aminobenzothiazoles, as well as the corresponding benzoxazoles and benzimidazoles (**5a–f**), were prepared by coupling the appropriate carboxylic acid **4a,b** with 2-aminobenzothiazole (**3a**), 2-aminobenzoxazole (**3b**) or 2-aminobenzimidazole (**3c**) (Coupling A, Scheme 1) using EDCI·HCl as the coupling reagent, in the presence of HOBt. In the same manner, carboxylic acid **6** or α,β-unsaturated carboxylic acids **8a,b** were coupled with **3a** to produce compounds **7** and **9a,b** (Couplings B and C, Scheme 1). For the synthesis of **11**, carboxylic acid **10** was converted to the corresponding chloride and reacted with **3a** (Coupling D, Scheme 1).

**Scheme 2:**
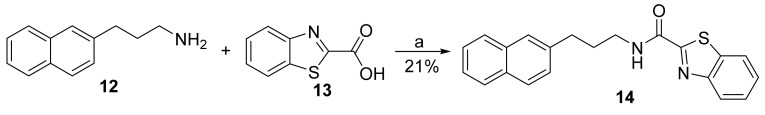
Coupling using benzothiazole-2-carboxylic acid (**13**). (a) EDCI·HCl, Et_3_N, HOBt, dry CH_2_Cl_2_, 0 °C to rt.

Compound **14** was the product of a coupling between benzothiazole-2-carboxylic acid (**13**) and amine **12**, by the EDCI/HOBt method (Scheme 2).

**Scheme 3:**
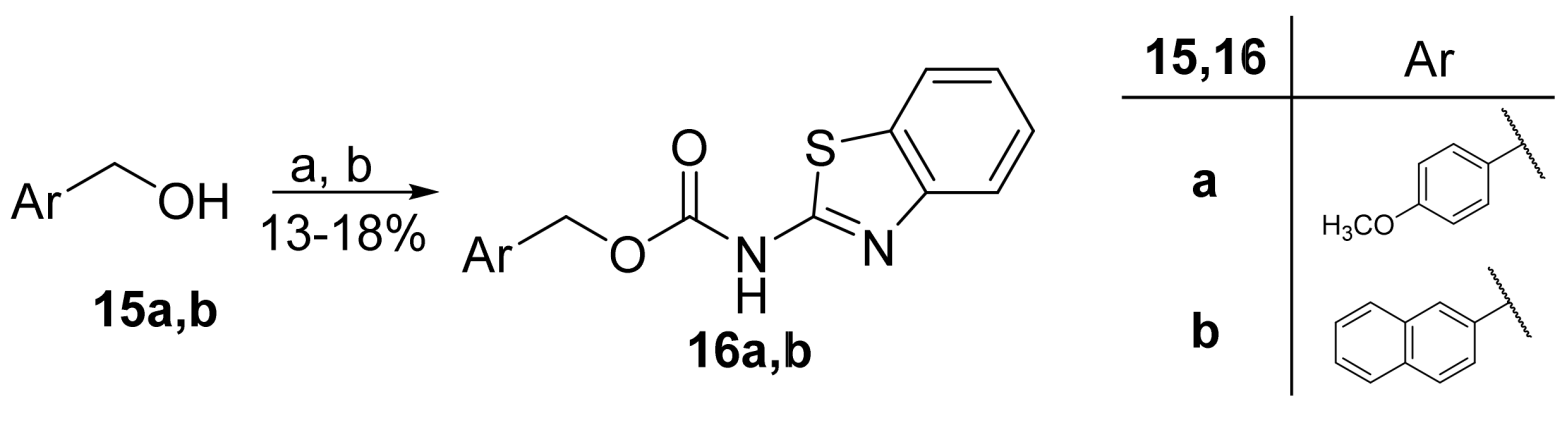
Synthesis of carbamate derivatives. (a) Triphosgene, Et_3_N, CH_2_Cl_2_, 0 °C to rt; (b) **3a**, 4-DMAP, Et_3_N, THF, reflux.

Carbamates **16a,b** were prepared in a two-step reaction from benzylic alcohols **15a,b**. The first step included conversion into chloroformates by treatment with triphosgene, which then reacted with 2-aminobenzothiazole (**3a**) in the presence of triethylamine and 4-DMAP (Scheme 3).

**Scheme 4:**
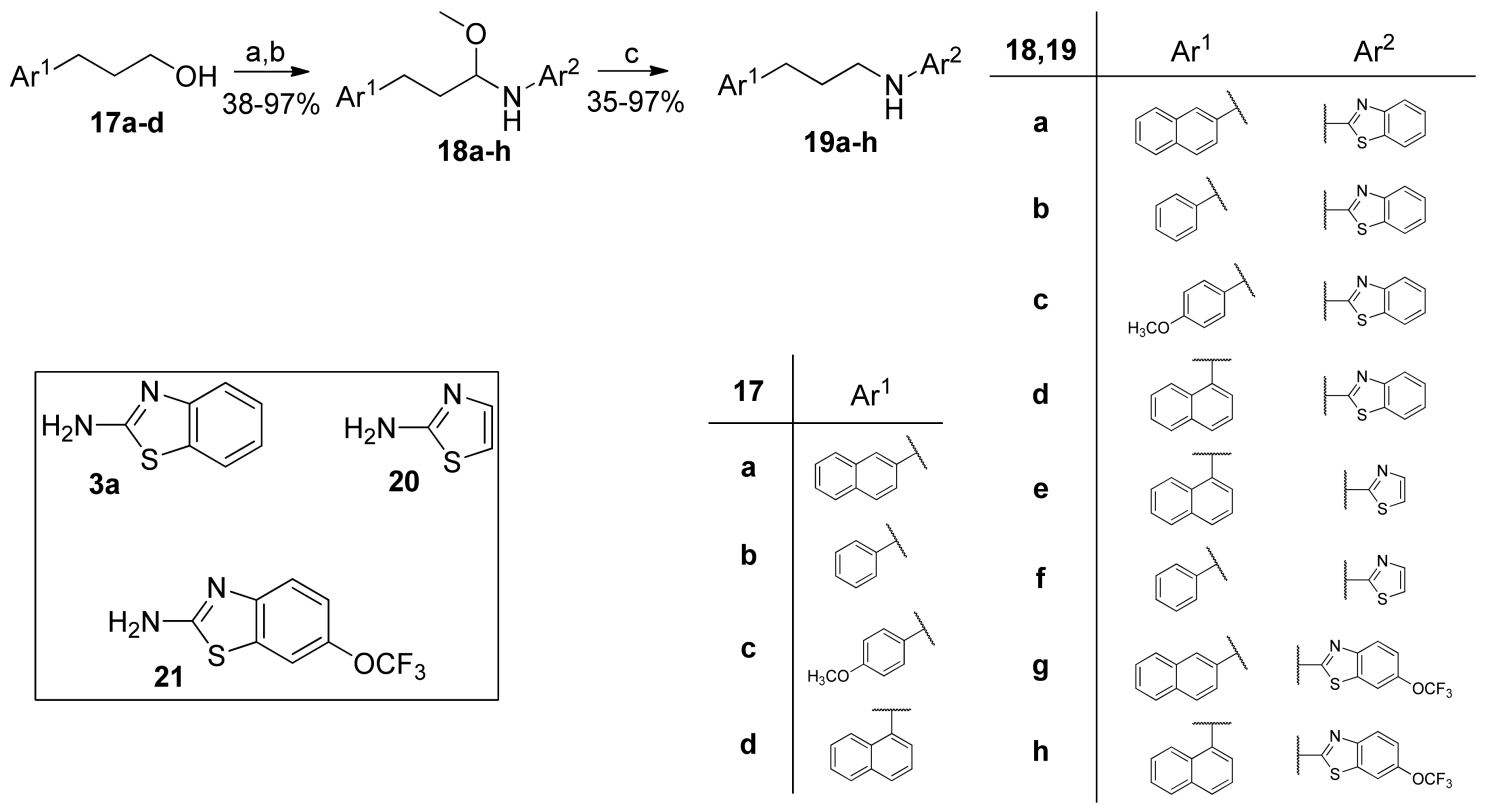
Synthesis of *N*-alkylated 2-aminobenzothiazoles and thiazoles via reductive amination. (a) C_6_H_5_I(O_2_CCH_3_)_2_, 10% cat. TEMPO, dry CH_2_Cl_2_; (b) i. 2-aminobenzothiazole (**3a**), 2-aminothiazole (**20**) or 2-amino-6-(trifluoromethoxy)benzothiazole (**21**), Na_2_SO_4_, absolute MeOH; ii. NaBH_4_; (c) NaBH_4_, absolute MeOH, 80 °C.

*N*-Alkylated 2-aminobenzothiazoles and thiazoles were synthesized starting from primary alcohols **17a–d** through a reductive amination reaction, as shown in Scheme 4. Alcohols **17a–d** were oxidized to the corresponding aldehydes, which reacted with 2-aminobenzothiazole (**3a**), 2-aminothiazole (**20**) or 2-amino-3-(trifluoromethoxy)benzothiazole (**21**) to produce either hemiaminal ethers **18a–h** or, directly, the final amines **19a–h**. In most cases, the hemiaminal ethers were formed and could be isolated and studied for their inhibitory activity. The further heating of the hemiaminal ethers **18a–h** in a reductive environment afforded the target compounds (Scheme 4).

**Scheme 5:**
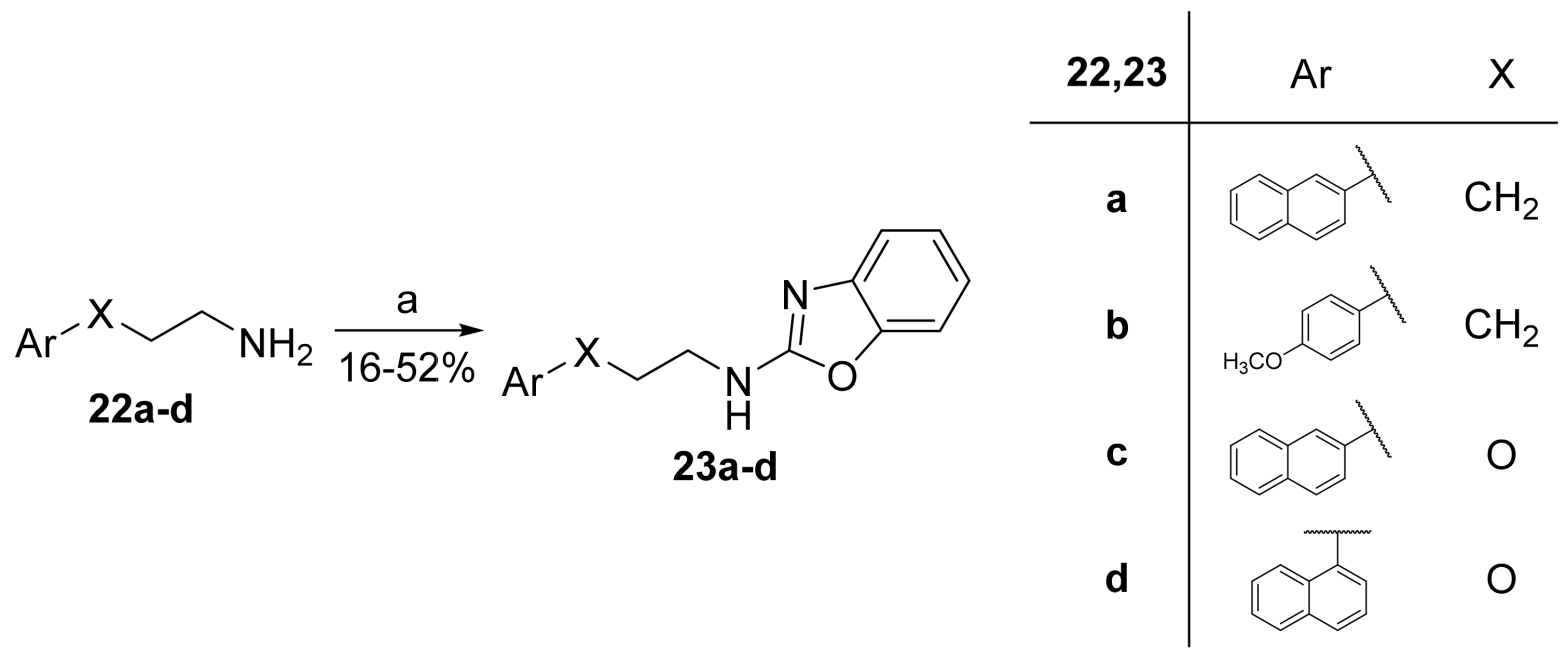
Benzoxazole amination. (a) TBAI, TBHP, CH_3_COOH, benzoxazole, MeCN, 50 °C.

*N*-Alkylated 2-aminobenzoxazoles **23a–d** were synthesized through a metal-free oxidative amination of benzoxazole with amines **22a–d**, using TBAI, TBHP and acetic acid [34], as depicted in Scheme 5.

**Scheme 6:**
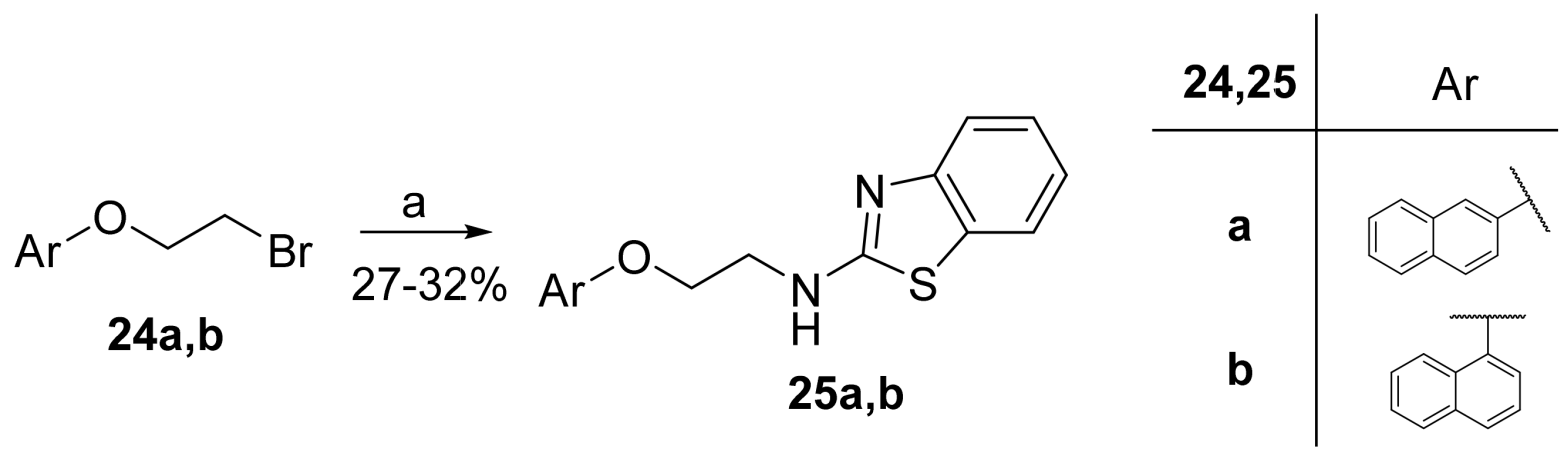
Synthesis of *N*-alkylated 2-aminobenzothiazoles **25a,b**. (a) **3a**, K_2_CO_3_, DMF, reflux.

*N*-Alkylated 2-aminobenzothiazoles **25a,b**, containing an oxygen atom attached to the naphthalene ring, were synthesized through the substitution of bromides **24a,b** by 2-aminobenzothiazole (**3a**), under alkaline conditions and reflux (Scheme 6).

### 3.2. Study of the Suppression of PGE_2_ Generation in Mesangial Cells

All the compounds synthesized were evaluated for their ability to suppress the production of PGE_2_, using renal mesangial cells as a model, as described in our previous studies [20,21]. As demonstrated in the past, mesangial cells are involved in various pathological processes, including inflammation of the renal glomerulus [35]. Upon the stimulation of rat renal mesangial cells with interleukin-1β (IL-1β) plus forskolin (Fk), a huge increase in PGE_2_ formation is observed, permitting the evaluation of synthetic compounds as inhibitors of this generation [20,21,35,36]. The results obtained for the effect of the newly synthesized compounds at a concentration of 3 µM are summarized in Table 1 and Table 2.

Initially, an analog of compound **1** (compound **14**, entry 1, Table 1), where the carbonyl group of **1** was incorporated into an amide bond, and two *N*-acylated derivatives of 2-aminobenzothiazole (**5a** and **5b** (GK510), entries 2 and 3, Table 1), where the naphthalene ring is situated at a distance corresponding to two or three carbon atoms away from the carbonyl group, were evaluated. Compound **14** exhibited weaker inhibitory activity (64%) in comparison to **1** (85%) [21]. Both compounds **5a** and **5b** inhibited the generation of PGE_2_ (50% and 96%, respectively); however, **5b** exhibited potent inhibition, indicating that the optimum distance between the naphthalene ring and the amide bond corresponds to two carbon atoms. The insertion of a double bond at the α,β-position (**9a**, entry 4, Table 1) or replacement of the amide functionality by a carbamate one (**16b**, entry 5, Table 1) destroyed the inhibitory activity (28% and 39%, respectively). The replacement of the benzothiazole group of **5a** and **5b** by a benzoxazole group led to active derivatives. The benzoxazole derivative **5d** (entry 6, Table 1), carrying the naphthalene group at a distance of three carbon atoms from the carbonyl group, exhibited higher inhibitory activity (73%) than the benzoxazole derivative **5c** (49%, entry 7, Table 1), with a shorter linker. When a benzimidazole group replaced the benzothiazole group of **5a** and **5b**, a remarkable decrease in activity was observed. Compound **5f** (entry 8, Table 1) inhibited it by 66%, while **5e** (entry 9, Table 1) inhibited it by 29%. The replacement of the naphthalene ring of **5b** by a p-methoxyphenyl ring (compound **11**, entry 10, Table 1) resulted in a decrease in inhibitory potency (80%). Similarly, derivatives carrying a p-methoxyphenyl ring, either **9b** (entry 11, Table 1) bearing an α,β-double bond or **16a** (entry 12, Table 1) bearing a carbamate group, presented very weak activity (25% and 15%, respectively). Finally, the insertion of an oxygen atom, replacing the methylene attached to the naphthalene ring (compound **7**, entry 13, Table 1), led, again, to a decrease in the inhibitory activity (41%).

Next, we explored the replacement of the amide bond of the previous compounds by either a hemiaminal ether or a methyleneamino functionality. Compound **18a** (entry 1, Table 2), where the carbonyl group of **5b** was replaced by a methoxy group (hemiaminal ether), was found to present 46% inhibitory activity. Interestingly, the *N*-alkylated 2-aminobenzothiazole derivative **19a** (GK543) (entry 2, Table 2) proved to be a potent inhibitor, causing 98% inhibition at 3 µM. Similar potent inhibitory activity (95%) was observed for the *N*-alkylated 2-aminobenzoxazole derivative **23a** (entry 3, Table 2). The insertion of an oxygen atom (compound **25a**, entry 4, Table 2), replacing the methylene group attached to the naphthalene ring, resulted in a considerable decrease in the inhibitory potency (63%), in comparison to **19a**. However, in the case of the benzoxazole derivatives, similar replacement (compound **23c**, entry 5, Table 2) led to a slight decrease (87%). Changing the position of the substituent on the naphthalene group from 2- to 1- (compound **19d**, entry 6, Table 2) caused a decrease in the activity (82%), in comparison to **19a**. An additional decrease was observed for compound **25b** (entry 7, Table 2), where an oxygen atom replaced a methylene of **19d**. The benzoxazole derivative **23d** (entry 8, Table 2), bearing an oxygen atom, caused an even weaker effect (45%).

Then, the naphthalene ring was replaced by either a p-methoxyphenyl or a phenyl ring. The hemiaminal ether **18c** (entry 9, Table 2) as well as **18b** (entry 10, Table 2) did not present any inhibitory activity. To the contrary, derivative **19c** (entry 11, Table 2) inhibited PGE_2_ generation by 62% and, gratifyingly, *N*-alkylated 2-aminobenzothiazole **19b** (GK562) (entry 12, Table 2) proved to potently suppress PGE_2_ generation (96%) at 3 μM. The benzoxazole derivative **23b** (entry 13, Table 2) exhibited a reduced activity (79%).

Two derivatives containing a thiazole ring replacing the benzothiazole ring were also studied. Compound **19e** (entry 14, Table 2) inhibited the activity by 88%, while compound **19f** (entry 15, Table 2) exhibited a weak effect (20%). Finally, two riluzole-based derivatives (**19** and **19h**, entries 16 and 17, respectively, Table 2) were evaluated and found to be inactive.

**Figure 2:**
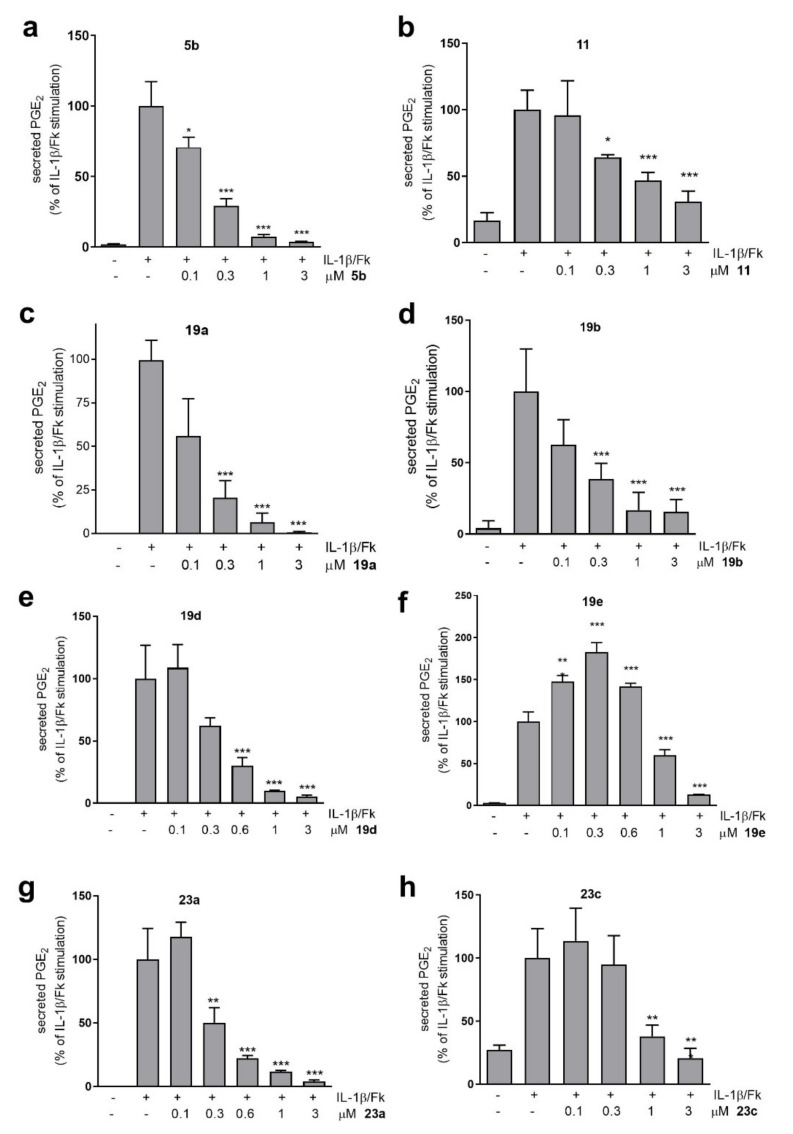
Effect of compounds **5b** (**a**), **11** (**b**), **19a** (**c**), **19b** (**d**), **19d** (**e**), **19e** (**f**), **23a** (**g**) and **23c** (**h**) on IL-1/Fk-stimulated PGE_2_ generation in rat mesangial cells. Cells were pretreated for 20 min with the indicated concentrations of inhibitors and then stimulated for 24 h in the absence (−) or presence (+) of 1 nM interleukin 1β (IL-1) plus 5 μM forskolin (Fk). PGE_2_ was quantified in supernatants using an enzyme-linked immunoassay, as described in the Experimental section. Data are presented as % of IL-1/Fk stimulation and are means ± S.D.s (n = 3). * *p* < 0.05, ** *p* < 0.01, and *** *p* < 0.001 were considered statistically significant when comparing with the IL-1/Fk-stimulated samples.

Taken together, eight novel compounds—*N*-acylated 2-aminobenzothiazoles **5b** and **11**; *N*-alkylated 2-aminobenzothiazoles **19a**, **19b** and **19d**; *N*-alkylated 2-aminobenzoxazoles **23a** and **23c**; and *N*-alkylated 2-aminothiazole **19e**—were identified to inhibit the generation of PGE_2_ at levels higher than 80% at 3 µM in rat renal mesangial cells. The effect of these compounds was further studied at various concentrations ranging from 0.1 to 3 µM, and the results are shown in Figure 2. The EC_50_ values are summarized in Table 1 and Table 2. *N*-Acylated 2-aminobenzothiazole **5b** and *N*-alkylated 2-aminobenzothiazoles **19a** and **19b** were found to exhibit the most potent inhibitory activity, with EC_50_ values of 173 nM, 118 nM and 177 nM, respectively. As concluded, either *N*-acylated- or *N*-alkylated 2-aminobenzothiazole derivatives were demonstrated to be better inhibitors of PGE_2_ generation than the corresponding benzoxazole derivatives. As shown in Figure 2, **19e** presented an unusual biphasic effect, as low concentrations enhanced PGE_2_, while at higher concentrations of 1 µM and 3 µM, PGE_2_ was efficiently reduced. The reason for this biphasic effect is presently unclear; however, NSAIDs have been reported to exhibit such biphasic effects [37].

### 3.3. Study of the In Vivo Anti-Inflammatory Activity

The rat-paw carrageenan-induced edema assay was employed as a model for acute inflammation to evaluate the anti-inflammatory activity of selected benzothiazole derivatives, as we have previously described for the evaluation of PLA_2_ inhibitors [18]. Two of the benzothiazole derivatives, presenting the most potent inhibitory activity in vitro (**5b**, EC_50_ 173 nM, and **19a**, EC_50_ 118 nM), as well as **19c**, presenting weaker activity (62% at 3 μM), were evaluated at a dose of 0.01 mmol/kg. Indomethacin was used in these experiments as a reference drug and led to 37.3% inhibition of inflammation at the same dose. The results for the in vivo anti-inflammatory activity of **5b**, **19a** and **19c** are presented in Table 3 and are in accordance with their in vitro inhibitory activity of PGE_2_ generation. Two novel benzothiazole derivatives synthesized in the present study, **5b** and **19a**, exhibited anti-inflammatory activity higher than that of indomethacin.

### 3.4. Inhibition of Phospholipases A_2_ by **19a**

**Figure 3:**
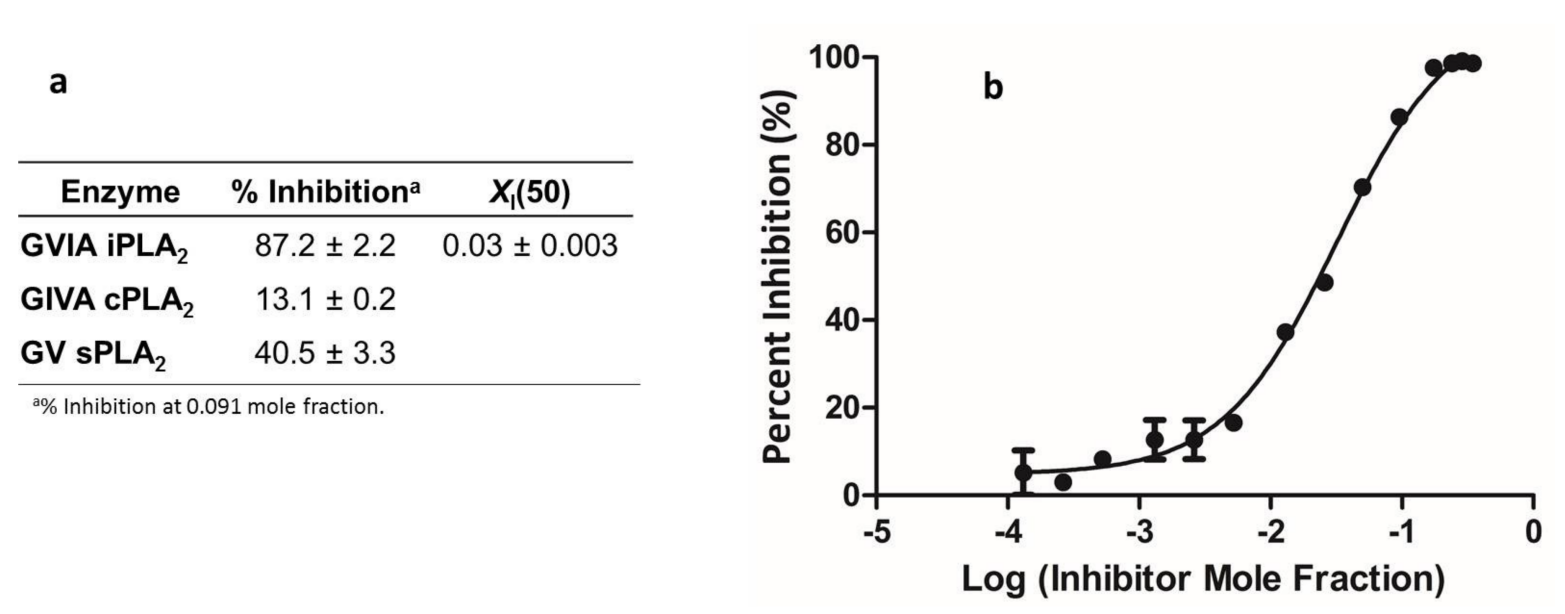
Inhibition of PLA_2_s by **19a** (**a**). Dose–response inhibition curve for GVIA iPLA_2_ for **19a** (**b**). The curve was generated using GraphPad Prism with a nonlinear regression targeted to a symmetrical sigmoidal curve based on plots of % inhibition vs. log(inhibitor concentration). The reported *X*_I_(50) value was calculated from the resultant plot.

To obtain insight as to the mechanism of action of compound **19a**, which was identified in the present study to exert the most potent in vitro and in vivo activity, its effect on NO formation in cytokine-stimulated rat mesangial cells and on the ability to inhibit PLA_2_ activity in vitro was studied. No effect on NO production was observed (Supplementary Materials, Figure S1), suggesting that **19a** is not involved in the NF-kB pathway and gene transcription [38]. We determined the ability of **19a** to inhibit three different human PLA_2_s, namely, cytosolic calcium-dependent PLA_2_ (GIVA cPLA_2_), secreted PLA_2_ (GV sPLA_2_) and calcium-independent PLA_2_ (GVIA iPLA_2_), utilizing a lipidomics assay, as previously described [32]. Compound **19a** was found to inhibit GVIA iPLA_2_ with an *X*_I_(50) value of 0.03, but did not inhibit GIVA cPLA_2_ or GV sPLA_2_ significantly (Figure 3). The *X*_I_(50) is the mole fraction of the inhibitor in the total substrate interface required to inhibit the enzyme activity by 50%. Although this inhibitory activity is not particularly potent, this finding suggests that the anti-inflammatory activity of **19a** may be attributed, at least in part, to the inhibition of GVIA iPLA_2_ and its specific interference with the prostaglandin pathway. We have previously shown that compound AX048 (ethyl 4-(2-oxohexadecanamido)butanoate) [17], which, in vivo, presents anti-hyperalgesic activity and inhibits spinal PGE_2_ release, inhibits GVIA iPLA_2_ in vitro with an *X*_I_(50) value of 0.027 (a value comparable to that measured for **19a**) and GIVA cPLA_2_ with an *X*_I_(50) value of 0.022 [17]. We have also shown that arachidonoyl trifluoromethyl ketone, a widely used inhibitor of GVIA iPLA_2_, inhibits the cytokine-stimulated generation of PGE_2_ in rat mesangial cells [36] and modulates allodynia after facial carrageenan injection in mice [39]. Furthermore, the selective GVIA iPLA_2_ inhibitor FKGK18 (1,1,1-trifluoro-6-(2-naphthalenyl)-2-hexanone) has been demonstrated to inhibit the generation of PGE_2_ in CD4^+^ T cells [40]. Taken together, the inhibition of PGE_2_ release and the anti-inflammatory activity of **19a** may be attributed, at least in part, to the inhibition of GVIA iPLA_2_. However, in addition, **19a** may exert its anti-inflammatory activity by acting on additional target(s).

## 4. Conclusions

We have developed various routes for the synthesis of *N*-acylated and *N*-alkylated 2-aminobenzothiazoles, and we have synthesized a series of such compounds and related benzoxazoles and benzimidazoles. All the compounds synthesized were evaluated for their ability to inhibit the cytokine-stimulated PGE_2_ release at a cellular level, employing a rat mesangial cell model. We have identified three novel compounds exhibiting potent inhibition of PGE_2_ generation. 2-Aminobenzothiazole acylated by a 3-(naphthalen-2-yl)propanoyl moiety (**5b**), as well as 2-aminobenzothiazole *N*-alkylated by a three carbon-atom chain carrying either a naphthalene (**19a**) or a phenyl (**19b**) ring, was found to suppress PGE_2_ formation with EC_50_ values of 173 nM, 118 nM and 177 nM, respectively. The inhibitors **5b** and **19a** were also found to exhibit anti-inflammatory activity greater than that of indomethacin in a rat-paw carrageenan-induced edema assay. The inhibition of PGE_2_ release and the anti-inflammatory activity of the most potent compound, **19a**, identified in the present study may be attributed, at least in part, to the inhibition of GVIA iPLA_2_. All the above findings suggest that *N*-acylated and *N*-alkylated 2-aminobenzothiazoles are potential novel leads for the development of agents inhibiting PGE_2_ generation.

## Figures and Tables

**Table 1:** Inhibition of PGE_2_ generation by *N*-acylated 2-aminobenzothiazoles and related heterocycles.

Entry	Compound	Structure	% Inhibition (at 3 μM)	EC_50_ (μM)
1	**14**	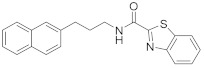	64	
2	**5a**	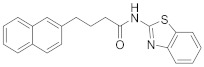	50	
3	**5b**	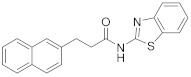	96	0.173
4	**9a**	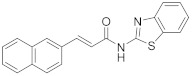	28	
5	**16b**	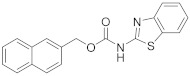	39	
6	**5d**	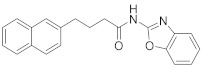	73	
7	**5c**	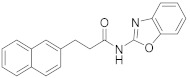	49	
8	**5f**	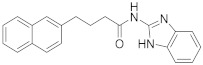	66	
9	**5e**	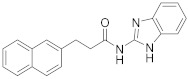	29	
10	**11**	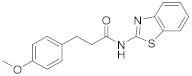	80	0.916
11	**9b**	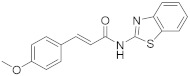	25	
12	**16a**	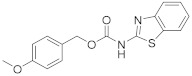	15	
13	**7**	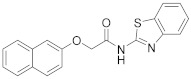	41	

**Table 2:** Inhibition of PGE_2_ generation by *N*-alkylated 2-aminobenzothiazoles and benzoxazoles.

Entry	Compound	Structure	% Inhibition (at 3 μM)	EC_50_ (μM)
1	**18a**	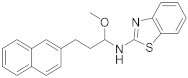	46	
2	**19a**	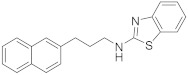	98	0.118
3	**23a**	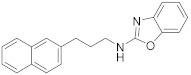	95	0.336
4	**25a**	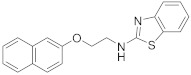	63	
5	**23c**	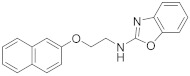	87	0.894
6	**19d**	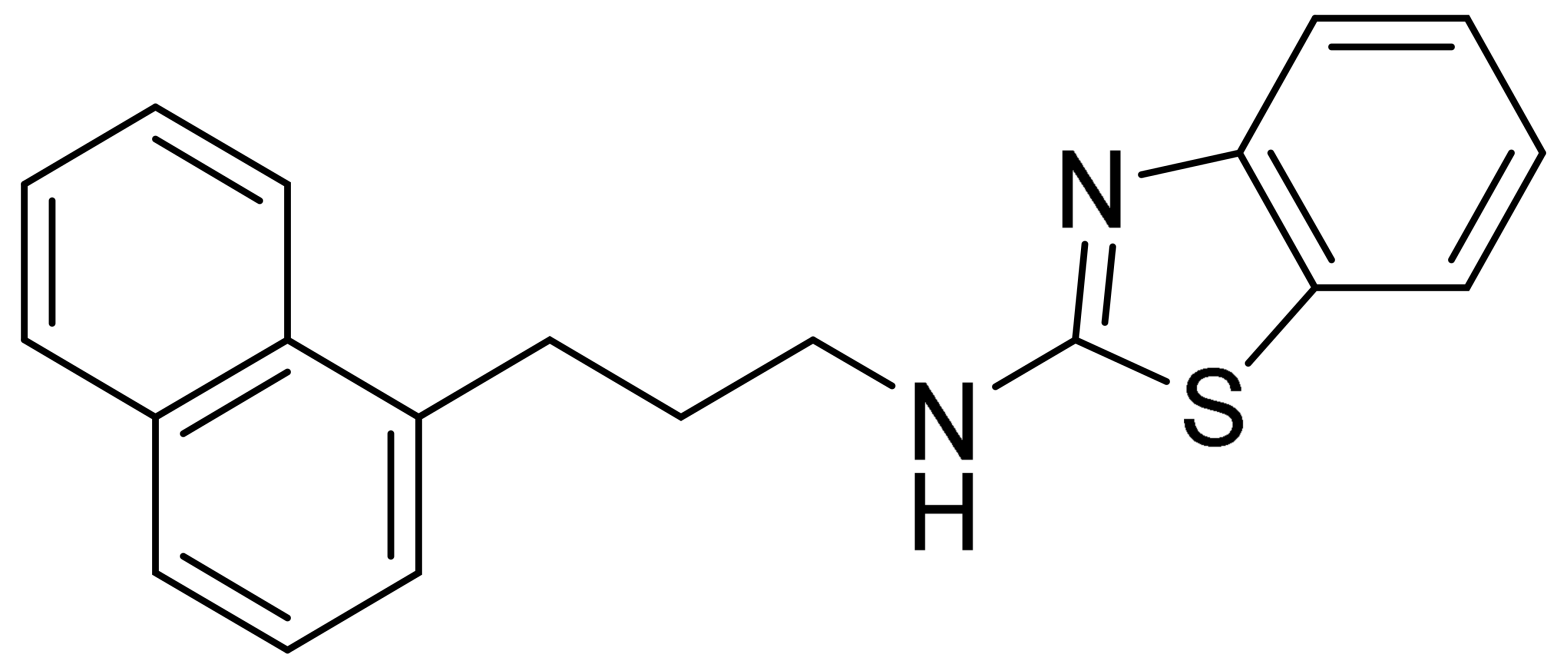	82	0.399
7	**25b**	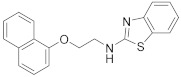	57	
8	**23d**	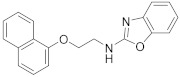	45	
9	**18c**	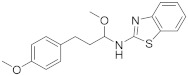	0	
10	**18b**	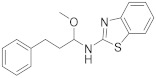	0	
11	**19c**	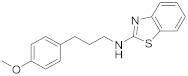	62	
12	**19b**	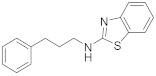	96	0.177
13	**23b**	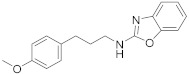	79	
14	**19e**	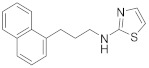	88	
15	**19f**	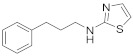	20	
16	**19g**	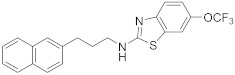	0	
17	**19h**	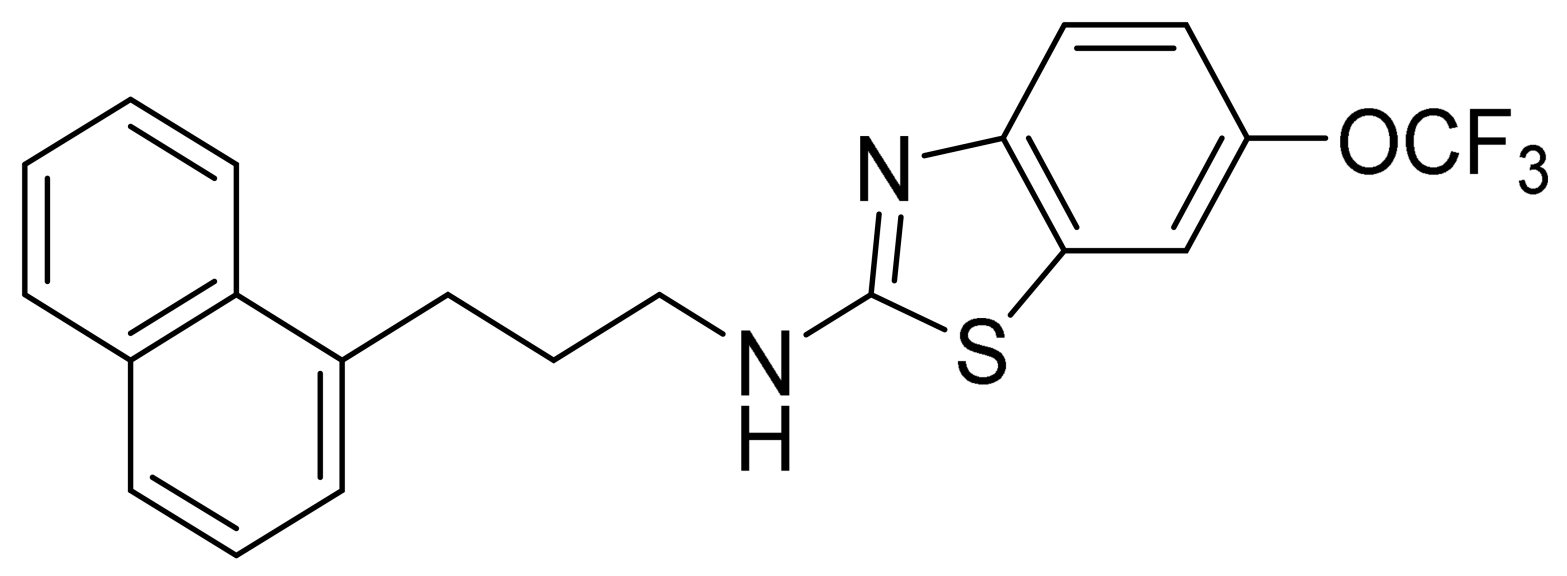	0	

**Table 3:** In vivo anti-inflammatory activity of selected benzothiazole derivatives.

Compound	Structure	% Reduction of Rat-Paw Edema ^a^
**5b**	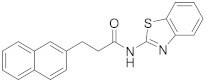	48.4 ** ± 2.16
**19a**	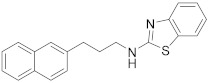	47.3 * ± 3.06
**19c**	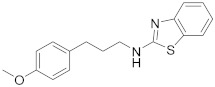	41.4 * ± 2.16
Indomethacin	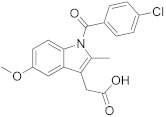	37.3 * ± 1.3

^a^ At a concentration of 0.01 mmol/kg. * *p <* 0.01 and ** *p <* 0.05, Student’s *t*-test.

## Data Availability

The data presented in this study are available on request from the corresponding author.

## Supplementary Materials

The following are available online at https://www.mdpi.com/article/10.3390/biom12020267/s1. Table S1. Names and code numbers of tested compounds; Figure S1. Effect of the inhibitor **19a** on IL-1/Fk-stimulated nitrite formation in rat mesangial cells; ^1^H NMR and ^13^C NMR spectra of the compounds synthesized, and HPLC traces of **5b**, **11**, **19a**, **19b**, **19c**, **19d**, **19e**, **23a** and **23c**. (PDF).

## Abbreviations

ACN, acetonitrile; 4-DMAP, 4-(dimethylamino)pyridine; AA, arachidonic acid; AD, Alzheimer’s disease; ATP, adenosine triphosphate; BSA, bovine serum albumin; COX-1, cyclooxygenase-1; COX-2, cyclooxygenase-2; DMEM, Dulbecco’s modified Eagle’s medium; DMF, *N,N*-dimethylformamide; DMSO, dimethyl sulfoxide; DTT, dithiothreitol; EC_50_, half maximal effective concentrations; EDCI, 1-ethyl-3-(3-dimethylaminopropyl)carbodiimide; ESI, electrospray ionization; Fk, forskolin; GIVA cPLA_2_, group IVA cytosolic phospholipase A_2_; GVIA iPLA_2_, group VIA phospholipase A_2_; GV sPLA_2_, group V secreted phospholipase A_2_; HEPES, 4-(2-hydroxyethyl)-1-piperazineethanesulfonic acid; HOBt, 1-hydroxybenzotriazole; IL-1, interleukin 1β; LPC, lysophosphatidylcholine; mPGES-1, microsomal prostaglandin E synthase-1; NSAIDs, non-steroidal anti-inflammatory drugs; PAPC, 1-palmitoyl-2-arachidonoyl-phosphatidylcholine; PGE_2_, prostaglandin E_2_; PGH_2_, prostaglandin H_2_; PI(4,5)P_2_, phosphatidylinositol 4,5-bisphosphate; PLA_2_s, phospholipases A_2_; TBAI, tetrabutylammonium iodide; TBHP, *tert*-butyl hydroperoxide; TEMPO, (2,2,6,6-τetramethylpiperidin-1-yl)oxyl; THF, tetrahydrofuran; Tris-HCl, tris(hydroxymethyl)aminomethane hydrochloride.
