# Supplementary material for: N-Acylated and N-Alkylated 2-Aminobenzothiazoles Are Novel Agents That Suppress the Generation of Prostaglandin E2
Source: Biomolecules. 2022 Feb 7;12(2):267. doi: 10.3390/biom12020267 (PMC8961538; doi:10.3390/biom12020267)
Supplement: Supplementary file 1 [file biomolecules-12-00267-s001.zip › biomolecules-1531702-supplementary.pdf]

N-Acylated and *N*-Alkylated 2-Aminobenzothiazoles are Novel Agents that Suppress the Generation of Prostaglandin E_2

Maria A. Theodoropoulou,[1,2,#] Anastasia Psarra,[1,2,#] Martin Erhardt,[3] Aikaterini Nikolaou,[1,2] Anna-Dimitra D. Gerogiannopoulou,[1,2] Dimitra Hadjipavlou-Litina,[4] Daiki Hayashi,[5] Edward A. Dennis,[5] Andrea Huwiler,[3] George Kokotos[1,2,*]

[1]*Laboratory of Organic Chemistry, Department of Chemistry, National and Kapodistrian University of Athens, Panepistimiopolis, Athens 15771, Greece*

[2]*Center of Excellence for Drug Design and Discovery, National and Kapodistrian University of Athens, Athens 15771, Greece*

[3]*Institute of Pharmacology, University of Bern, CH-3010 Bern, Switzerland*

[4]*Department of Pharmaceutical Chemistry, School of Pharmacy, Aristotelian University of Thessaloniki, 54124 Thessaloniki, Greece*

[5]*Department of Chemistry and Biochemistry and Department of Pharmacology, School of Medicine, University of California, San Diego, La Jolla, California 92093-0601, USA*

[#]*These authors contributed equally.*

*To whom correspondence should be addressed. For G.K.: phone: +30 210 7274462; fax: +30 210 7274761; E-mail: gkokotos@chem.uoa.gr.

SUPPLEMENTARY MATERIALS

Contents

	Page
**Table S1. Names and code numbers of tested compounds.**	**2**
**Figure S1. Effect of the inhibitor 19a on IL-1/Fk-stimulated nitrite formation in rat mesangial cells.**	**4**
**^{1}H NMR and ^{13}C NMR spectra of the compounds synthesized.**	**5**
**HPLC traces of 5b, 11, 19a, 19b, 19c, 19d, 19e, 23a and 23c.**	**40**

Table S1. Names and code numbers of tested compounds.

Compound chemical name	No	Code number
N-(Benzo[*d*]thiazol-2-yl)-4-(naphthalen-2-yl)butanamide	**5a**	GK509
N-(Benzo[*d*]thiazol-2-yl)-3-(naphthalen-2-yl)propanamide	**5b**	GK510
N-(Benzo[*d*]oxazol-2-yl)-3-(naphthalen-2-yl)propanamide	**5c**	GK538
N-(Benzo[*d*]oxazol-2-yl)-4-(naphthalen-2-yl)butanamide	**5d**	GK540
N-(1*H*-Benzo[*d*]imidazol-2-yl)-3-(naphthalen-2-yl)propanamide	**5e**	GK539
N-(1*H*-Benzo[*d*]imidazol-2-yl)-4-(naphthalen-2-yl)butanamide	**5f**	GK541
N-(Benzo[*d*]thiazol-2-yl)-2-(naphthalen-2-yloxy)acetamide	**7**	GK591
(*E*)-*N*-(Benzo[*d*]thiazol-2-yl)-3-(naphthalen-2-yl)acrylamide	**9a**	GK522
(*E*)-*N*-(Benzo[*d*]thiazol-2-yl)-3-(4-methoxyphenyl)acrylamide	**9b**	GK523
N-(Benzo[*d*]thiazol-2-yl)-3-(4-methoxyphenyl)propanamide	**11**	GK521
N-(3-(Naphthalen-2-yl)propyl)benzo[*d*]thiazole-2-carboxamide	**14**	GK508
4-Methoxybenzyl benzo[*d*]thiazol-2-ylcarbamate	**16a**	GK524
Naphthalen-2-ylmethyl benzo[*d*]thiazol-2-ylcarbamate	**16b**	GK525
N-(1-Methoxy-3-(naphthalen-2-yl)propyl)benzo[*d*]thiazol-2-amine	**18a**	GK542
N-(1-Methoxy-3-phenylpropyl)benzo[*d*]thiazol-2-amine	**18b**	GK561
N-(1-Methoxy-3-(4-methoxyphenyl)propyl)benzo[*d*]thiazol-2-amine	**18c**	GK559
N-(3-(Naphthalen-2-yl)propyl)benzo[*d*]thiazol-2-amine	**19a**	GK543
N-(3-Phenylpropyl)benzo[*d*]thiazol-2-amine	**19b**	GK562
N-(3-(4-Methoxyphenyl)propyl)benzo[*d*]thiazol-2-amine	**19c**	GK560
N-(3-(Naphthalen-1-yl)propyl)benzo[*d*]thiazol-2-amine	**19d**	GK593
N-(3-(Naphthalen-1-yl)propyl)thiazol-2-amine	**19e**	GK610
N-(3-Phenylpropyl)thiazol-2-amine	**19f**	GK643
N-(3-(Naphthalen-2-yl)propyl)-6-(trifluoromethoxy)benzo[*d*]thiazol-2-amine	**19g**	GK642
N-(3-(Naphthalen-1-yl)propyl)-6-(trifluoromethoxy)benzo[*d*]thiazol-2-amine	**19h**	GK644

N-(3-(Naphthalen-2-yl)propyl)benzo[*d*]oxazol-2-amine	**23a**	GK566
N-(3-(4-Methoxyphenyl)propyl)benzo[*d*]oxazol-2-amine	**23b**	GK567
N-(2-(Naphthalen-2-yloxy)ethyl)benzo[*d*]oxazol-2-amine	**23c**	GK592
N-(2-(Naphthalen-1-yloxy)ethyl)benzo[*d*]oxazol-2-amine	**23d**	GK604
N-(2-(Naphthalen-2-yloxy)ethyl)benzo[*d*]thiazol-2-amine	**25a**	GK606
N-(2-(Naphthalen-1-yloxy)ethyl)benzo[*d*]thiazol-2-amine	**25b**	GK607

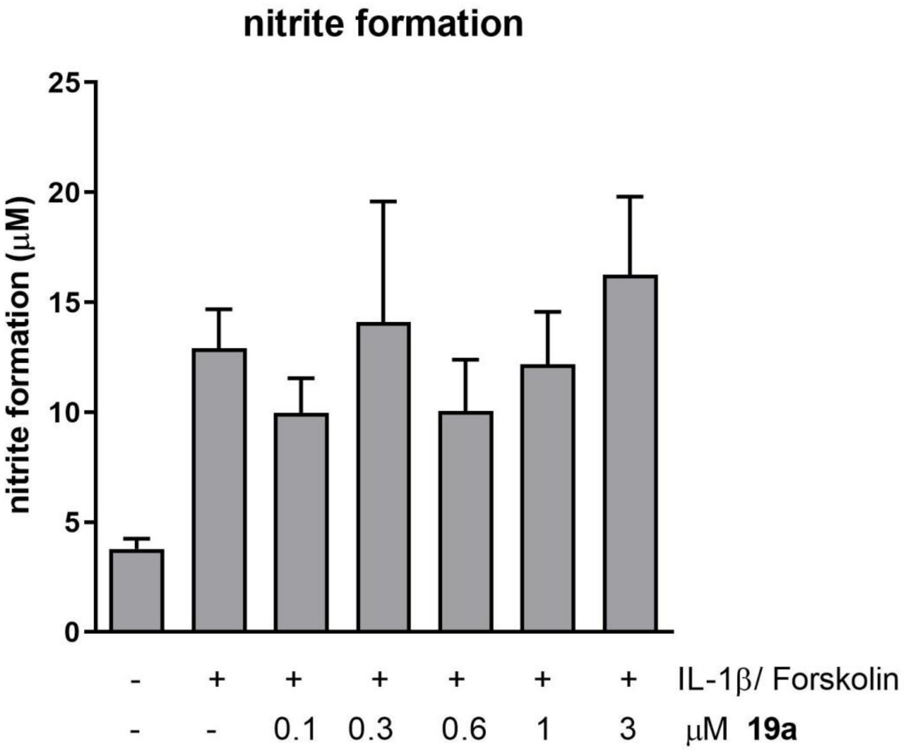

Figure S1. Effect of the inhibitor **19a** on IL-1/Fk-stimulated nitrite formation in rat mesangial cells.

Cells were pretreated for 20 min with the indicated concentrations of **19a** and then stimulated for 24 h in the absence (-) or presence (+) of 1 nM interleukin 1β (IL-1) plus 5 μM forskolin (Fk). Nitrite was quantified in supernatants using Griess assay as previously described.[1] Data are presented as mM of nitrite and are means +/- S.D. (n = 3).

References

1. Mühl, H.; Kunz, D.; Rob, P.; Pfeilschifter, J. Cyclosporin derivatives inhibit interleukin 1β induction of nitric oxide synthase in renal mesangial cells. *Eur. J. Pharmacol.* **1993**, *249*, 95-100.

NMR Spectra

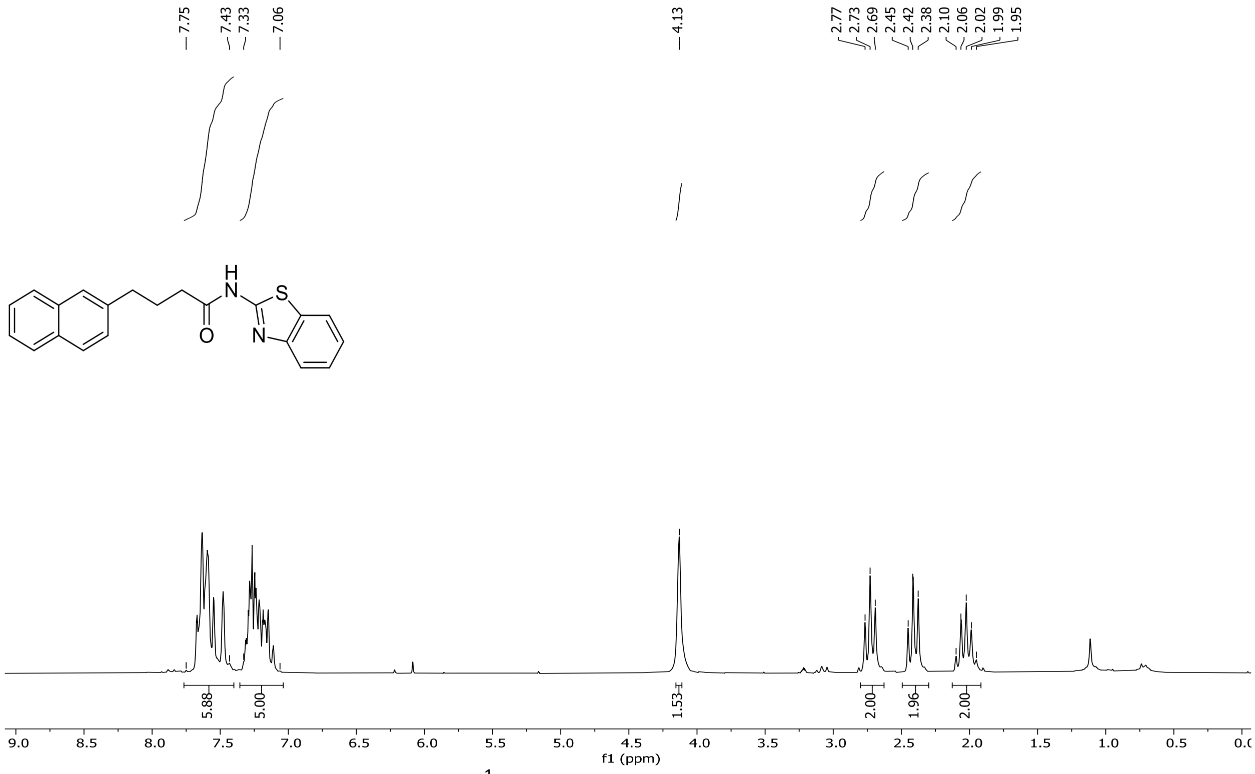

^{1}H NMR spectrum of **5a**.

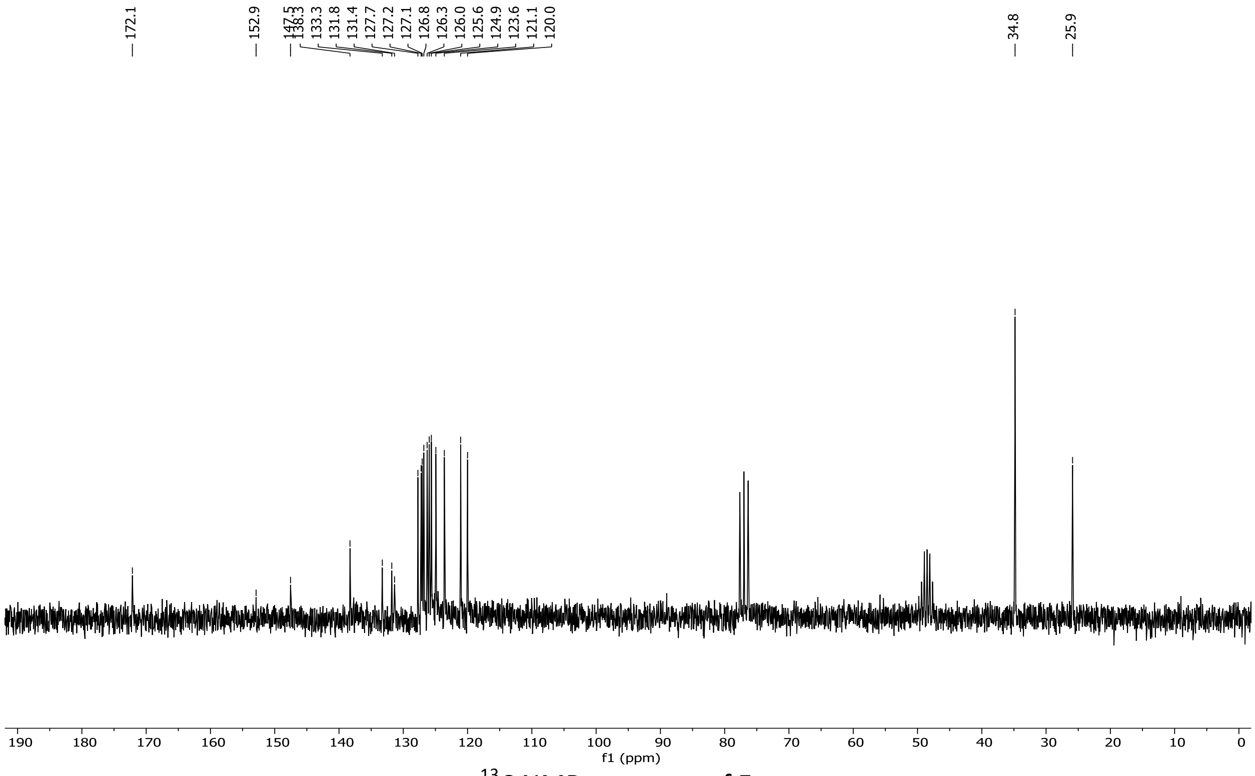

^{13}C NMR spectrum of **5a**.

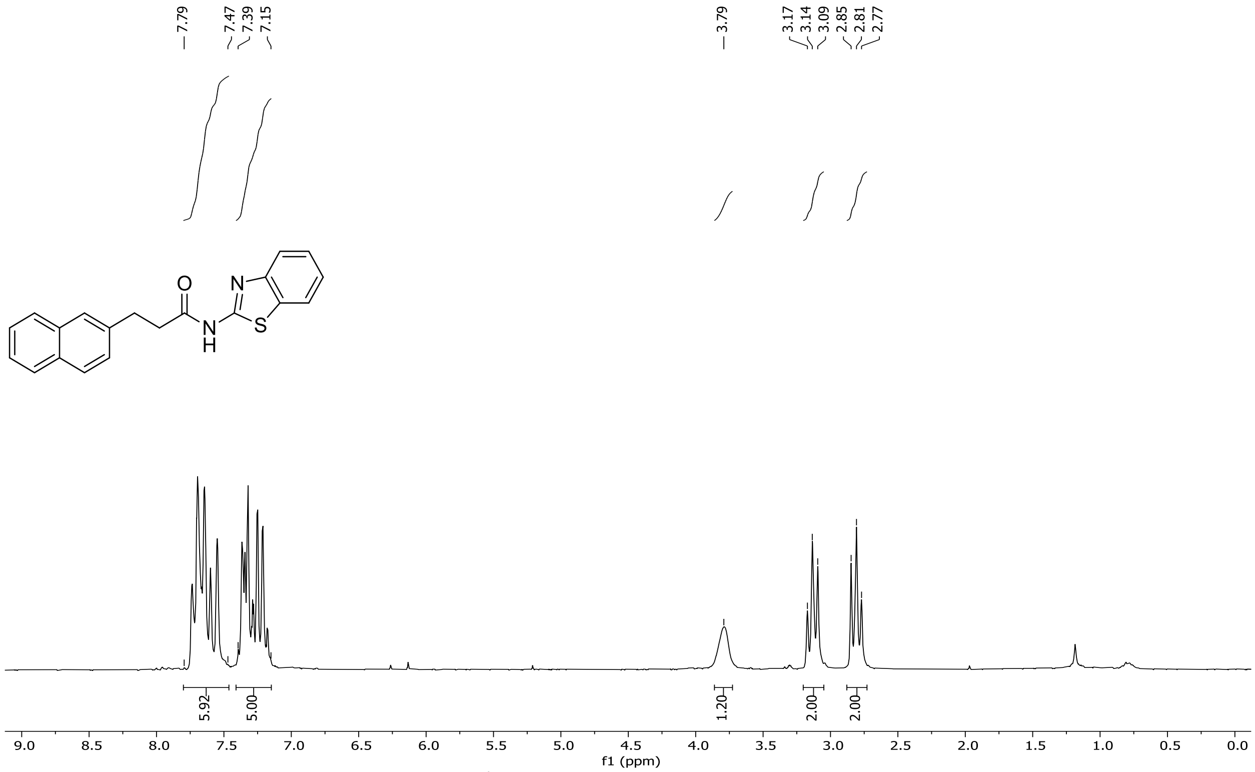

^{1}H NMR spectrum of **5b**.

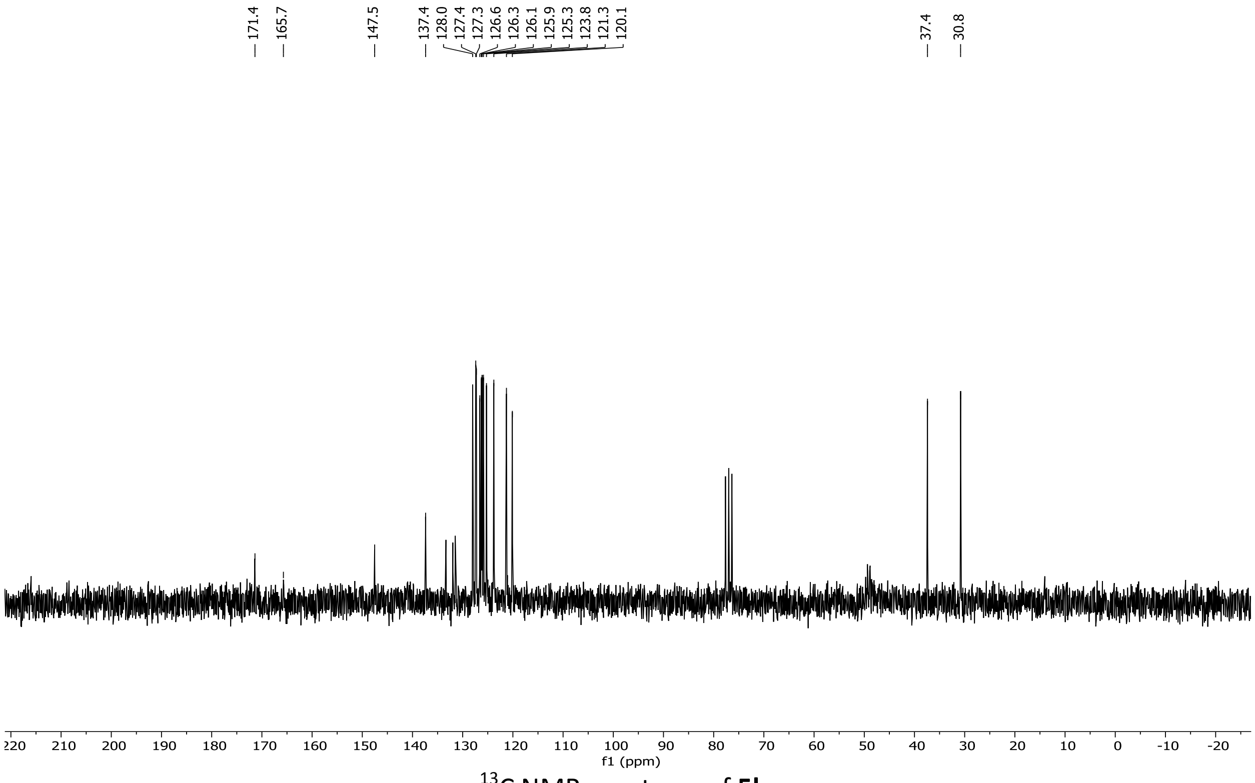

^{13}C NMR spectrum of **5b**.

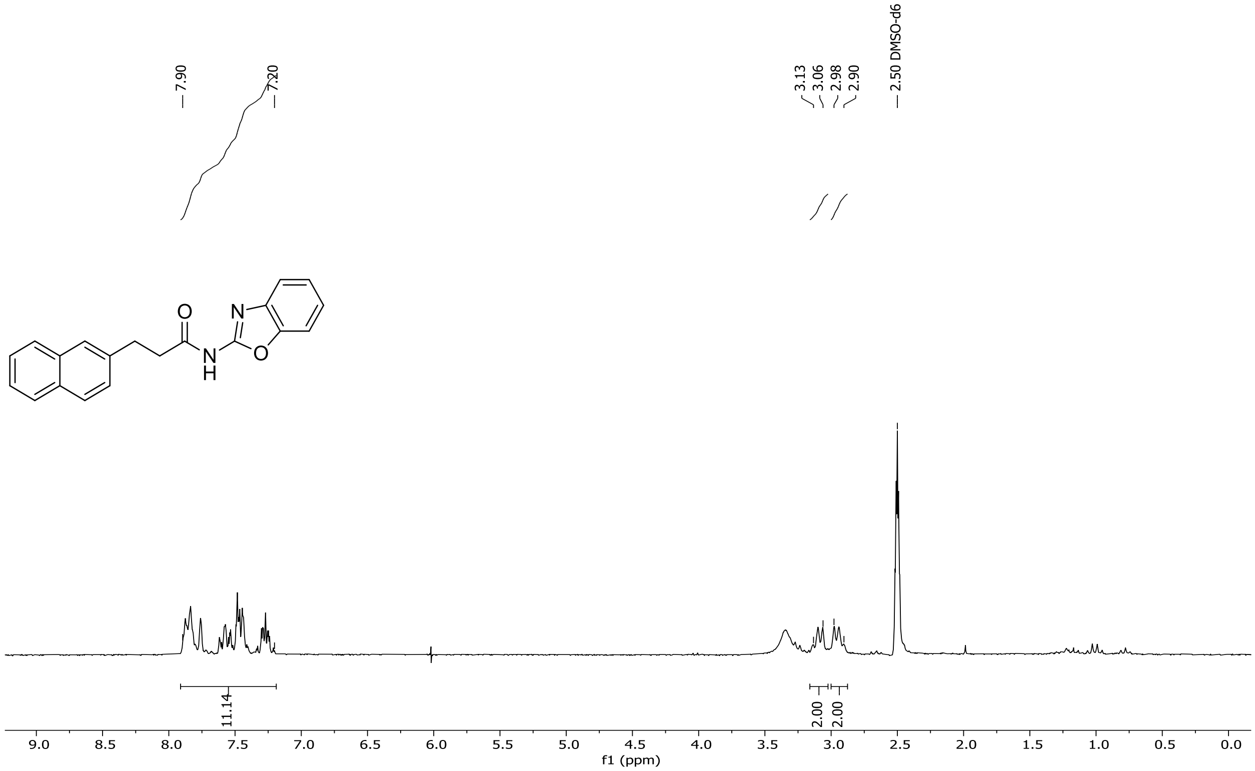

^{1}H NMR spectrum of **5c**.

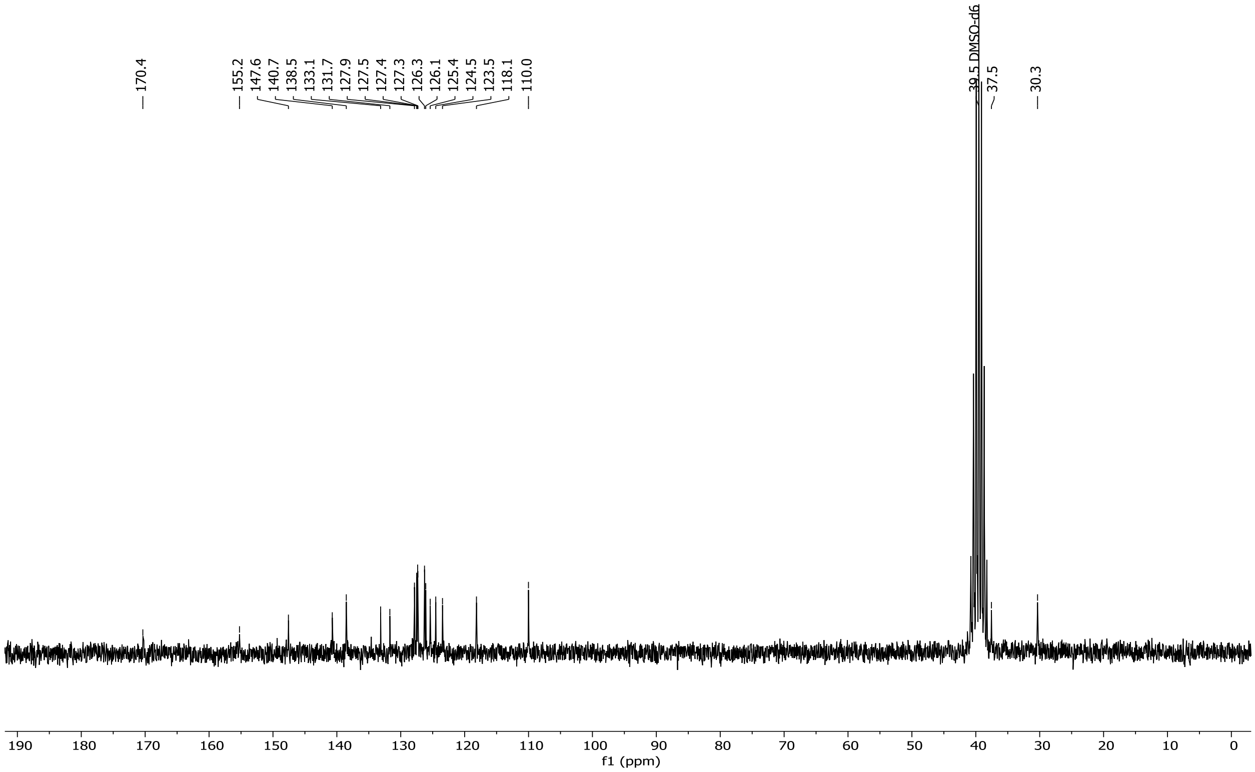

^{13}C NMR spectrum of **5c**.

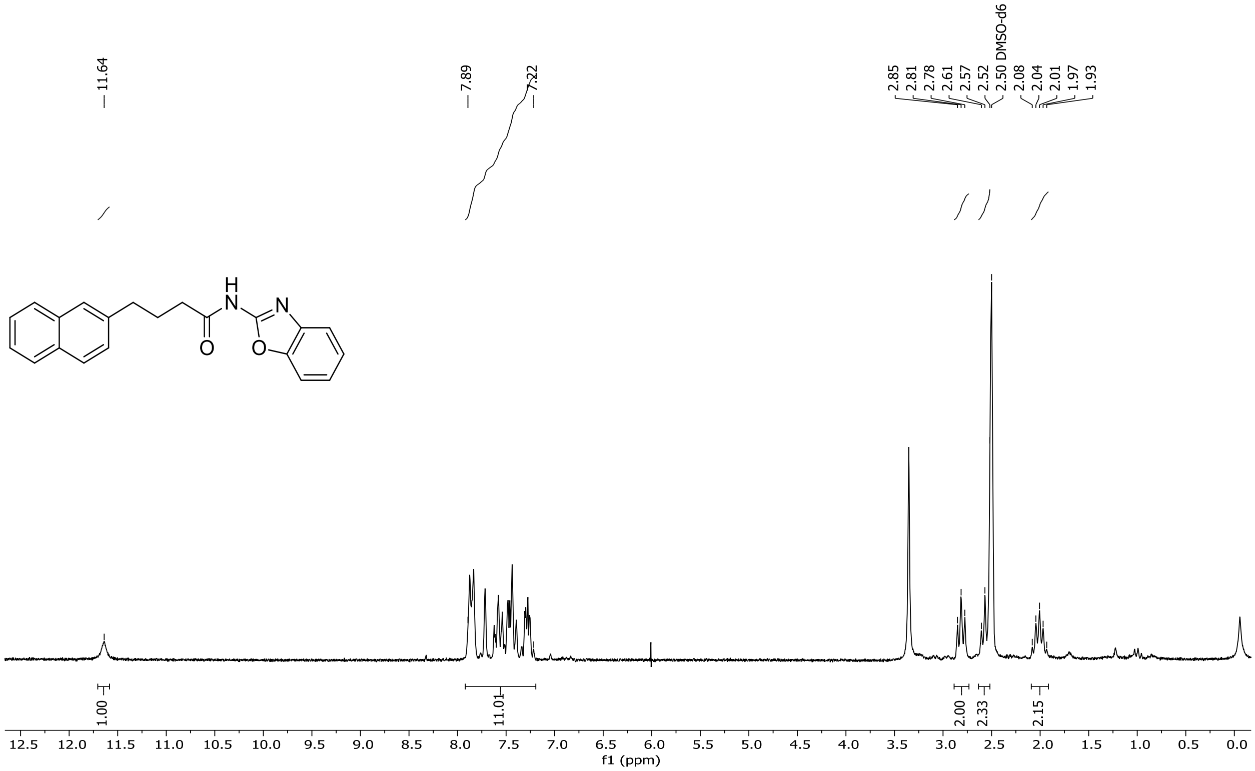

^{1}H NMR spectrum of **5d**.

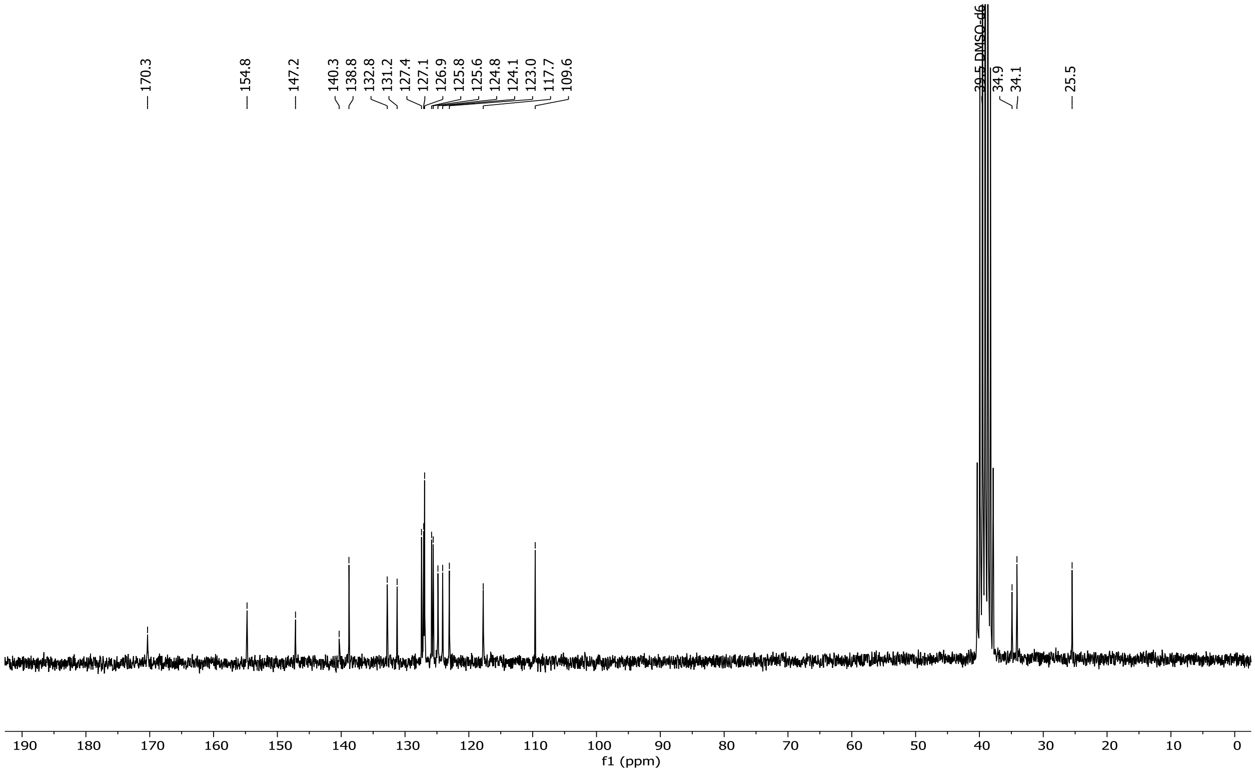

^{13}C NMR spectrum of **5d**.

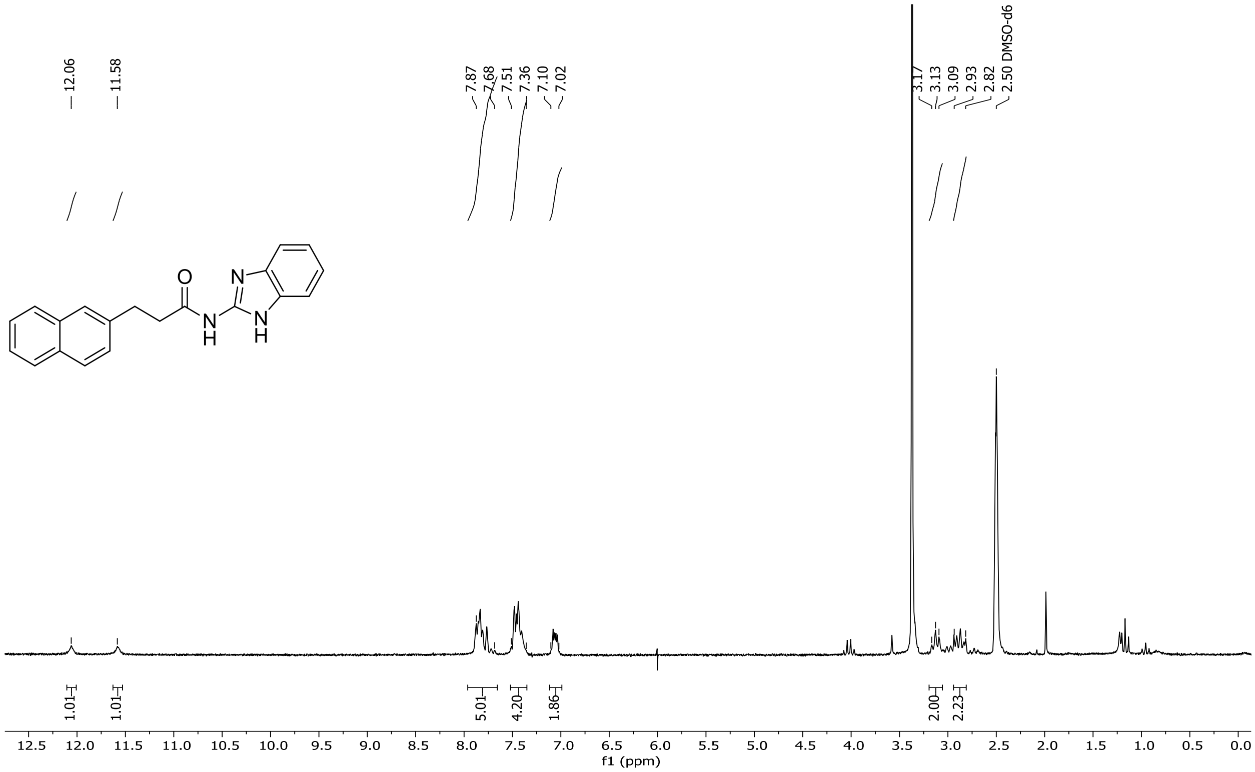

^{1}H NMR spectrum of **5e**.

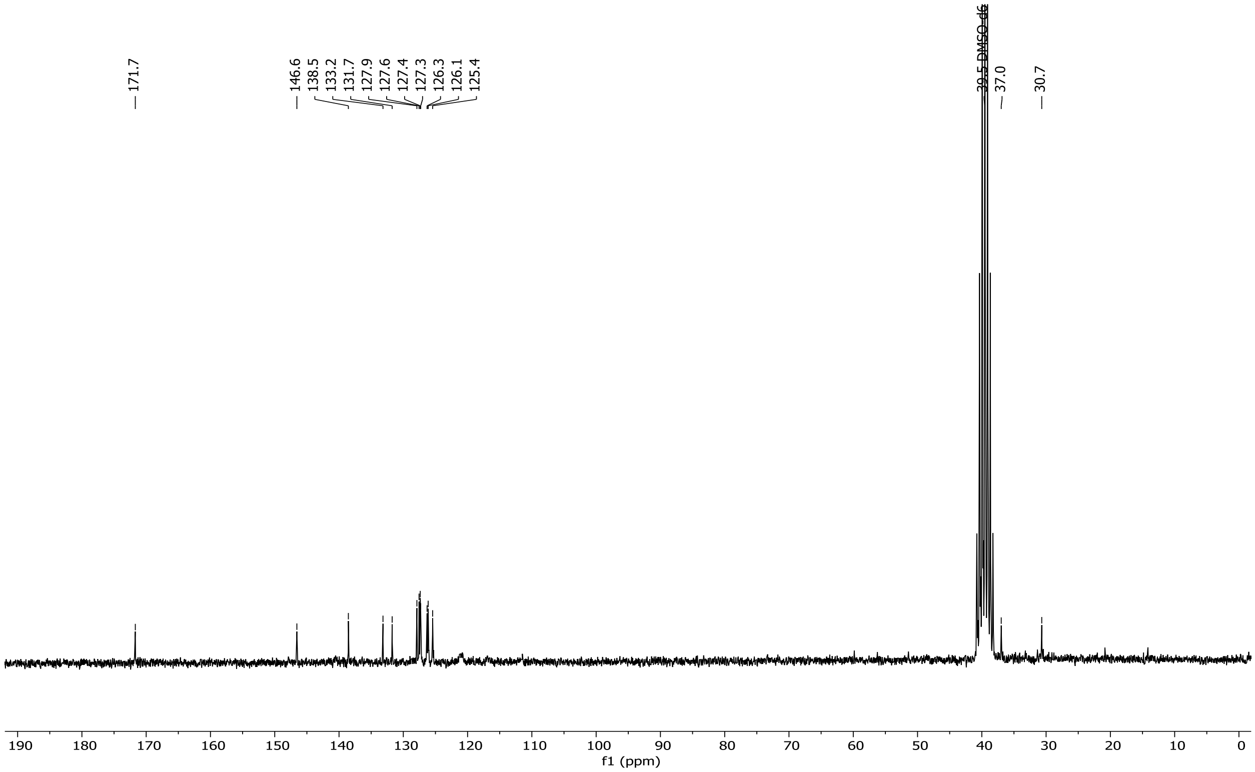

^{13}C NMR spectrum of **5e**.

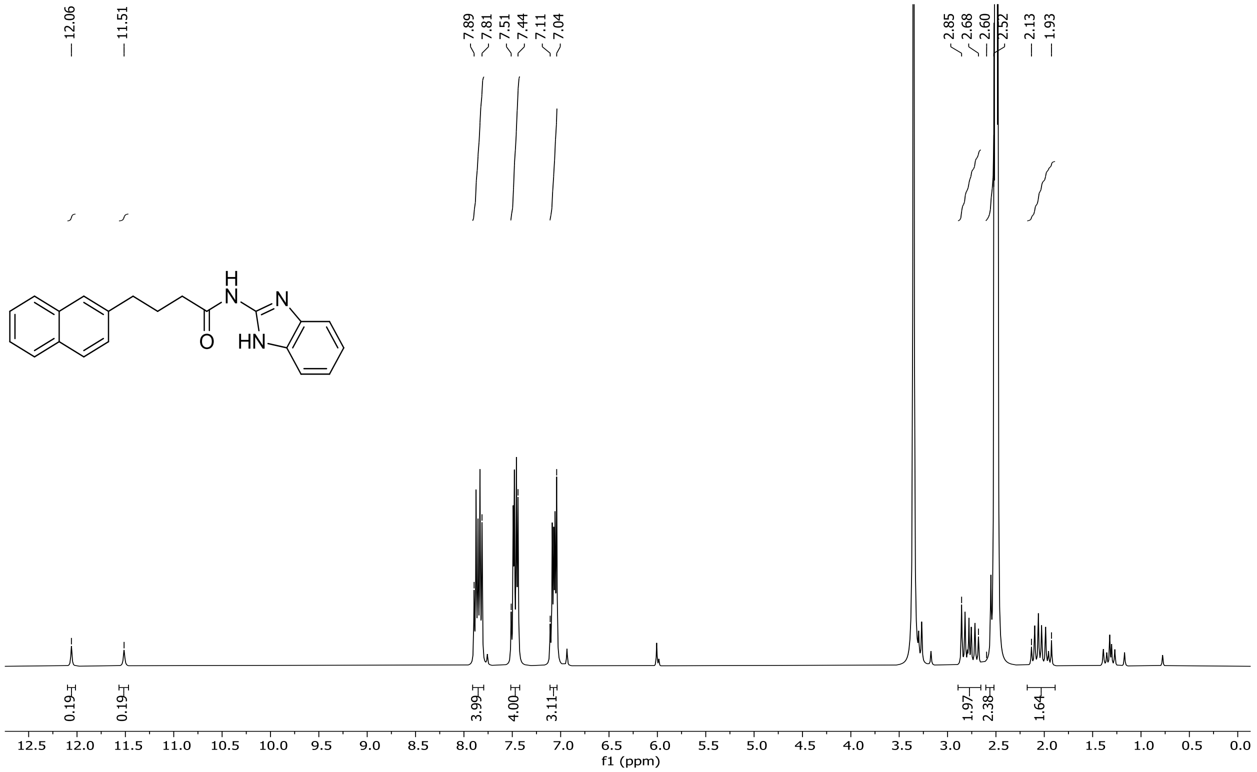

^{1}H NMR spectrum of **5f**.

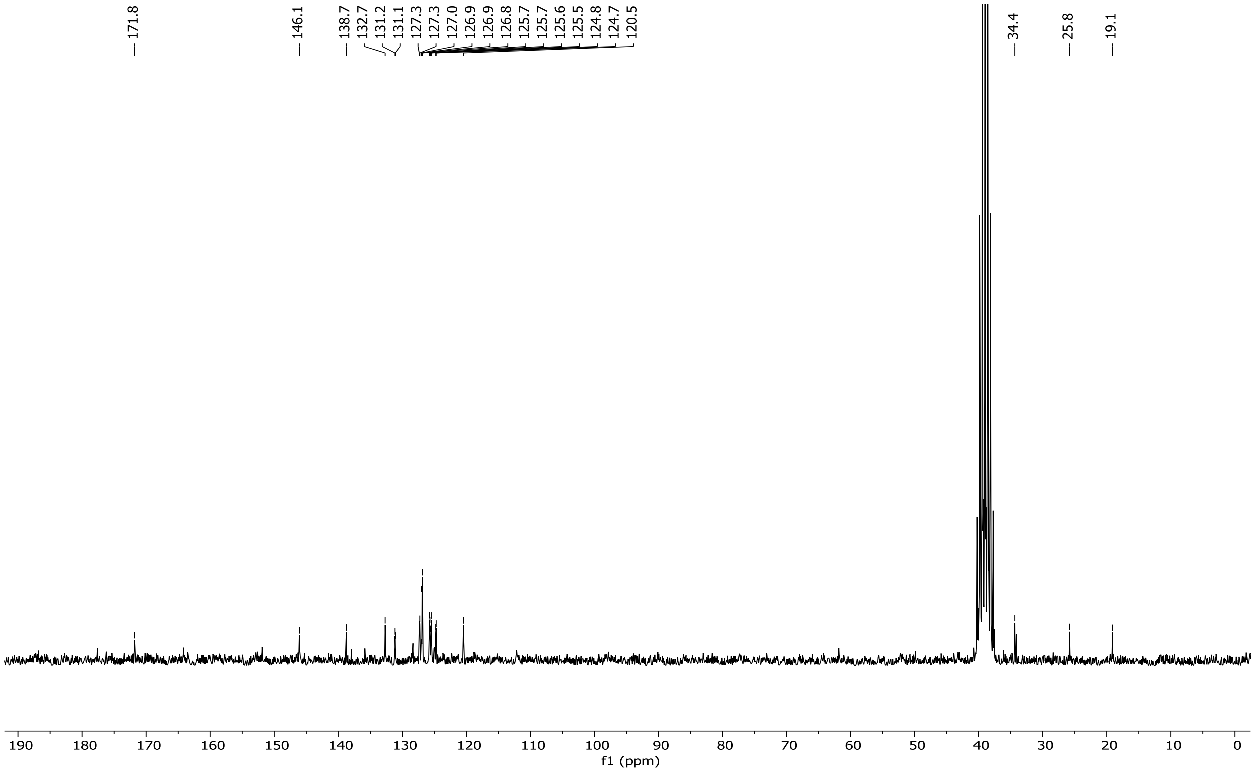

^{13}C NMR spectrum of **5f**.

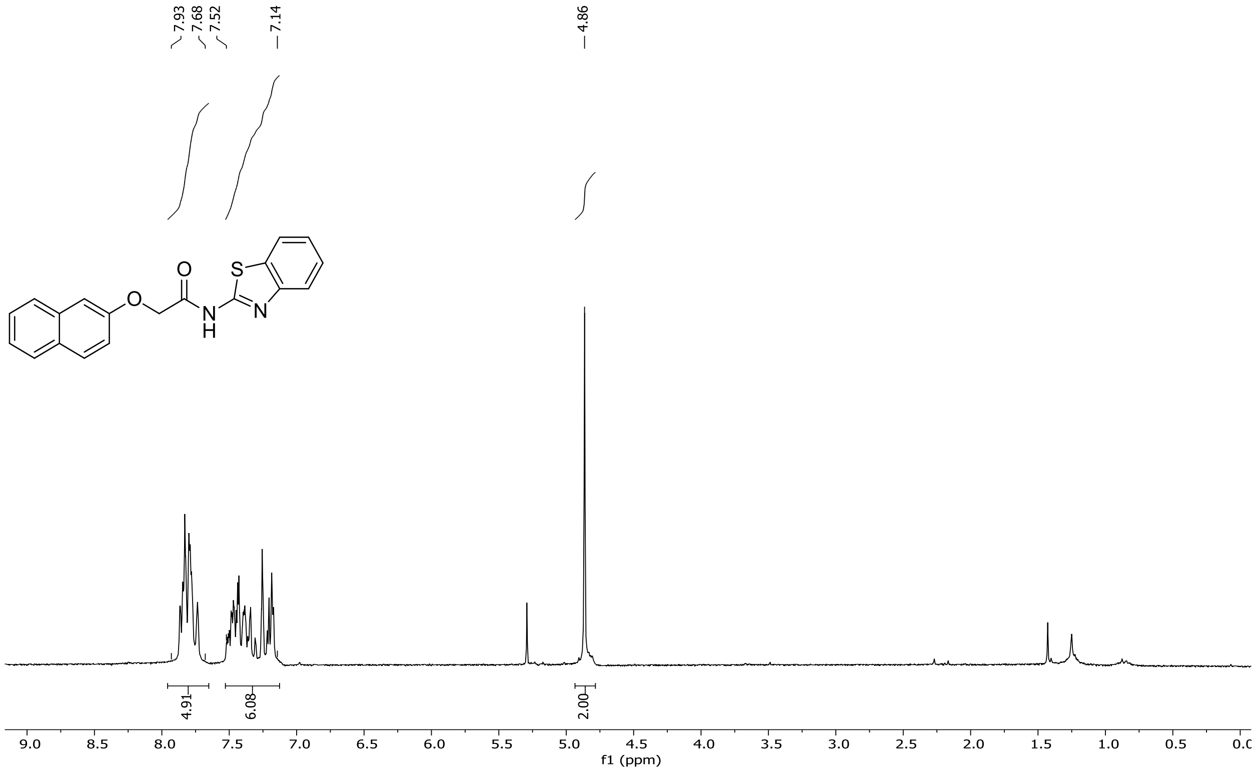

^{1}H NMR spectrum of **7**.

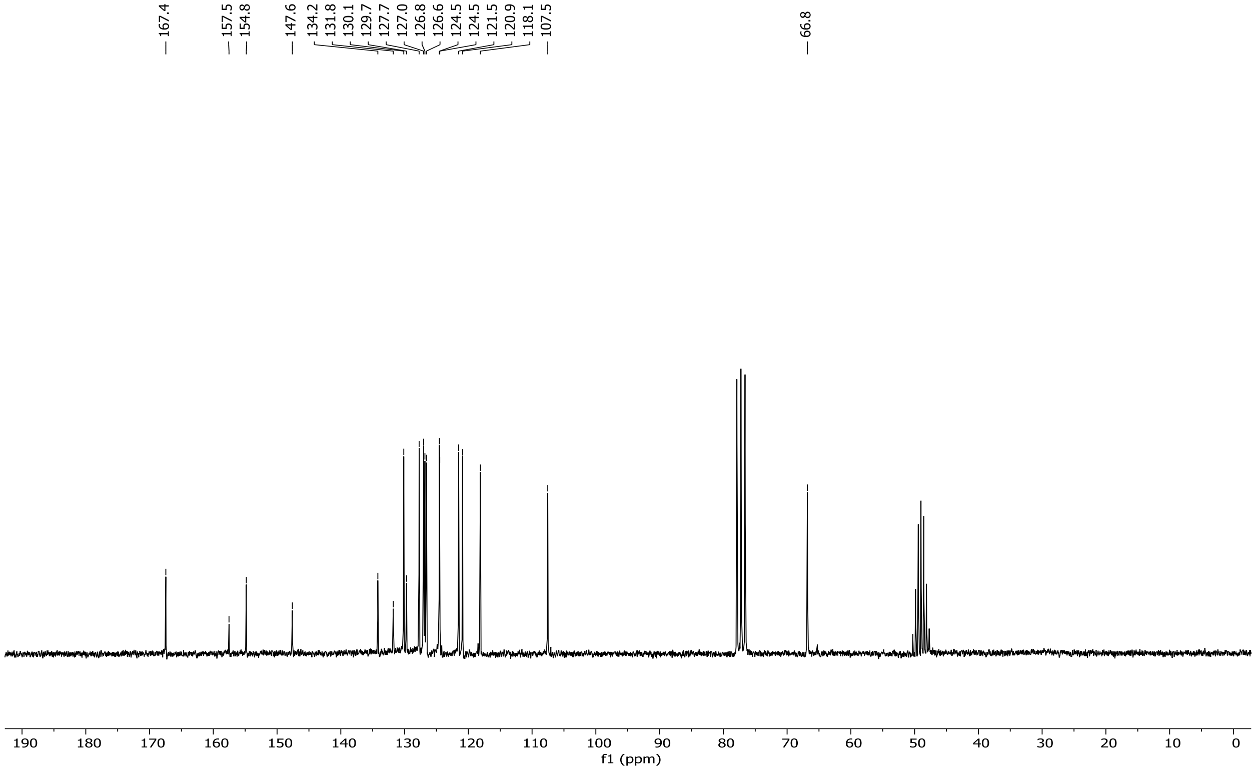

^{13}C NMR spectrum of **7**.

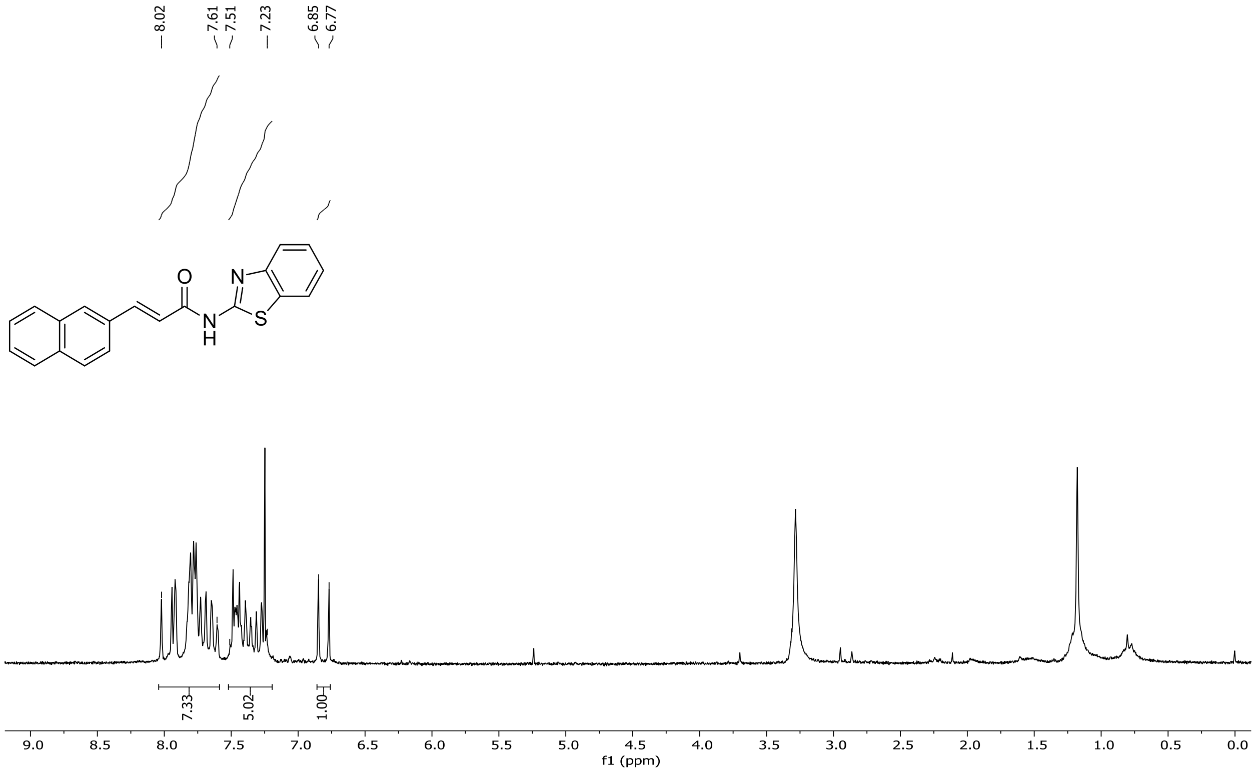

^{1}H NMR spectrum of **9a**.

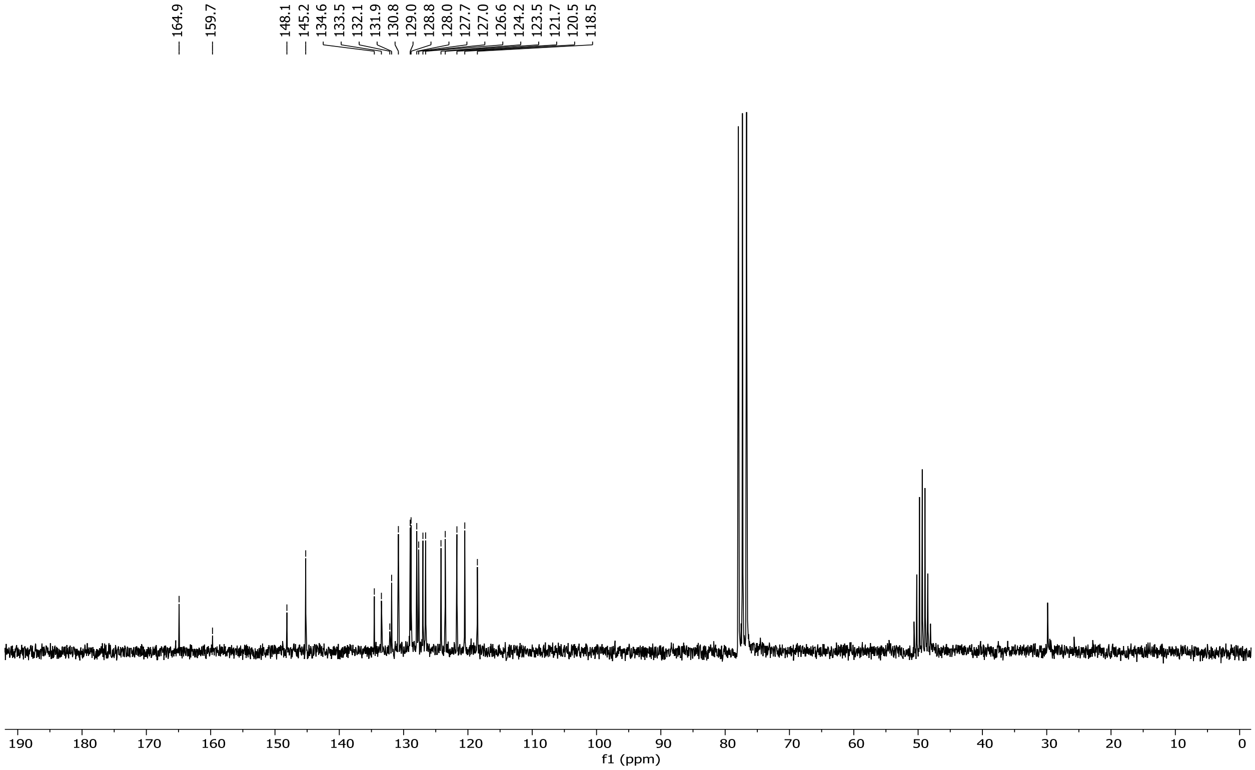

^{13}C NMR spectrum of **9a**.

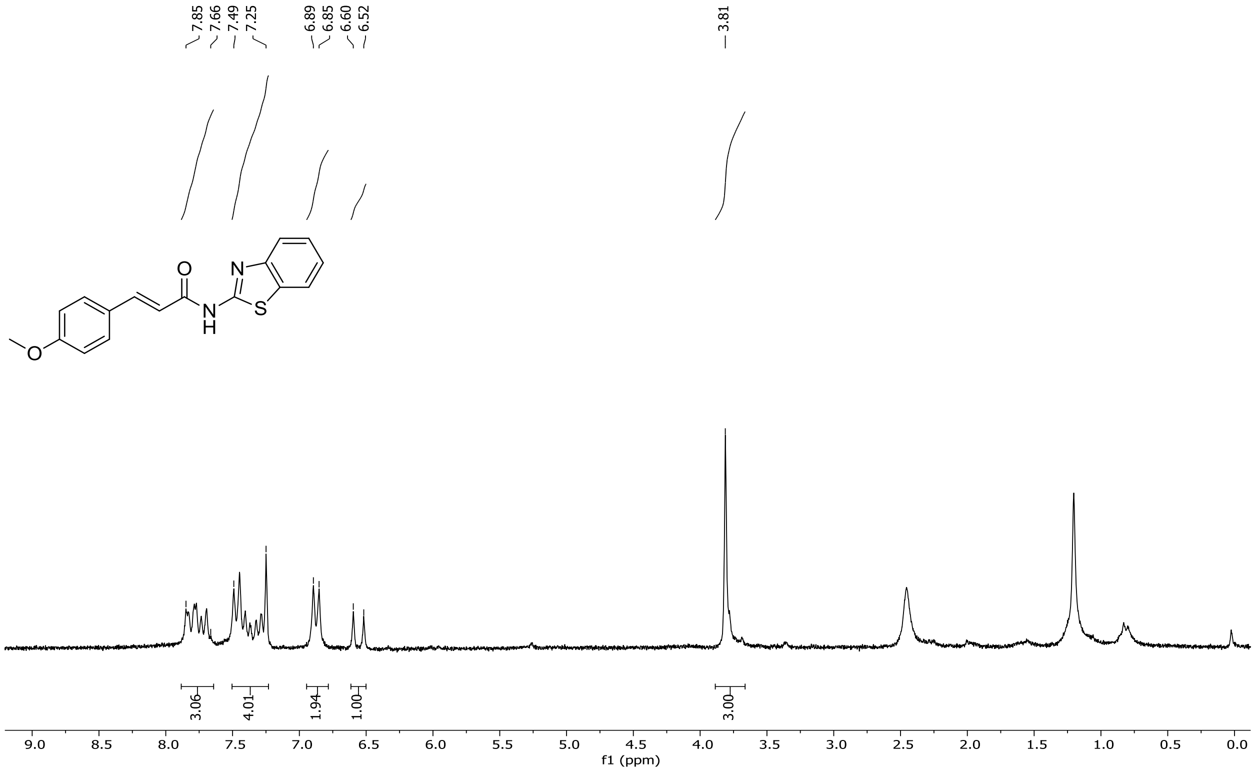

^{1}H NMR spectrum of **9b**.

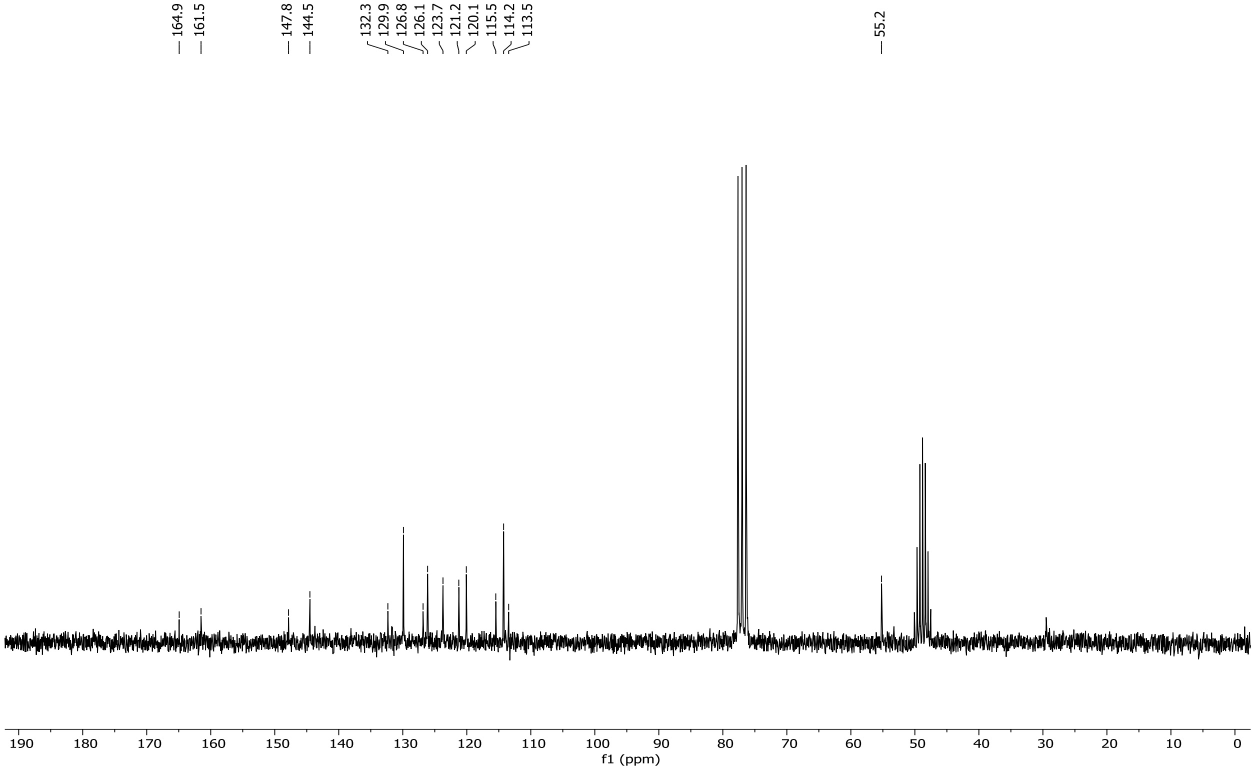

^{13}C NMR spectrum of **9b**.

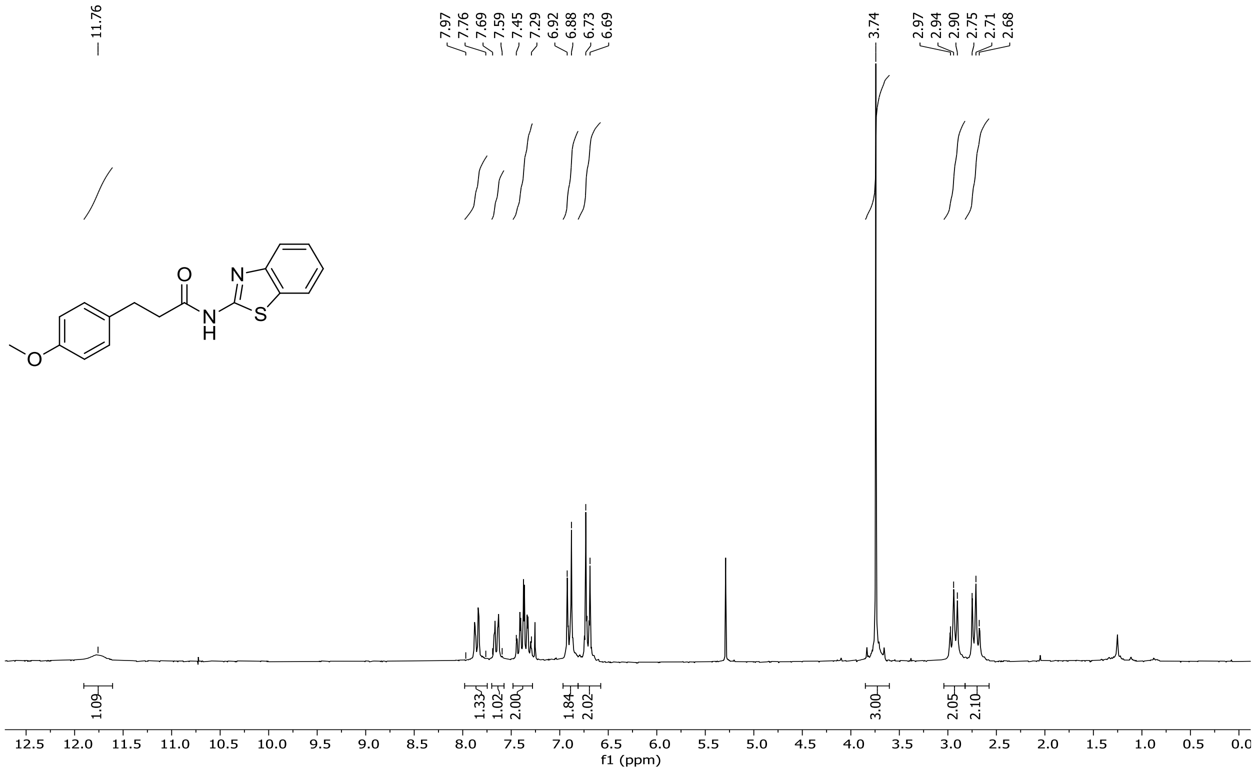

^{1}H NMR spectrum of **11**.

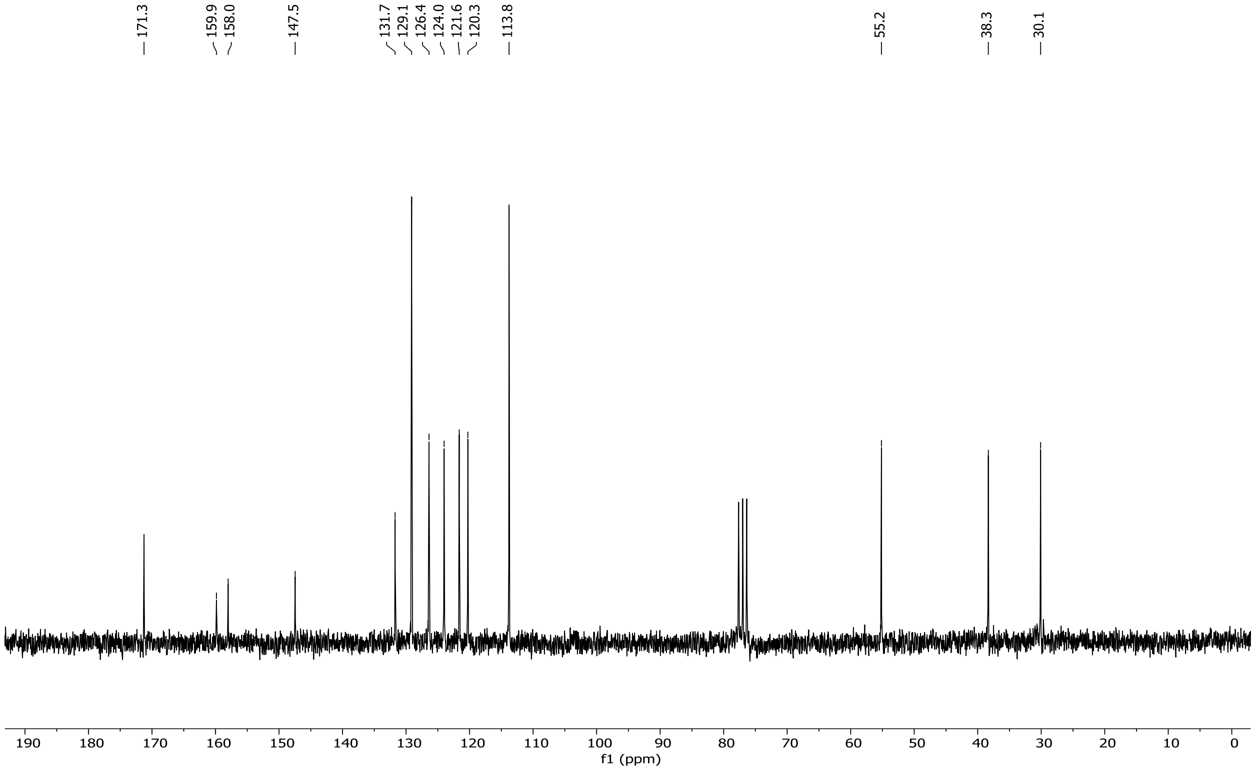

^{13}C NMR spectrum of **11**.

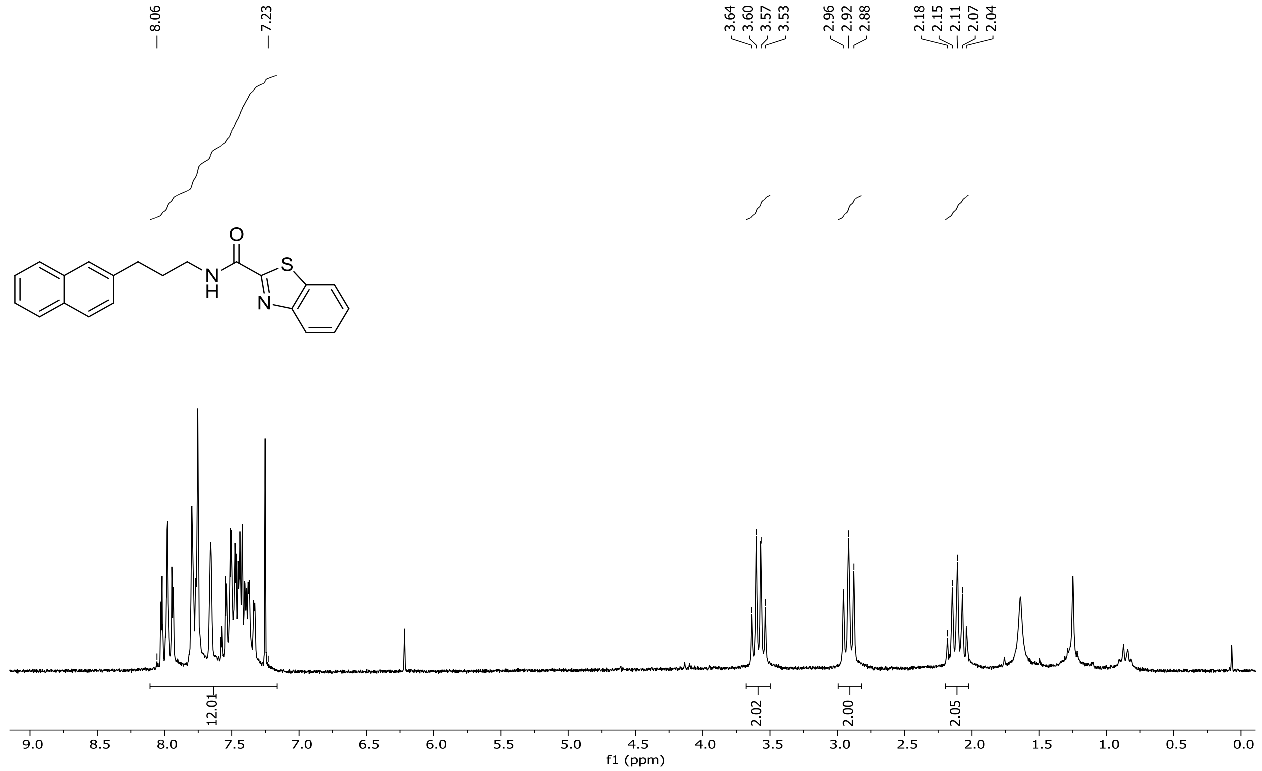

^{1}H NMR spectrum of **14**.

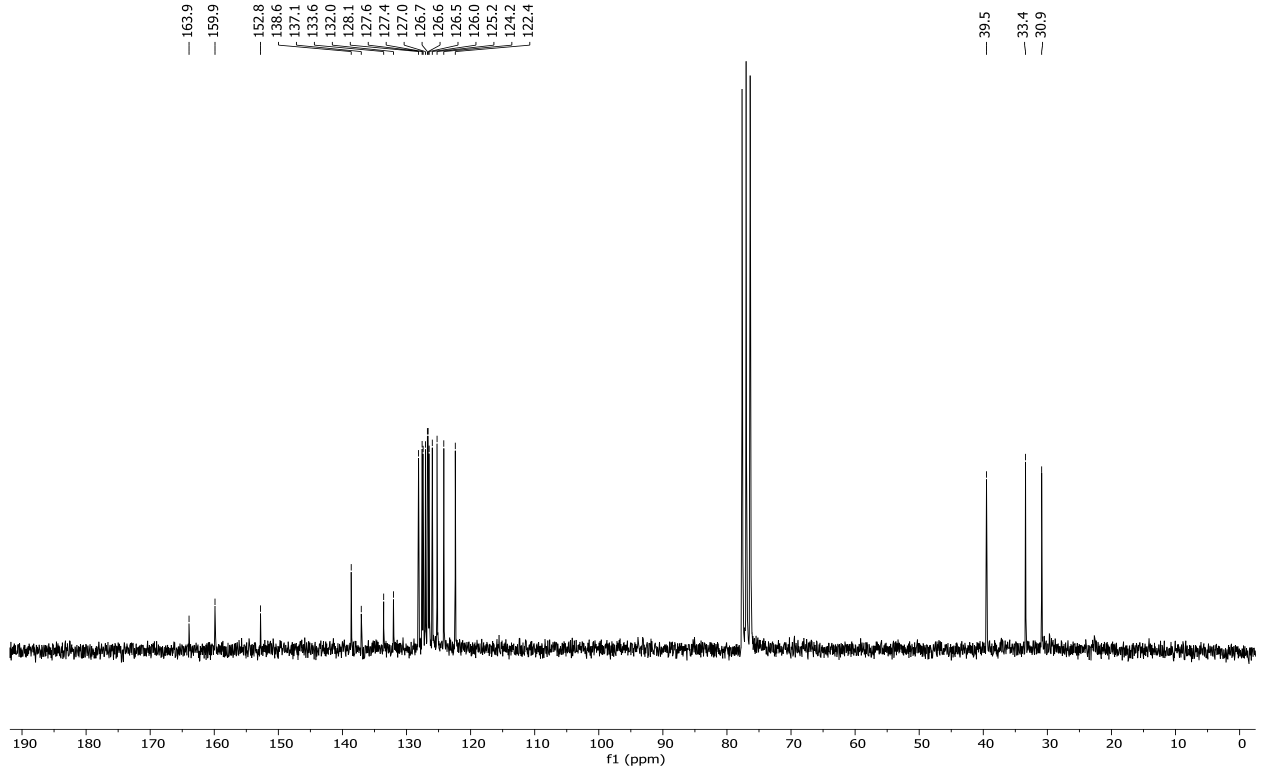

^{13}C NMR spectrum of **14**.

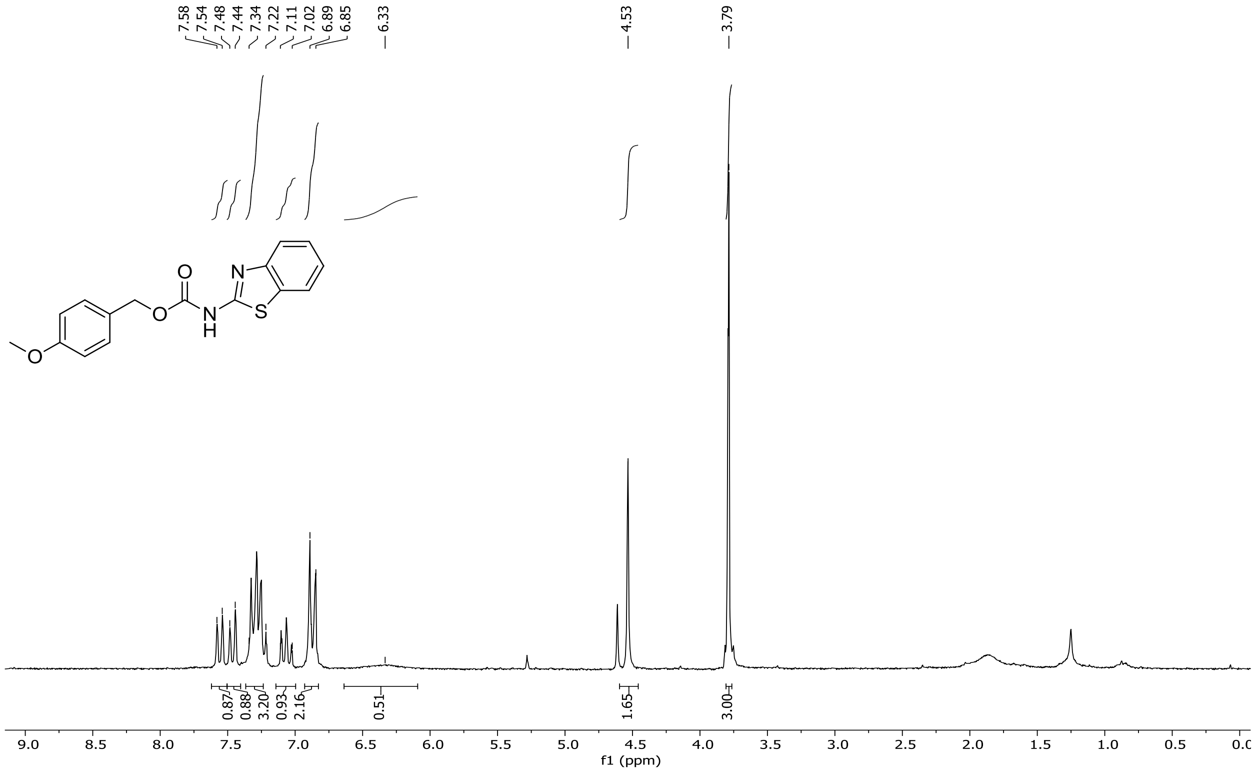

^{1}H NMR spectrum of **16a**.

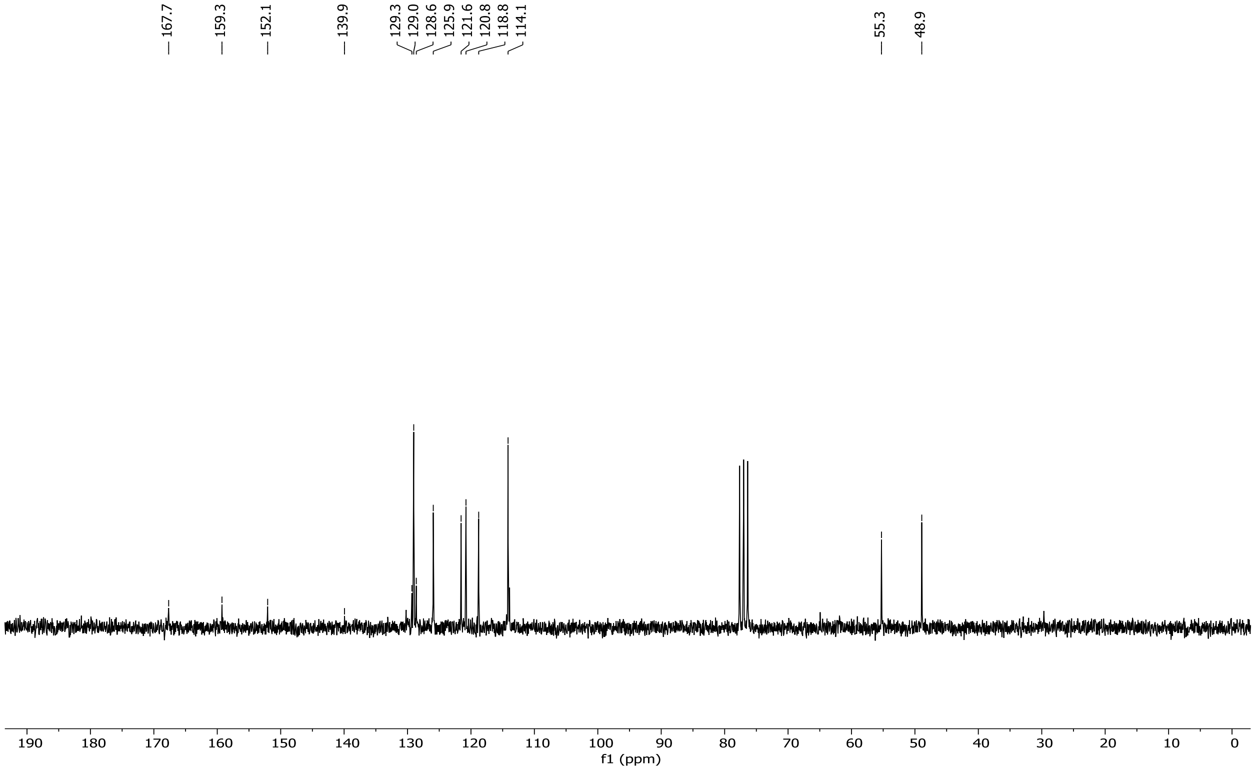

^{13}C NMR spectrum of **16a**.

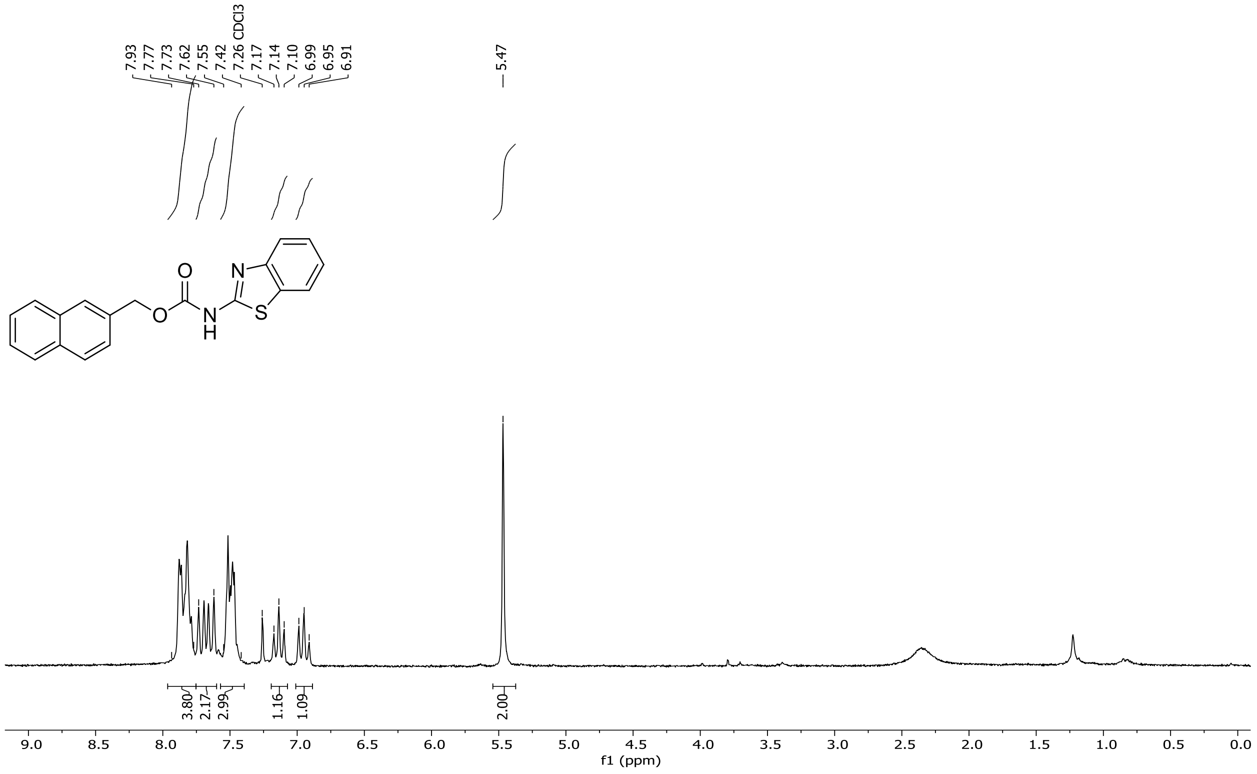

^{1}H NMR spectrum of **16b**.

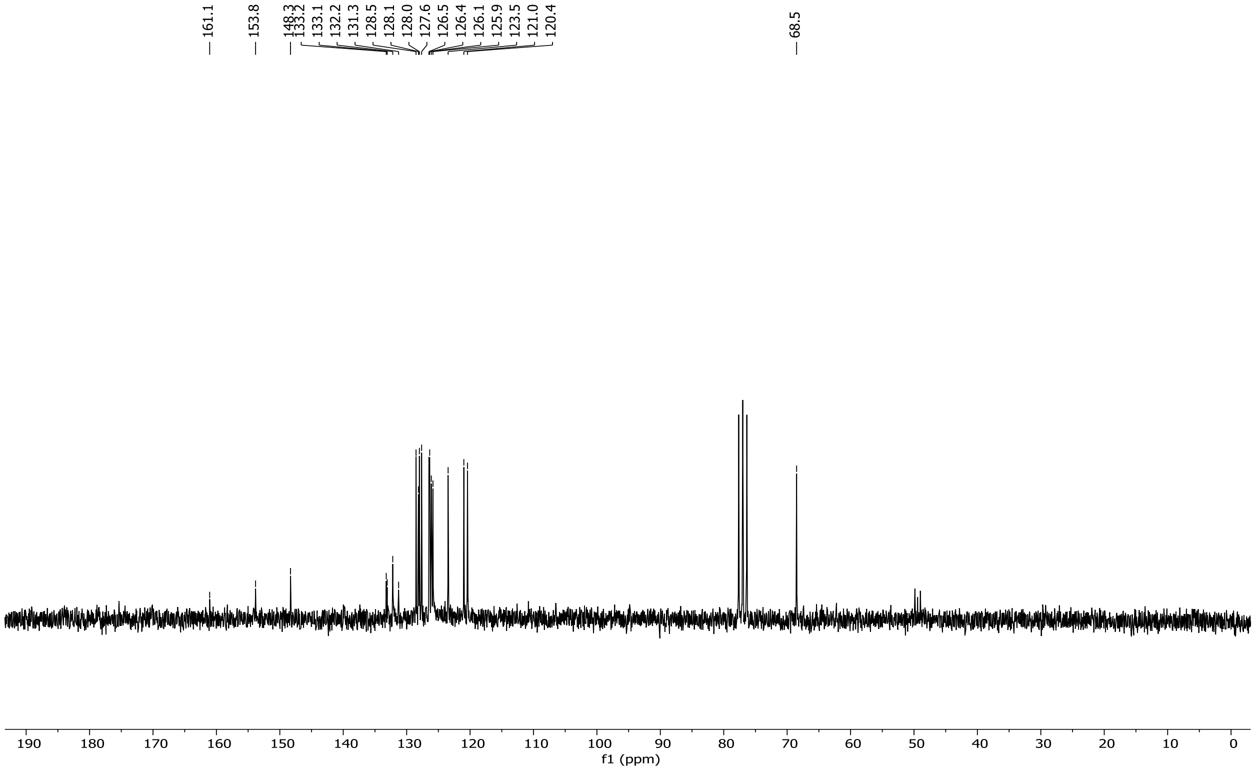

^{13}C NMR spectrum of **16b**.

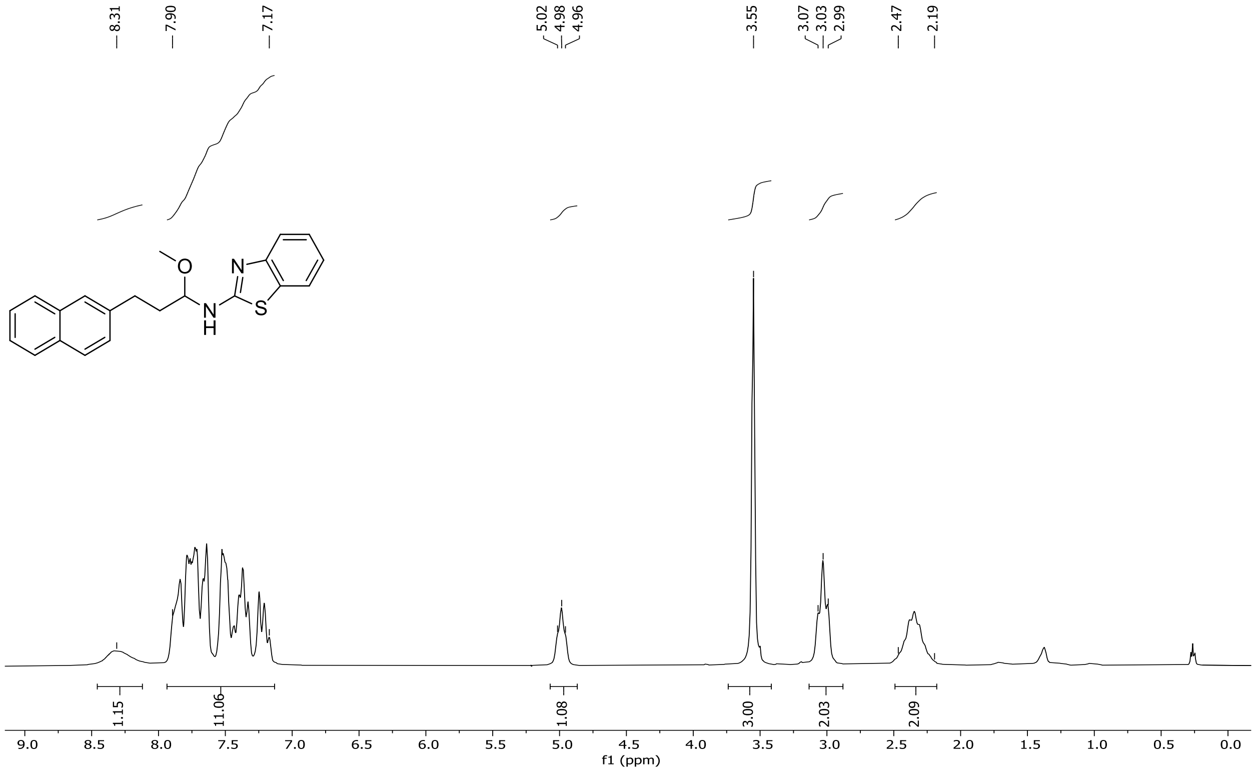

^{1}H NMR spectrum of **18a**.

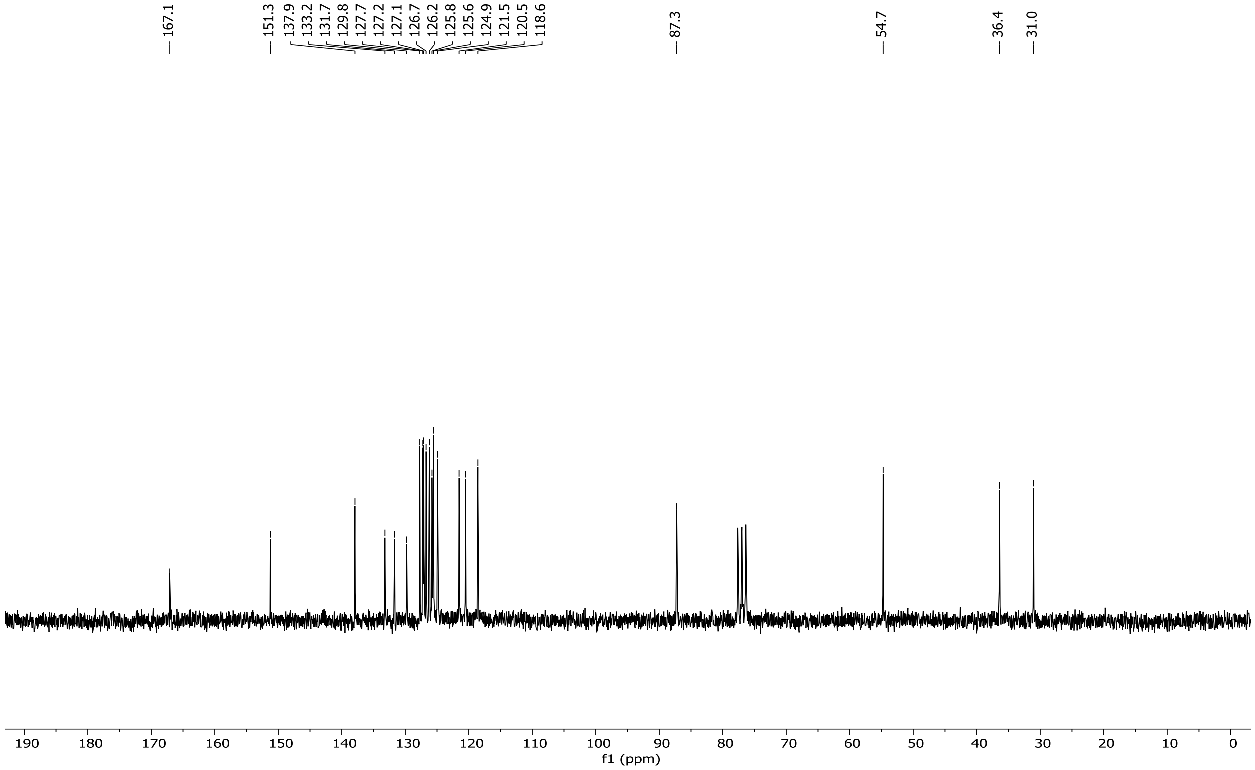

^{13}C NMR spectrum of **18a**.

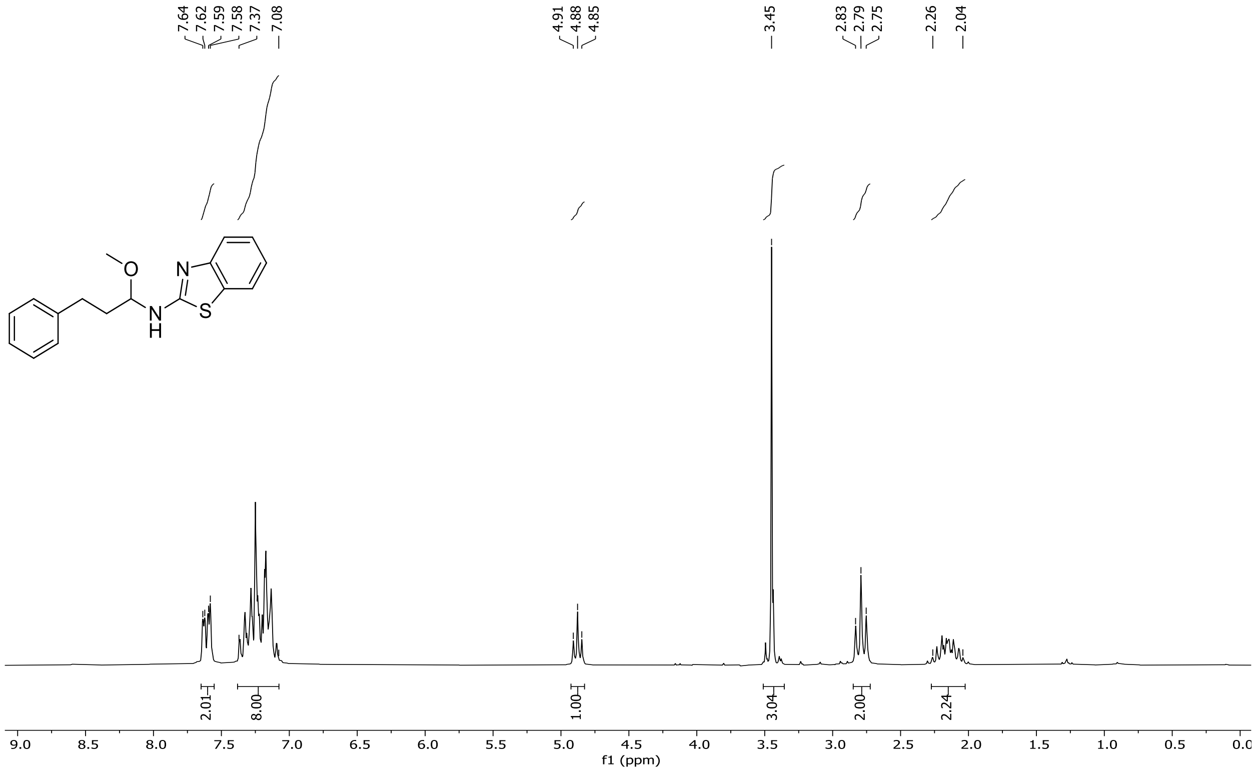

^{1}H NMR spectrum of **18b**.

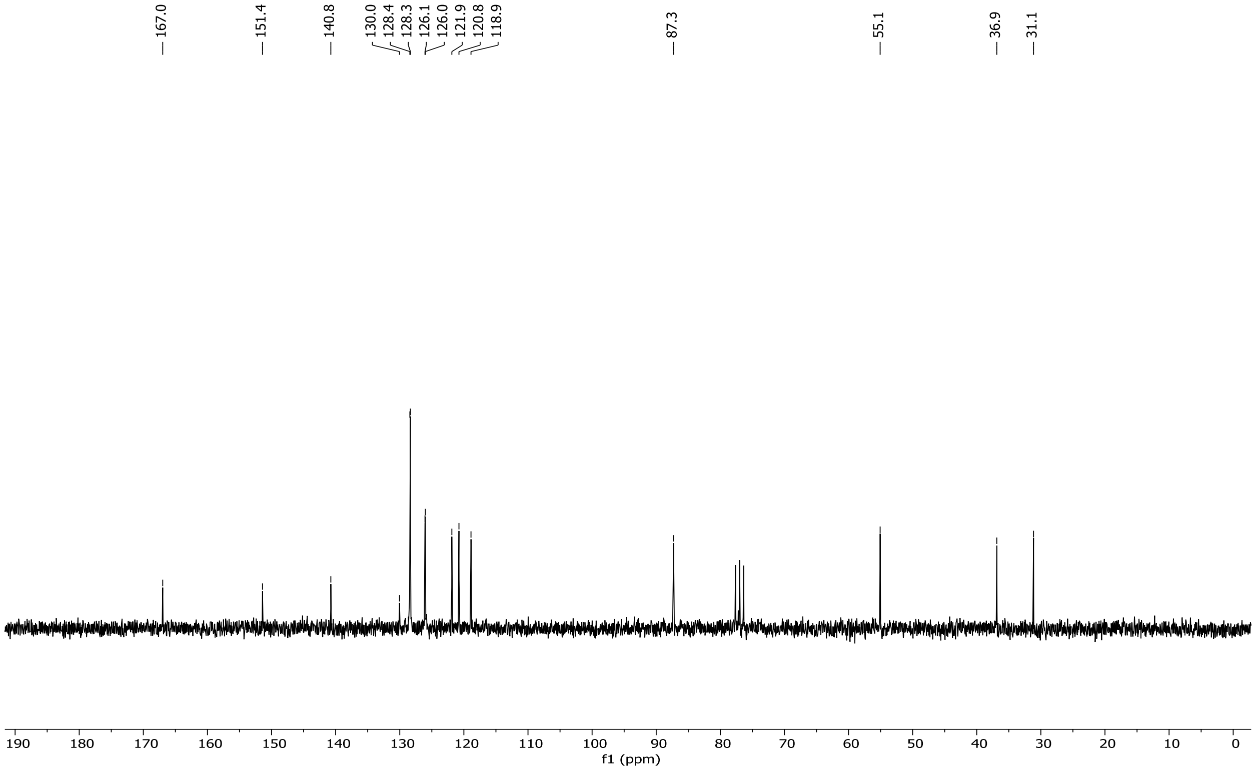

^{13}C NMR spectrum of **18b**.

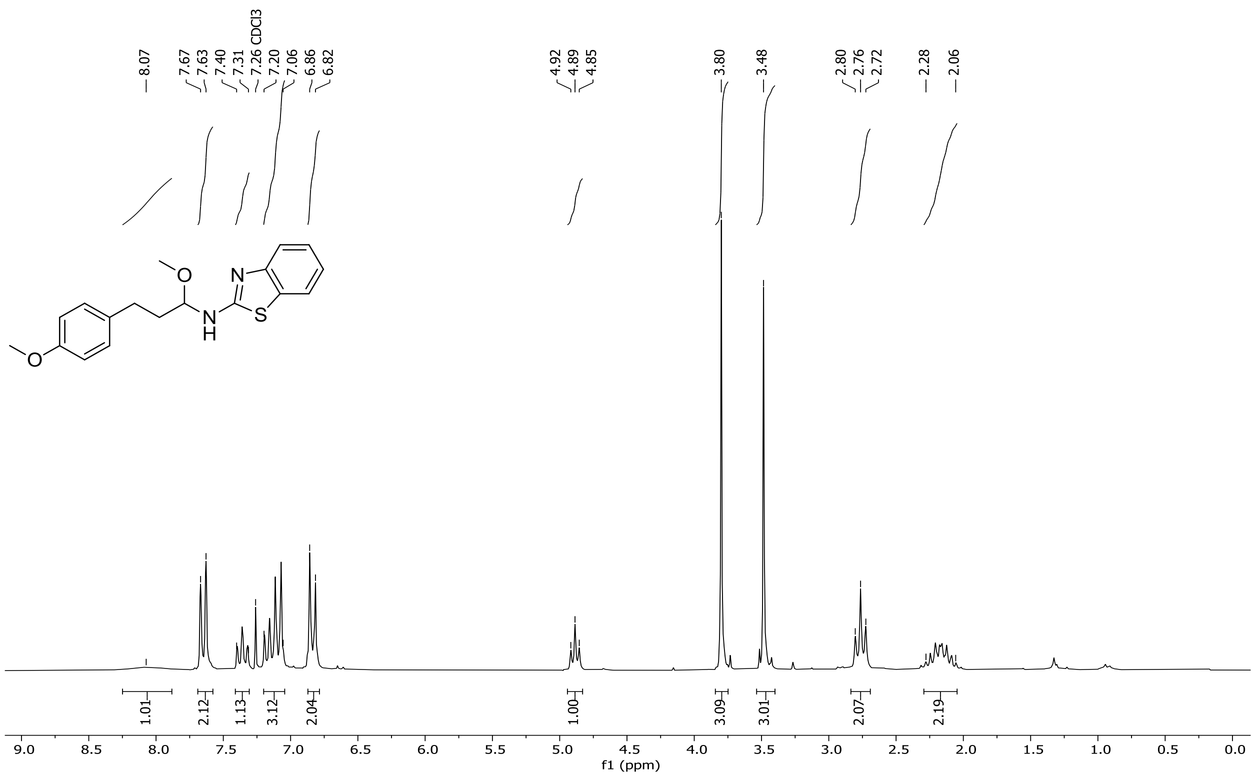

^{1}H NMR spectrum of **18c**.

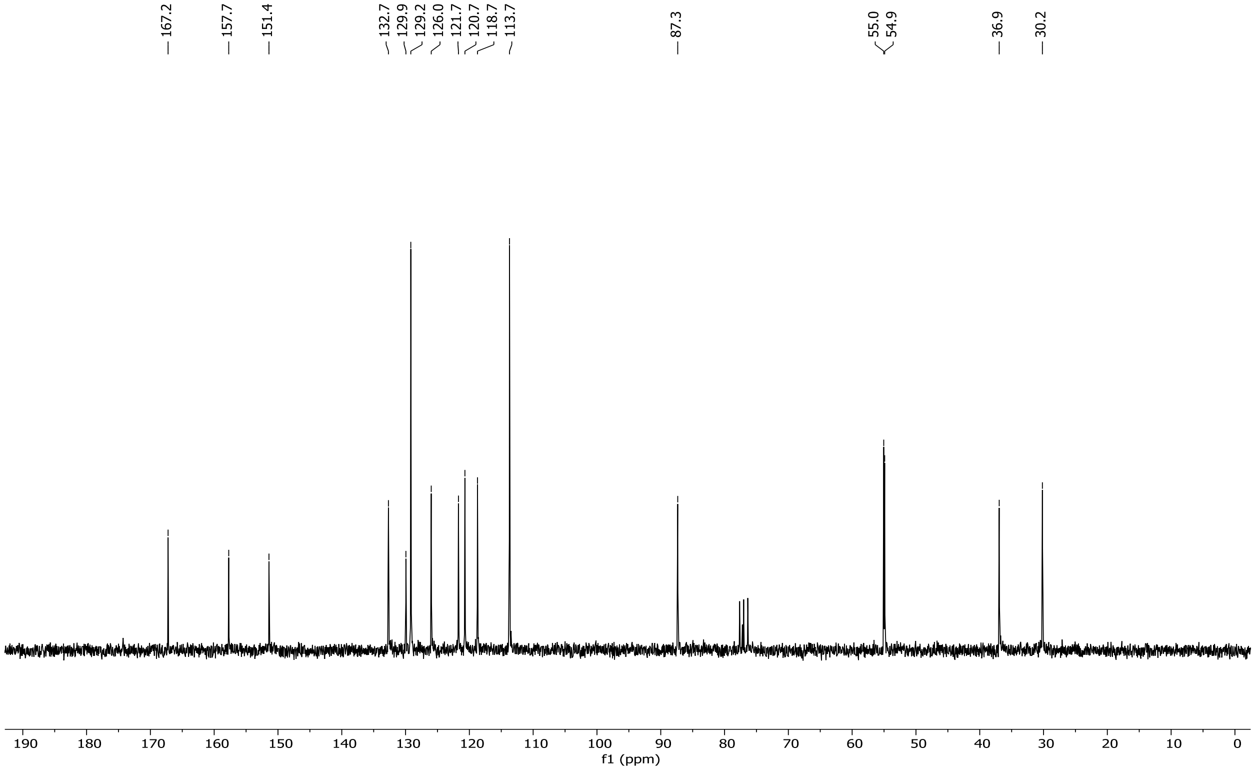

^{13}C NMR spectrum of **18c**.

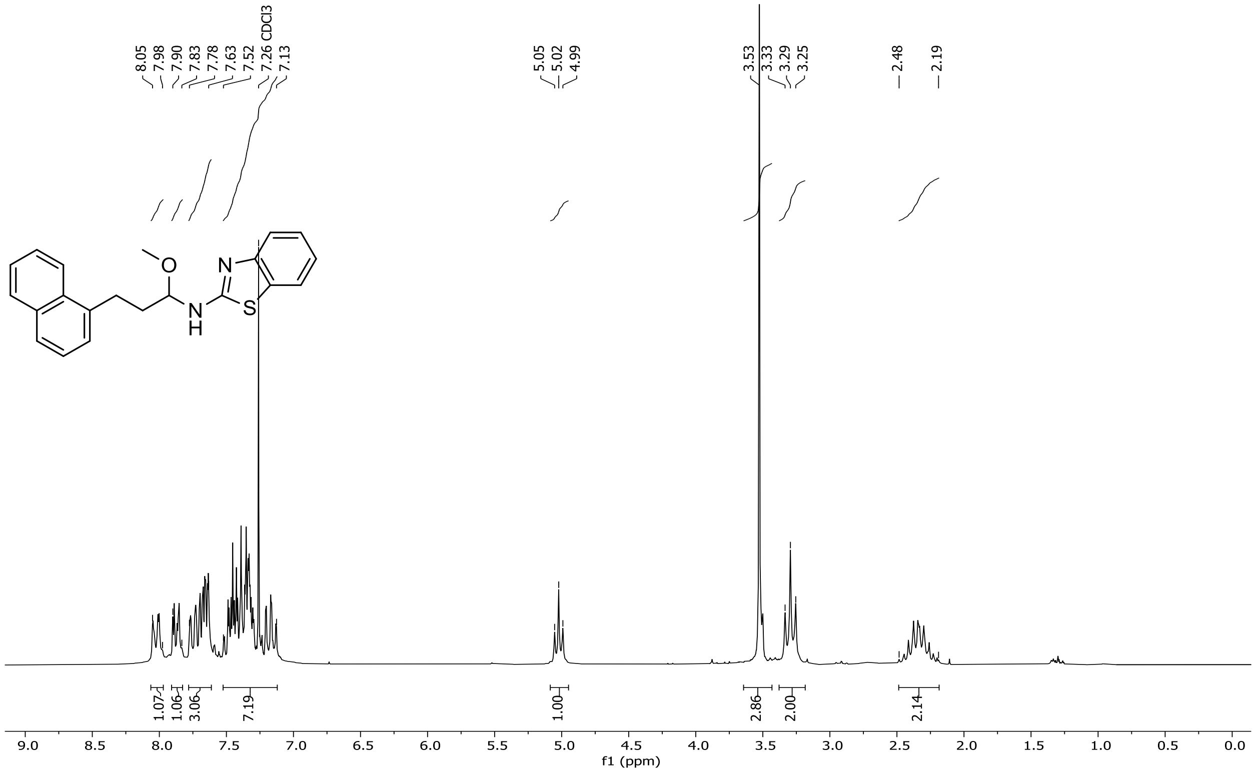

^{1}H NMR spectrum of **18d**.

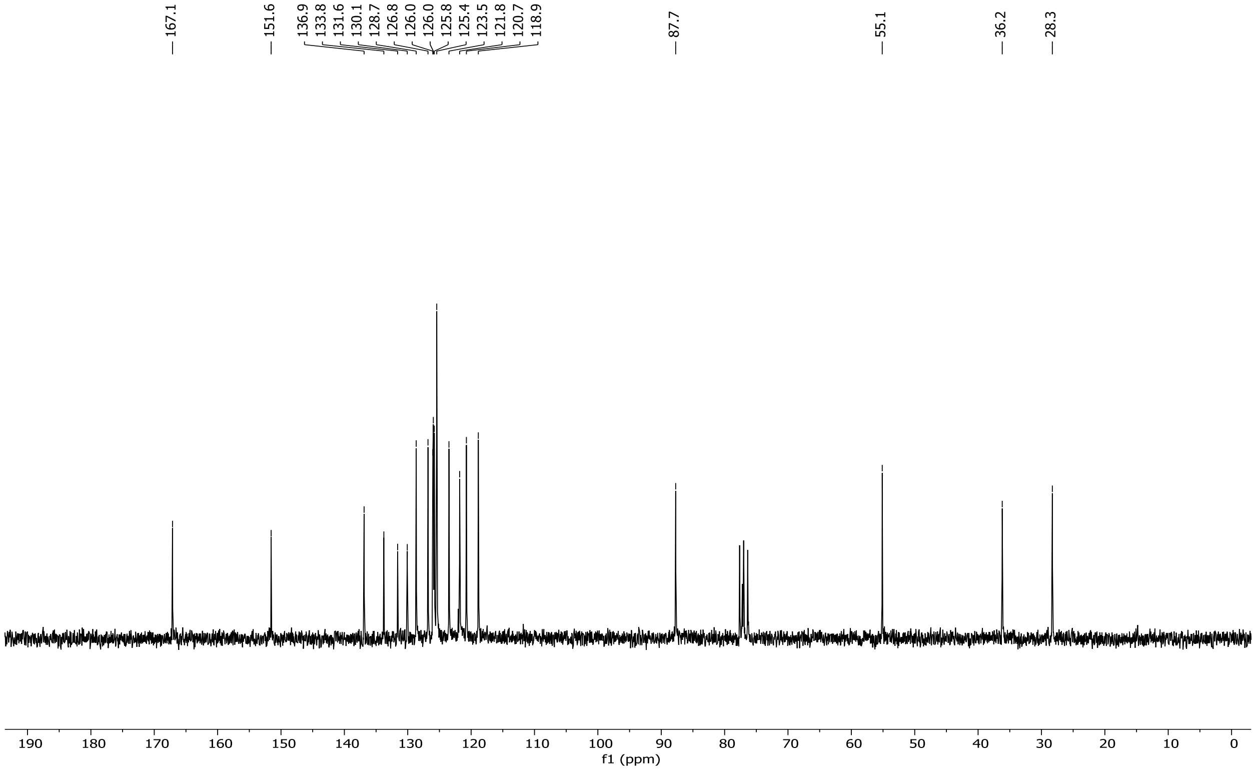

^{13}C NMR spectrum of **18d**.

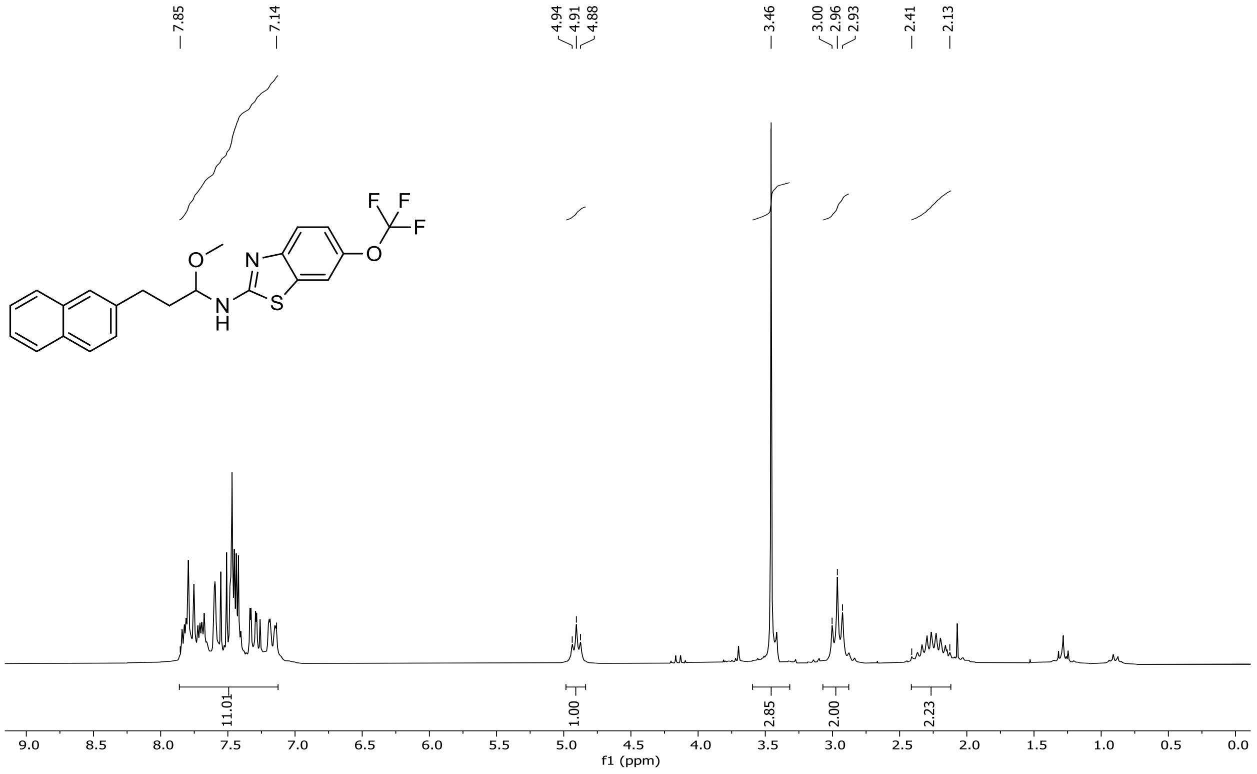

^{1}H NMR spectrum of **18g**.

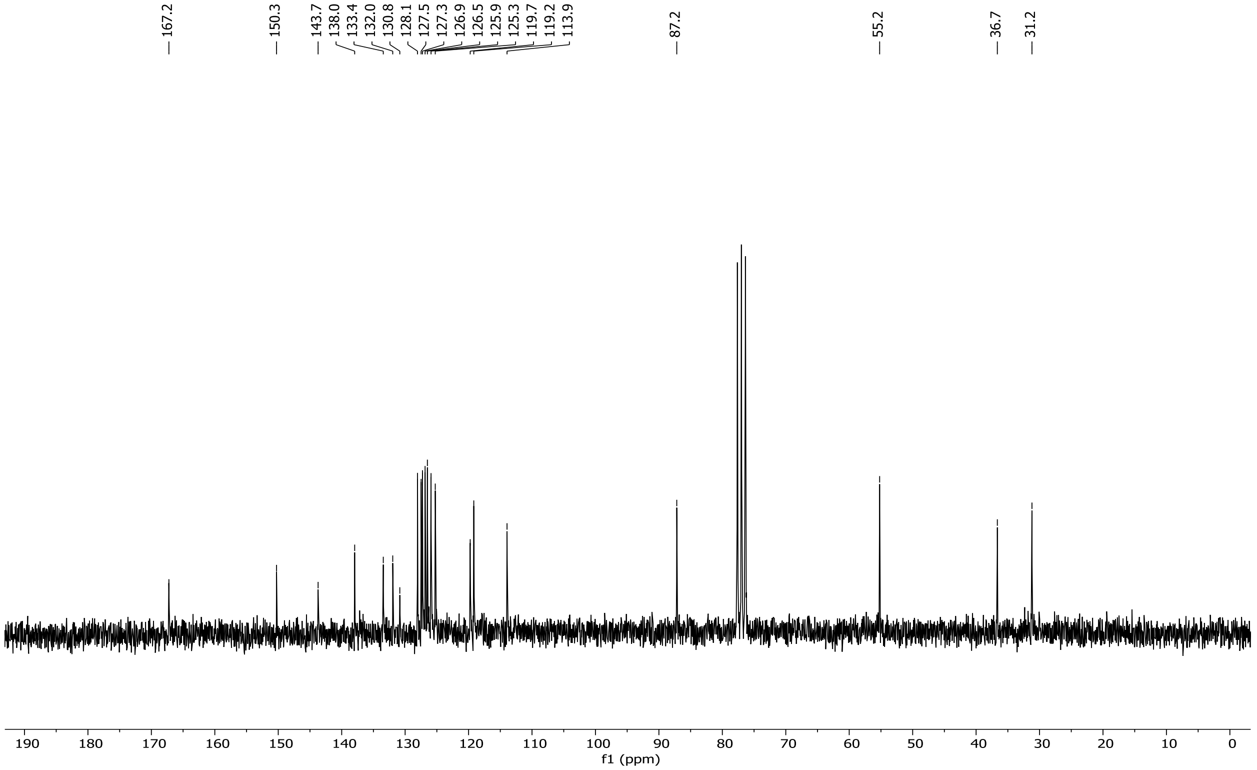

^{13}C NMR spectrum of **18g**.

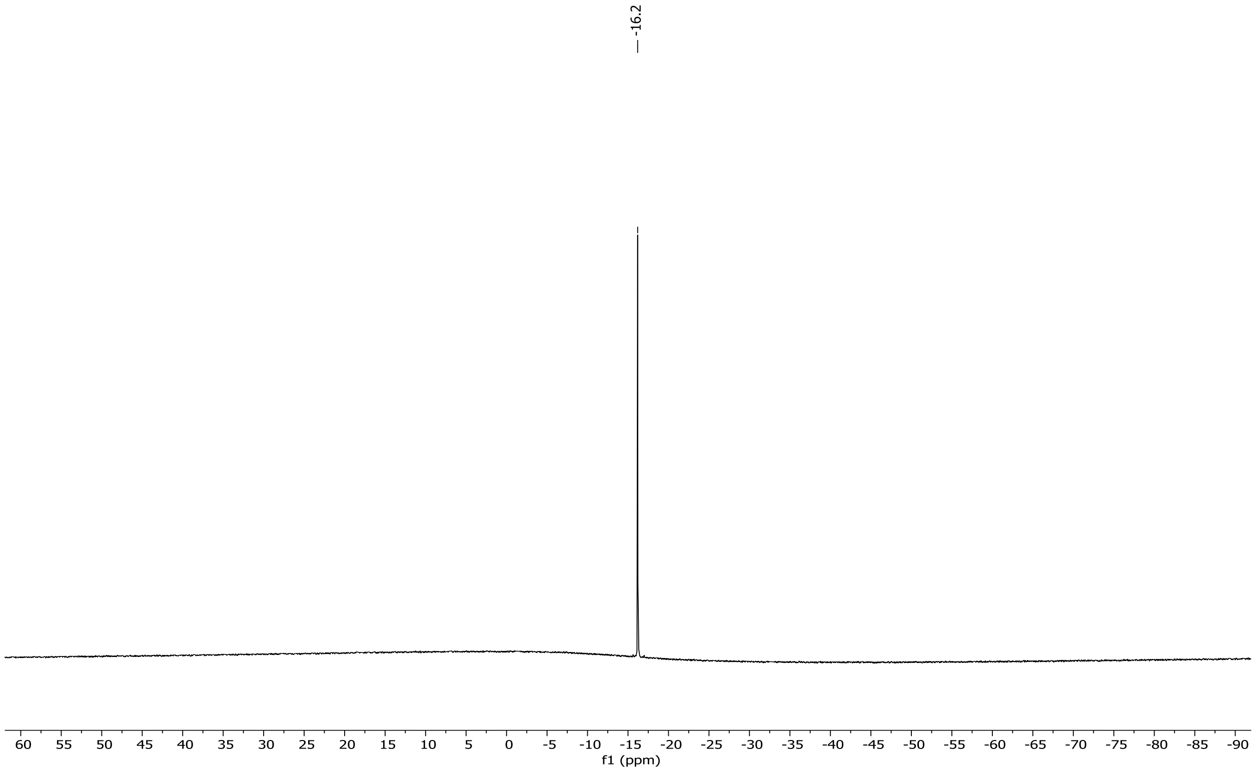

^{19}F NMR spectrum of **18g**.

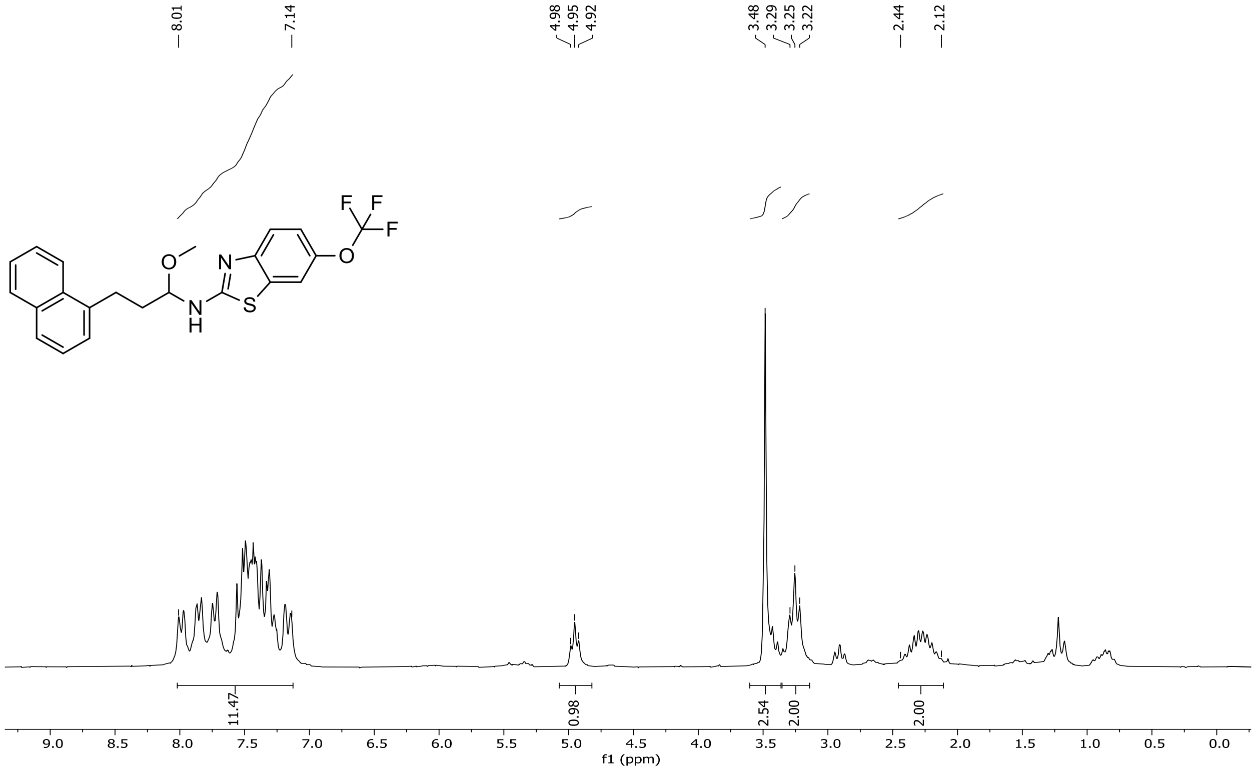

^{1}H NMR spectrum of **18h**.

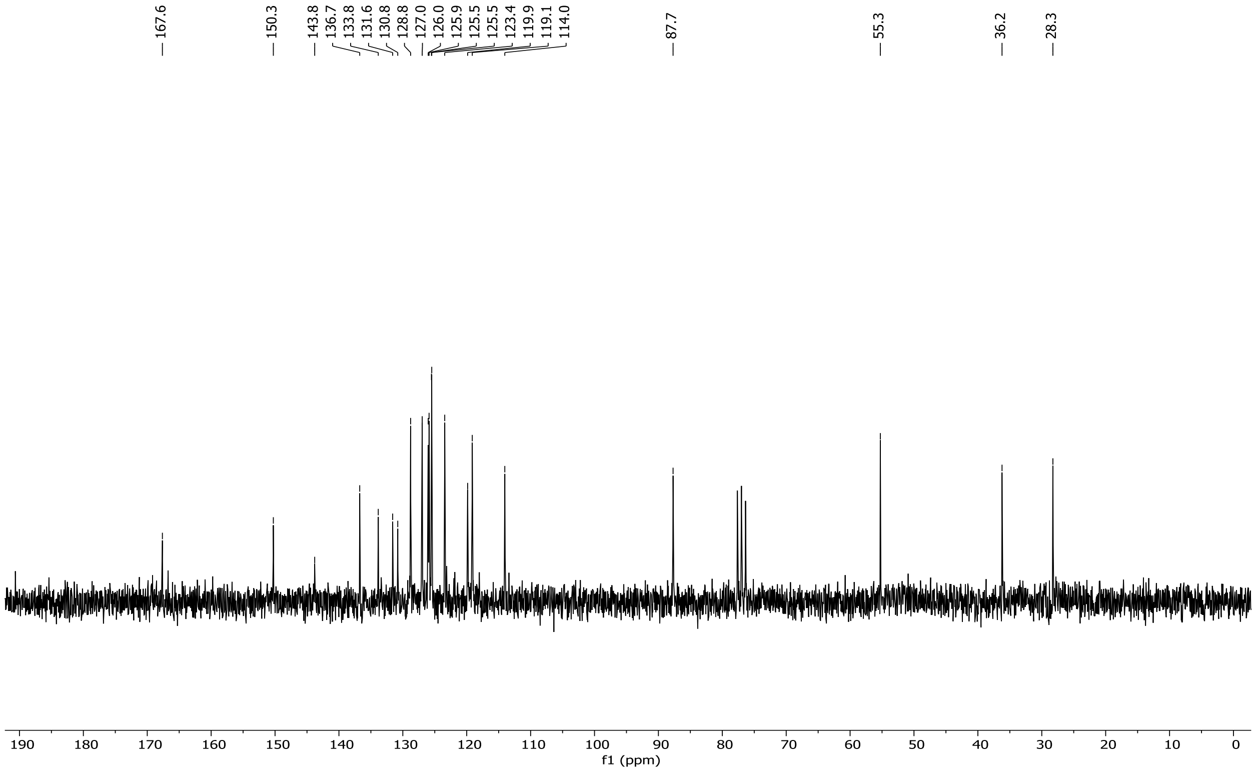

^{13}C NMR spectrum of **18h**.

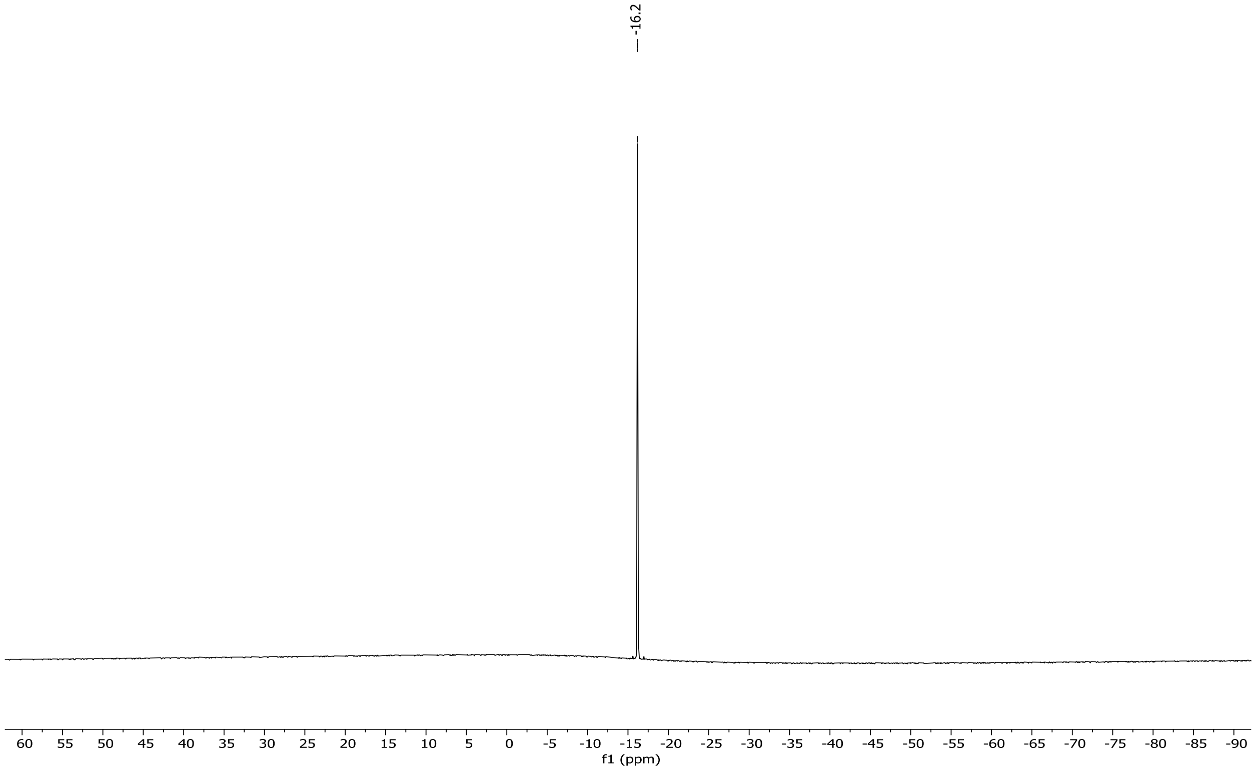

^{19}F NMR spectrum of **18h**.

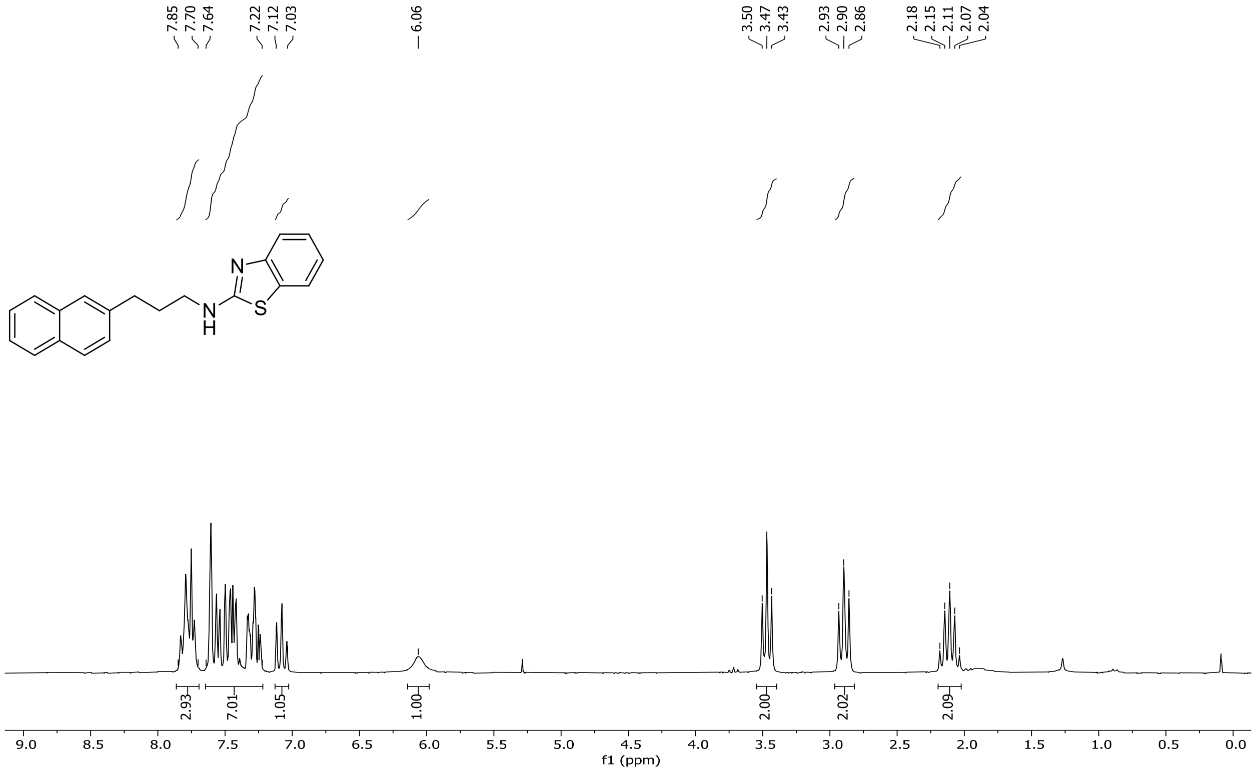

^{1}H NMR spectrum of **19a**.

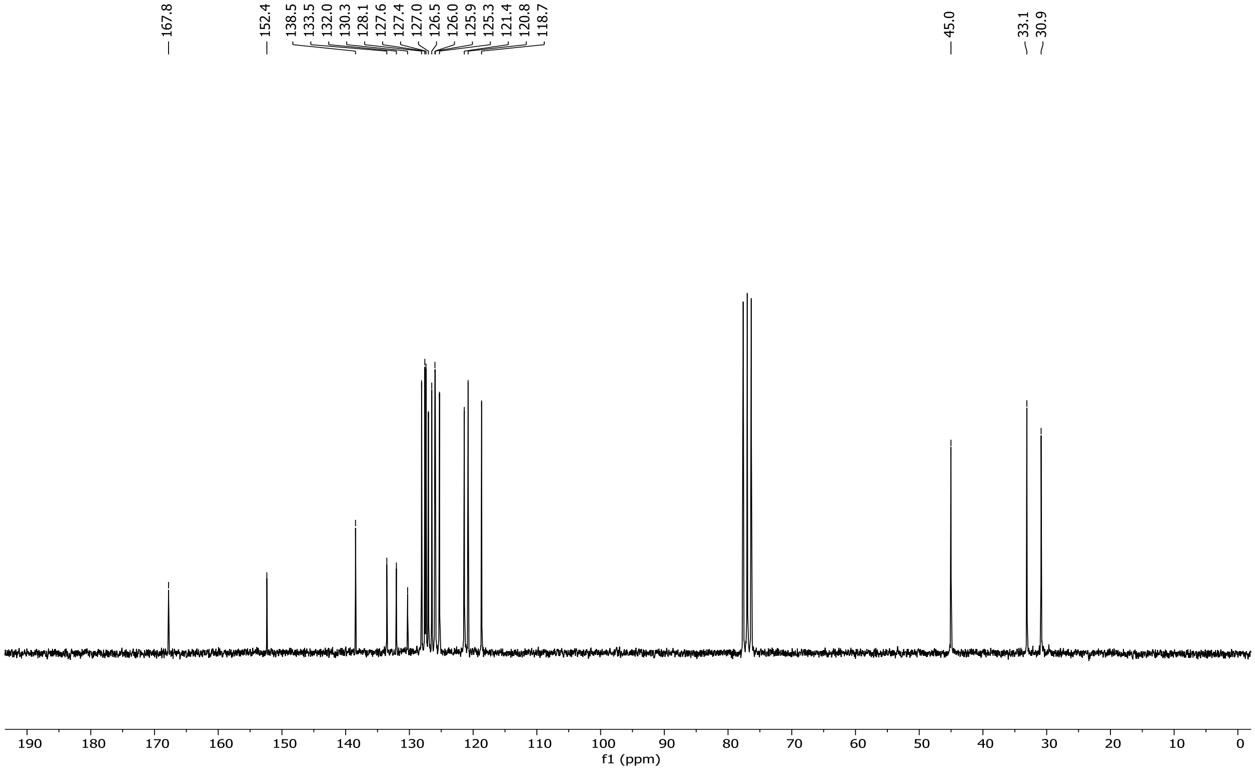

^{13}C NMR spectrum of **19a**.

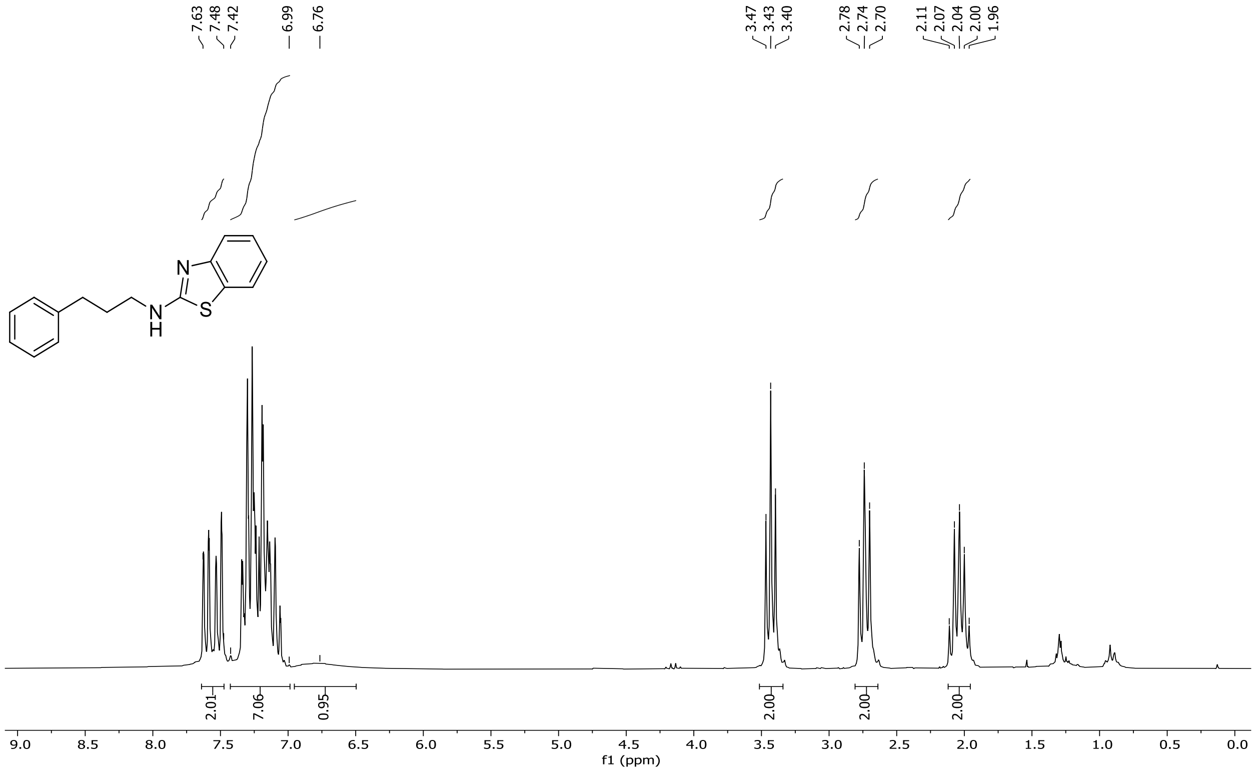

^{1}H NMR spectrum of **19b**.

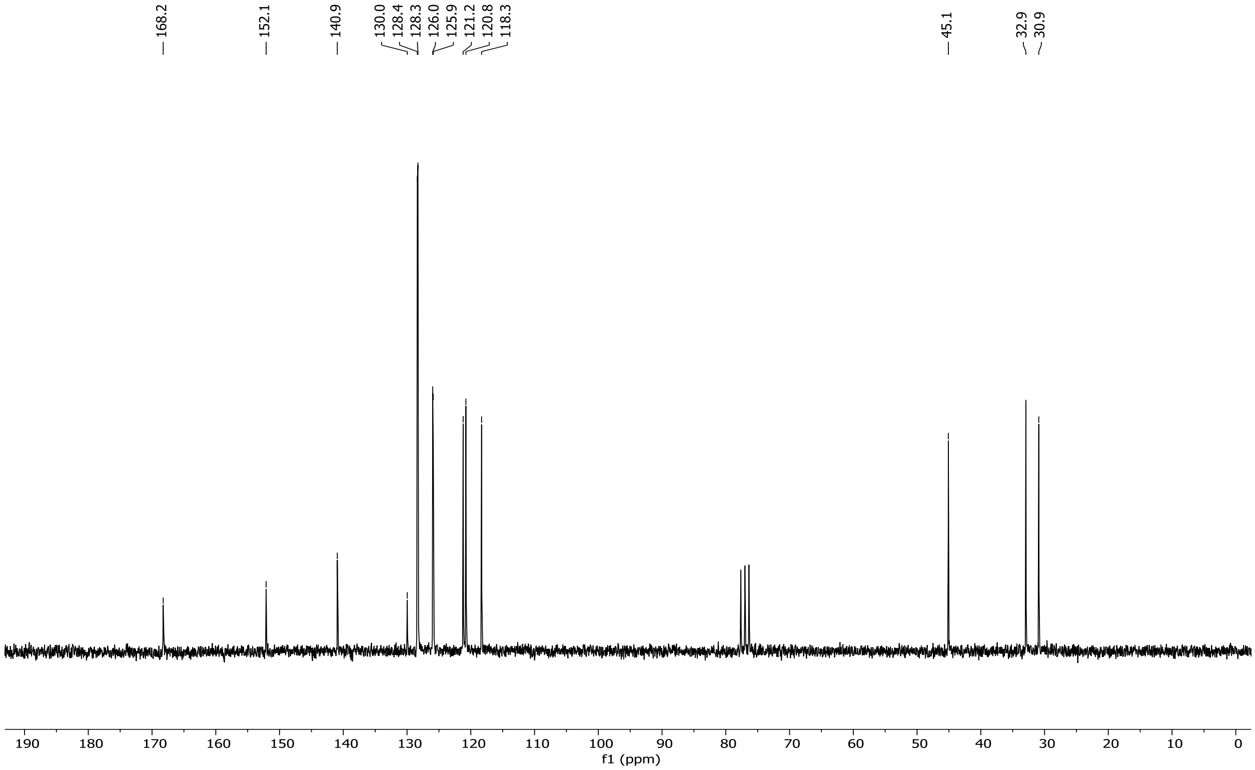

^{13}C NMR spectrum of **19b**.

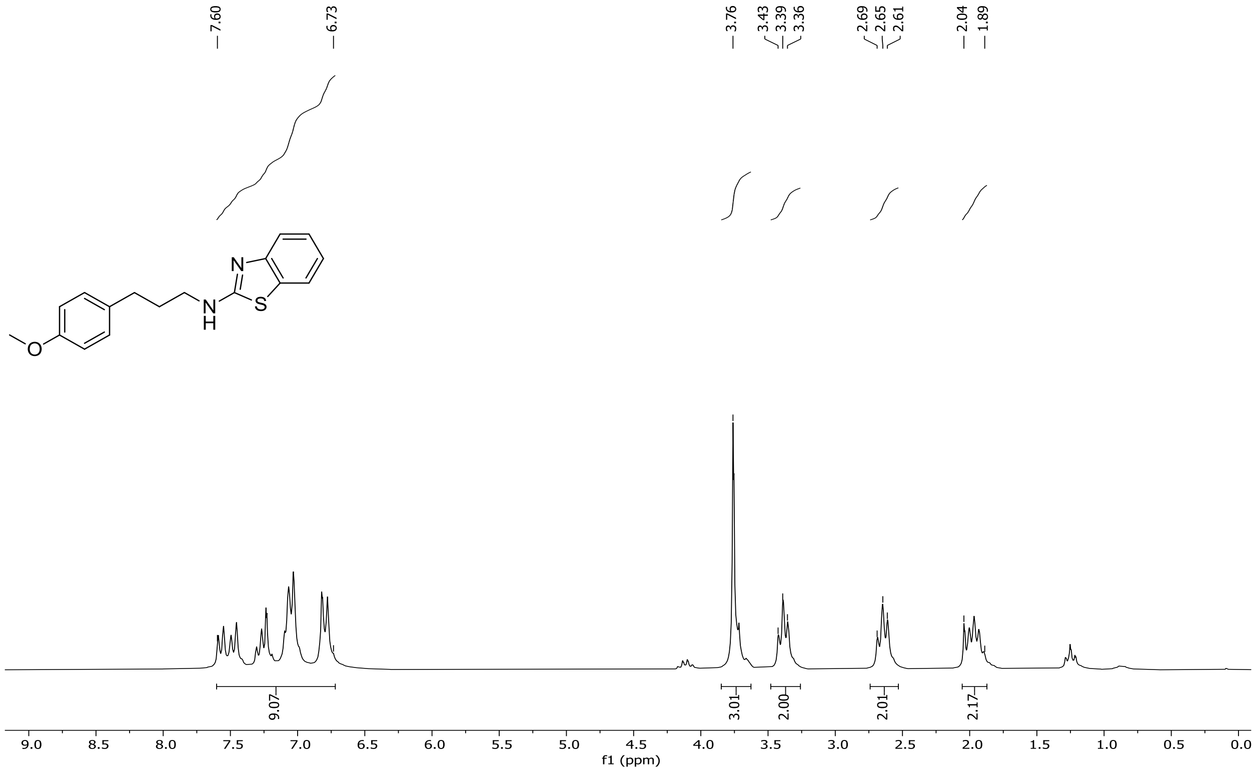

^{1}H NMR spectrum of **19c**.

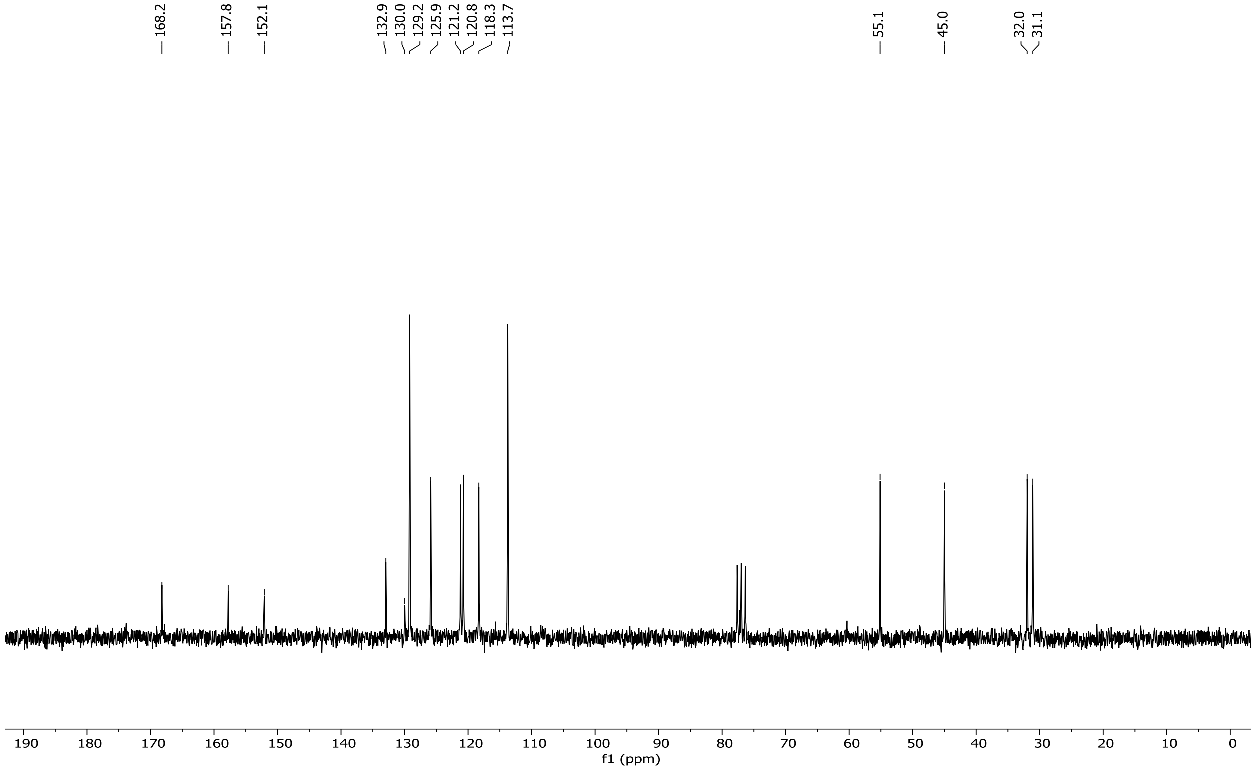

^{13}C NMR spectrum of **19c**.

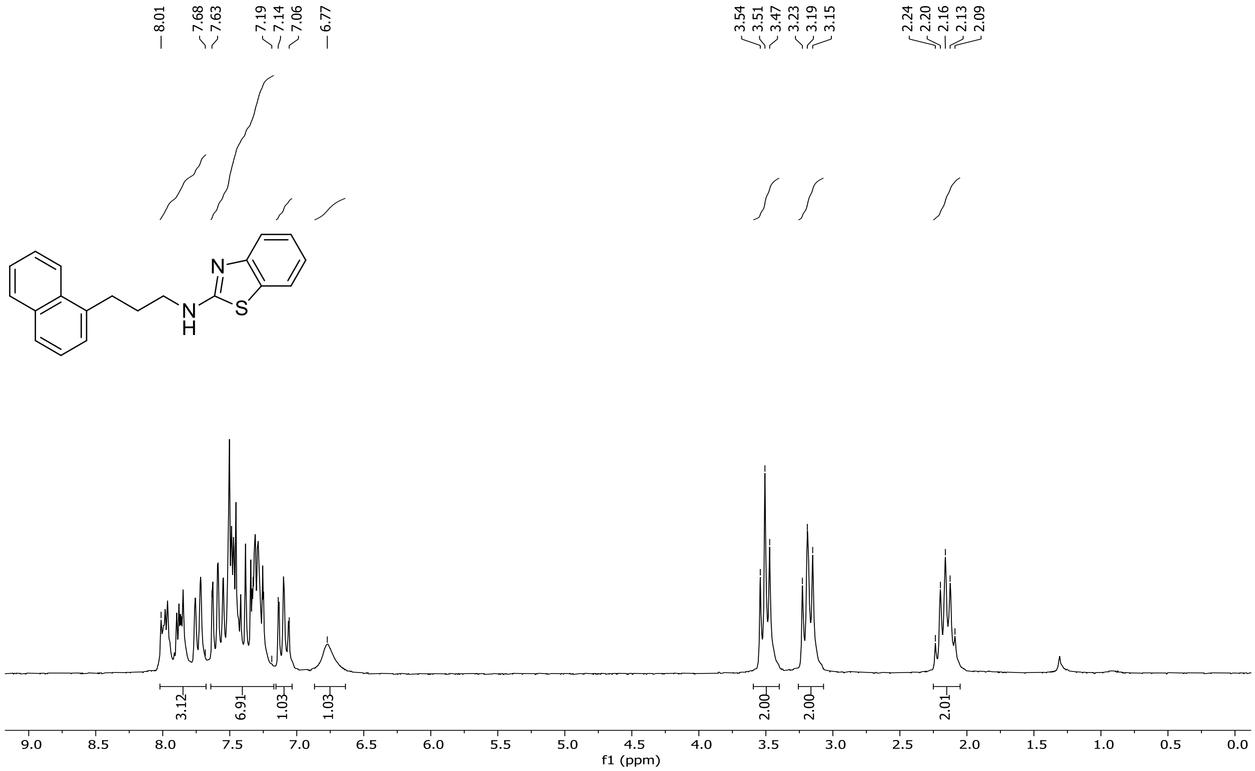

^{1}H NMR spectrum of **19d**.

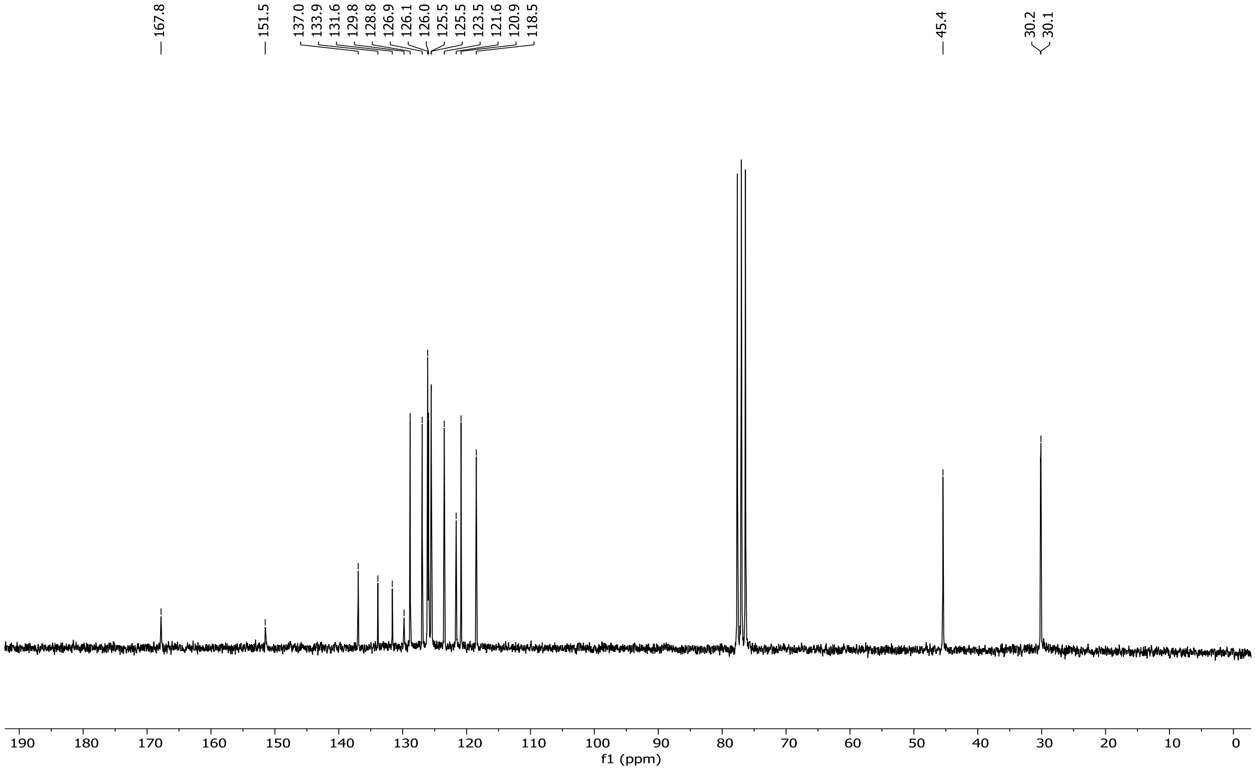

^{13}C NMR spectrum of **19d**.

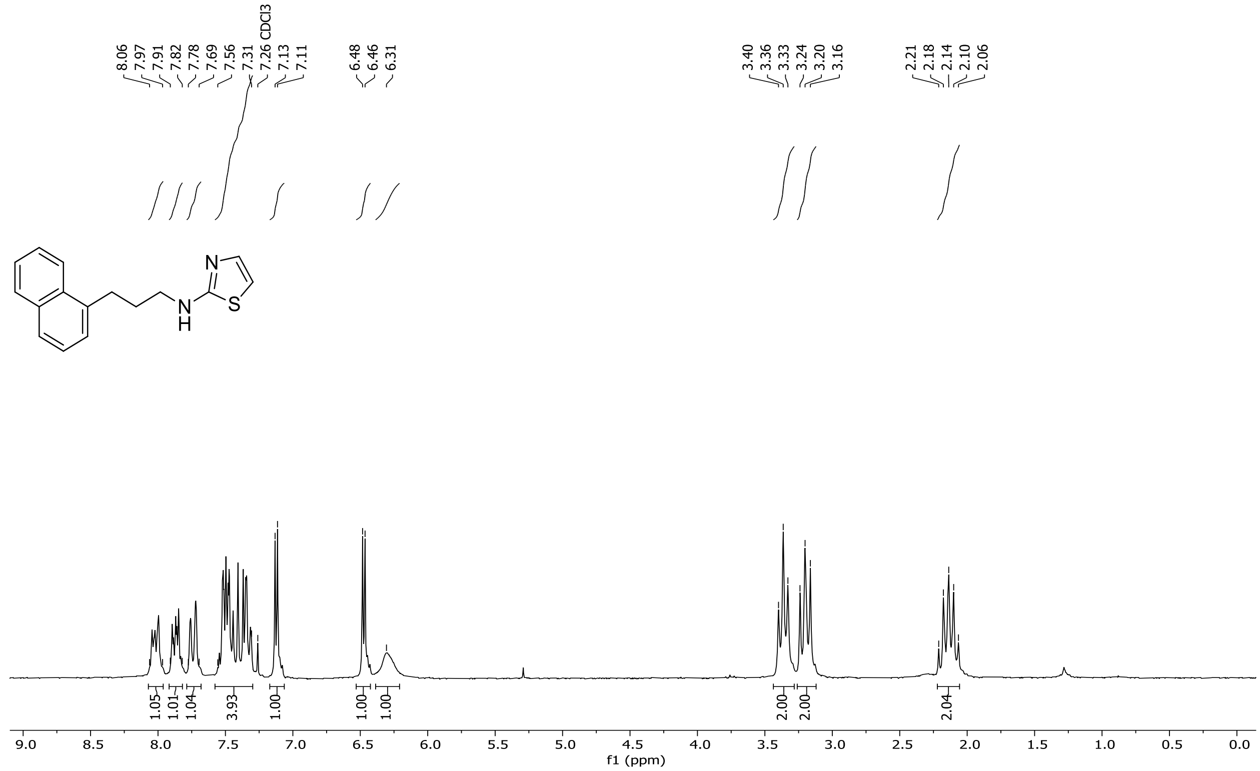

^{1}H NMR spectrum of **19e**.

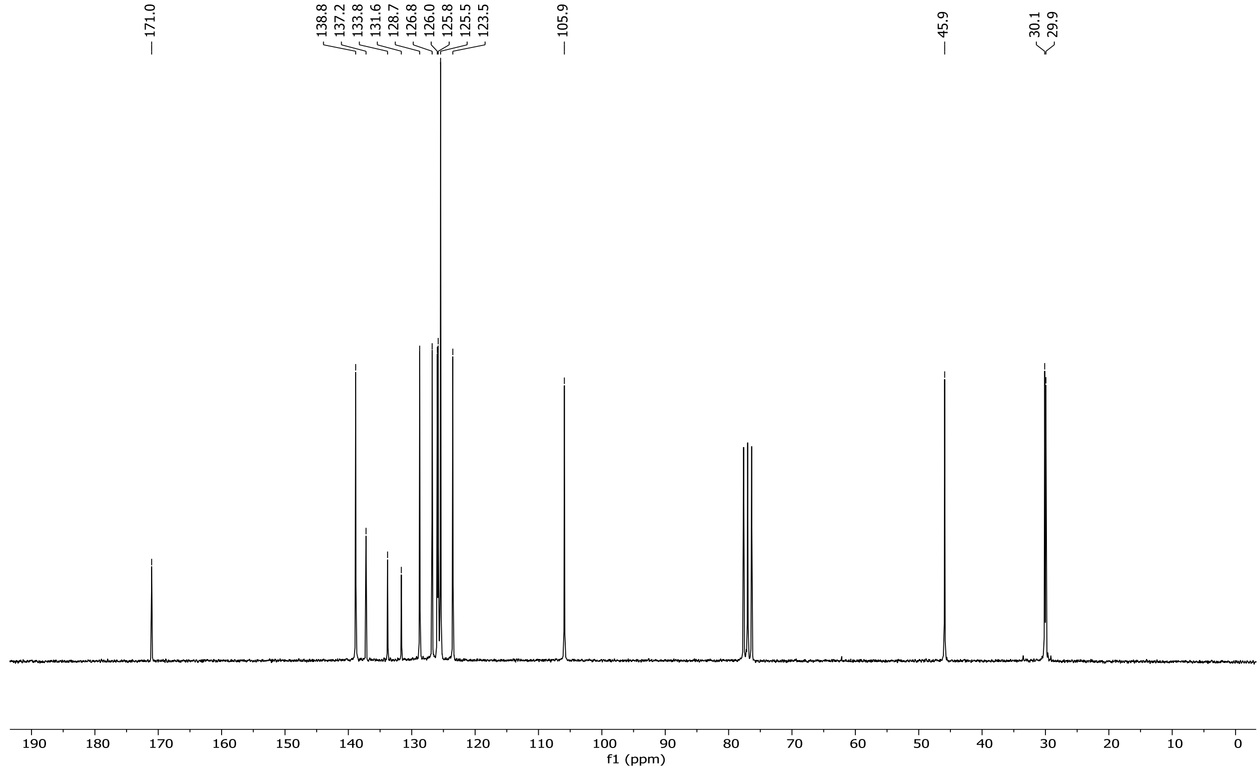

^{13}C NMR spectrum of **19e**.

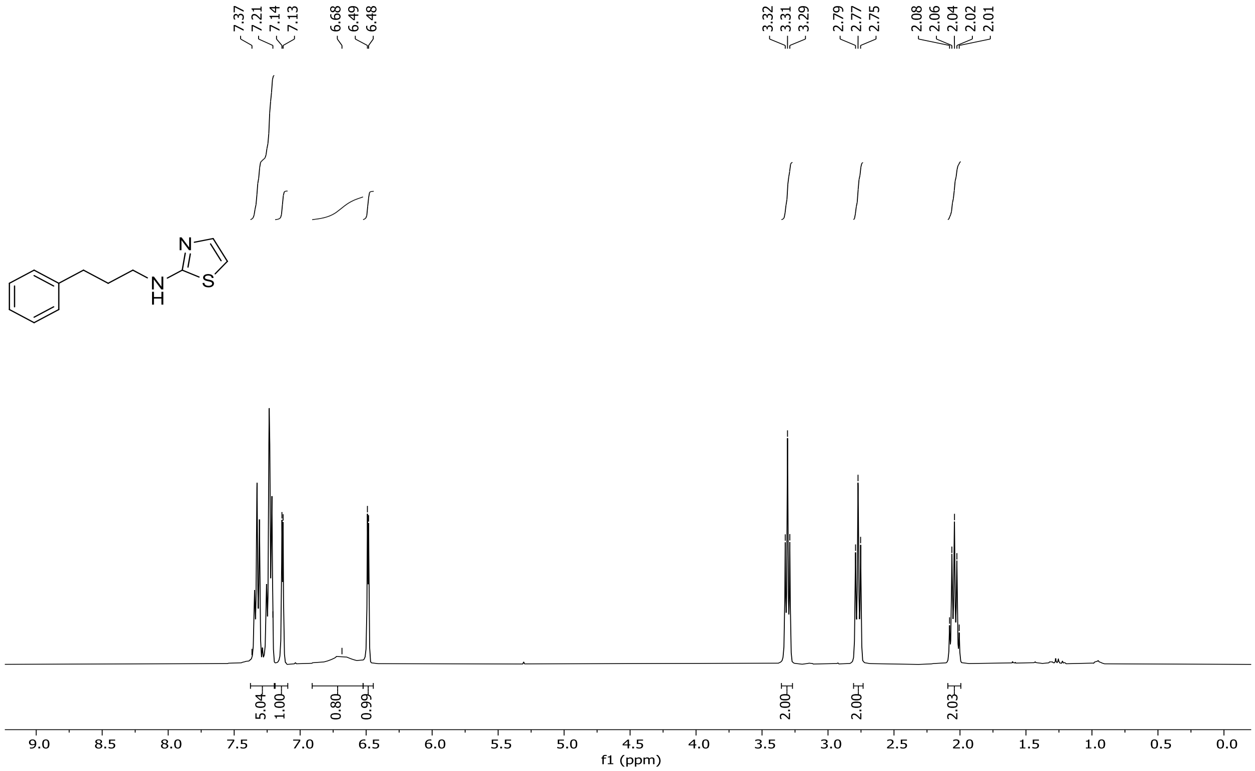

^{1}H NMR spectrum of **19f**.

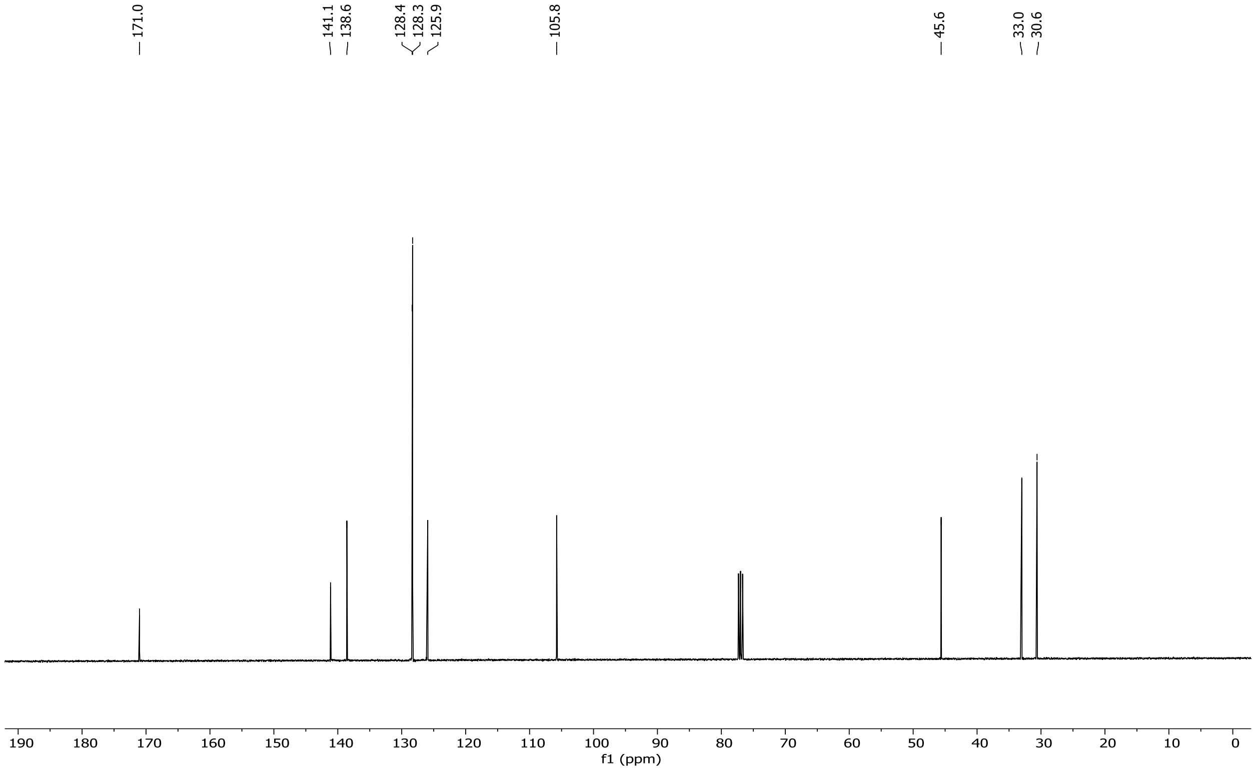

^{13}C NMR spectrum of **19f**.

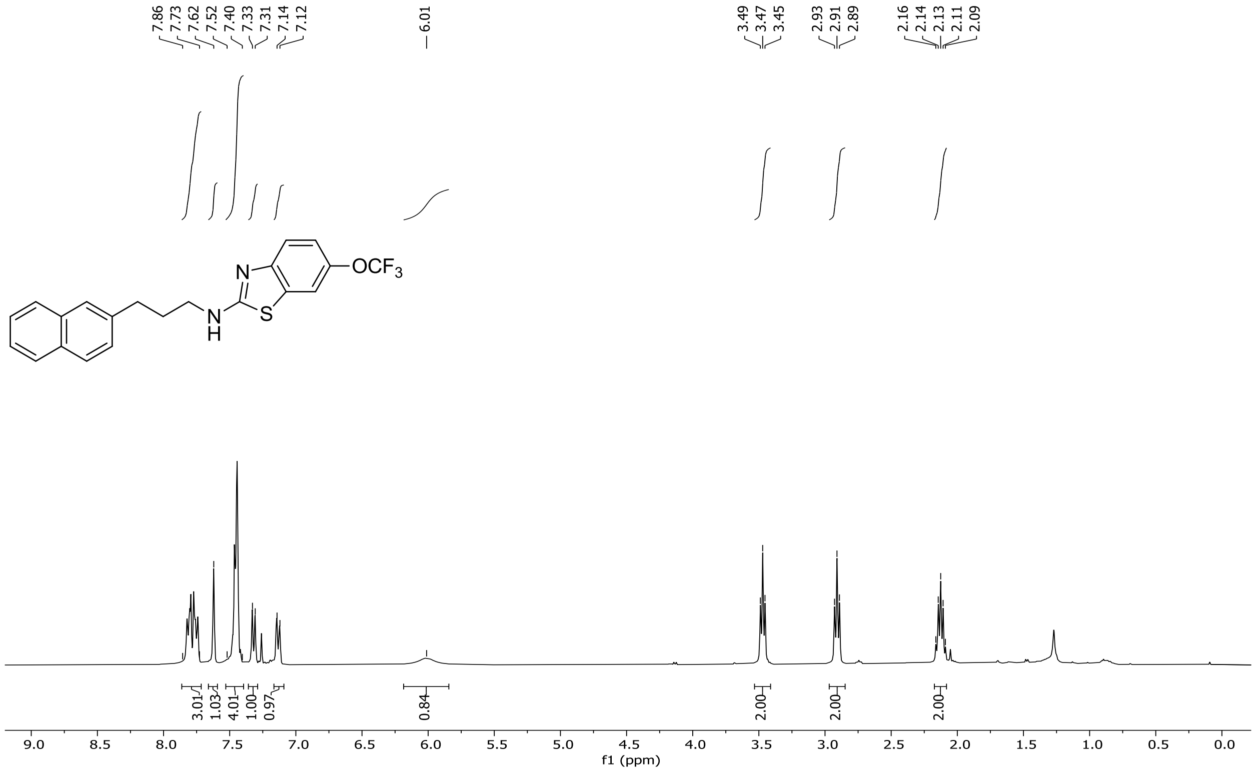

^{1}H NMR spectrum of **19g**.

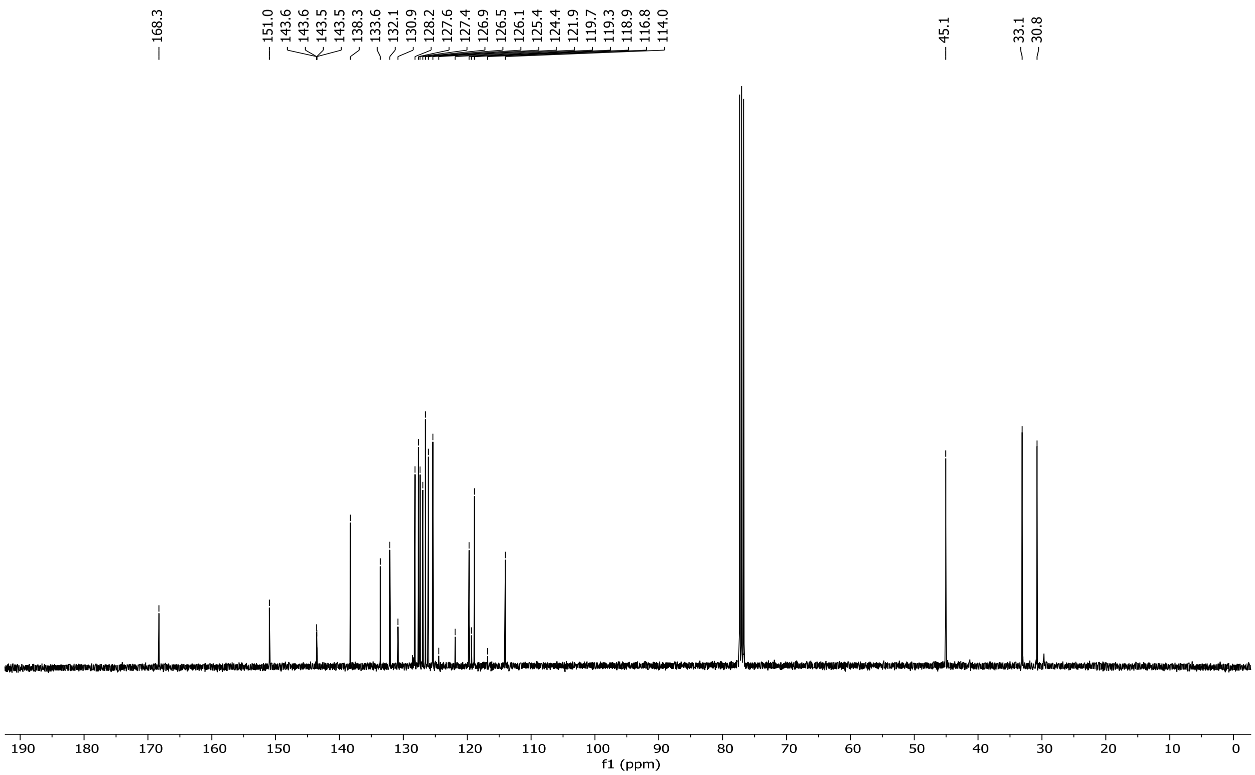

^{13}C NMR spectrum of **19g**.

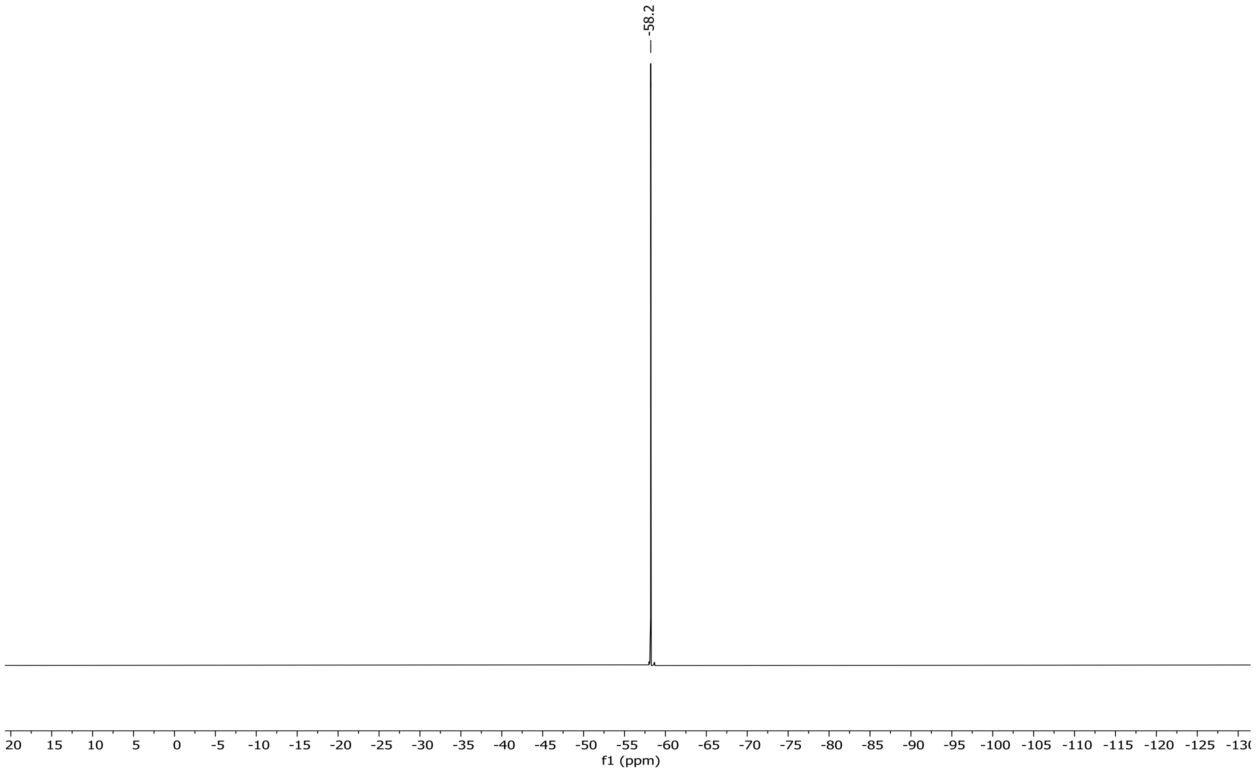

^{19}F NMR spectrum of **19g**.

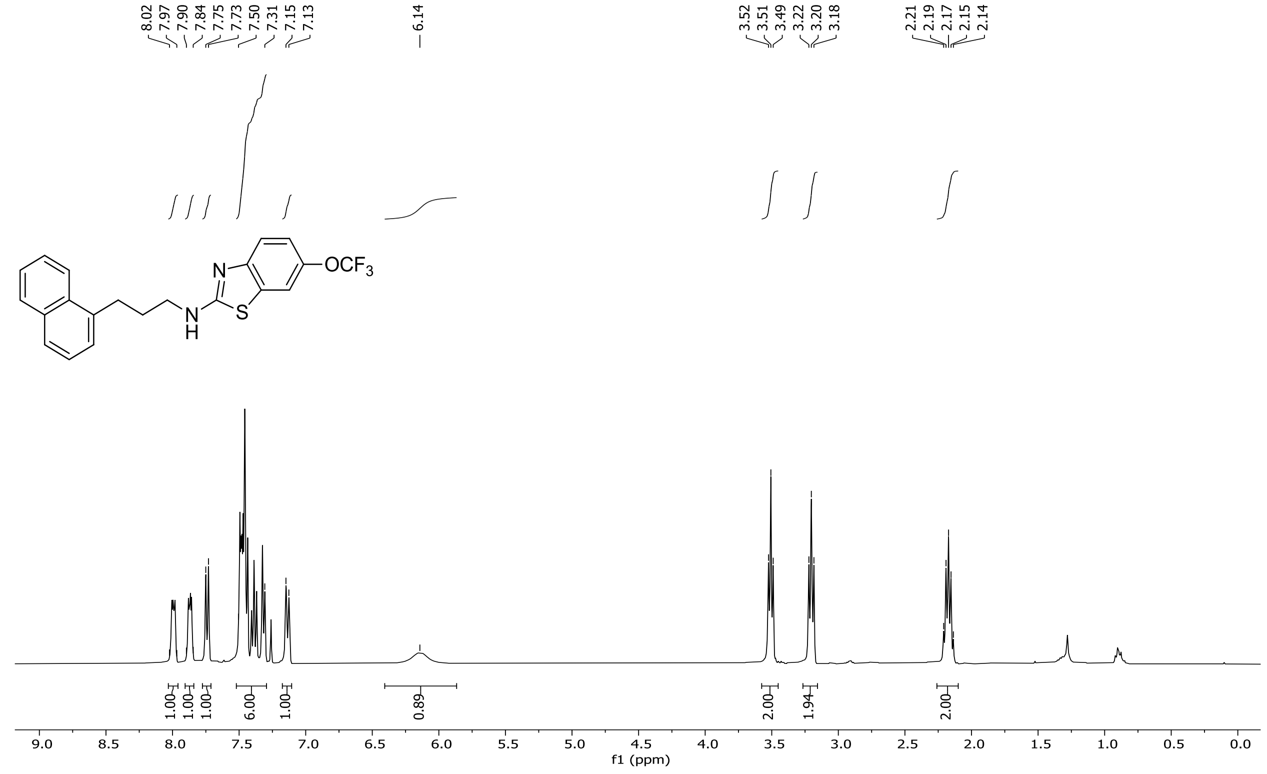

^{1}H NMR spectrum of **19h**.

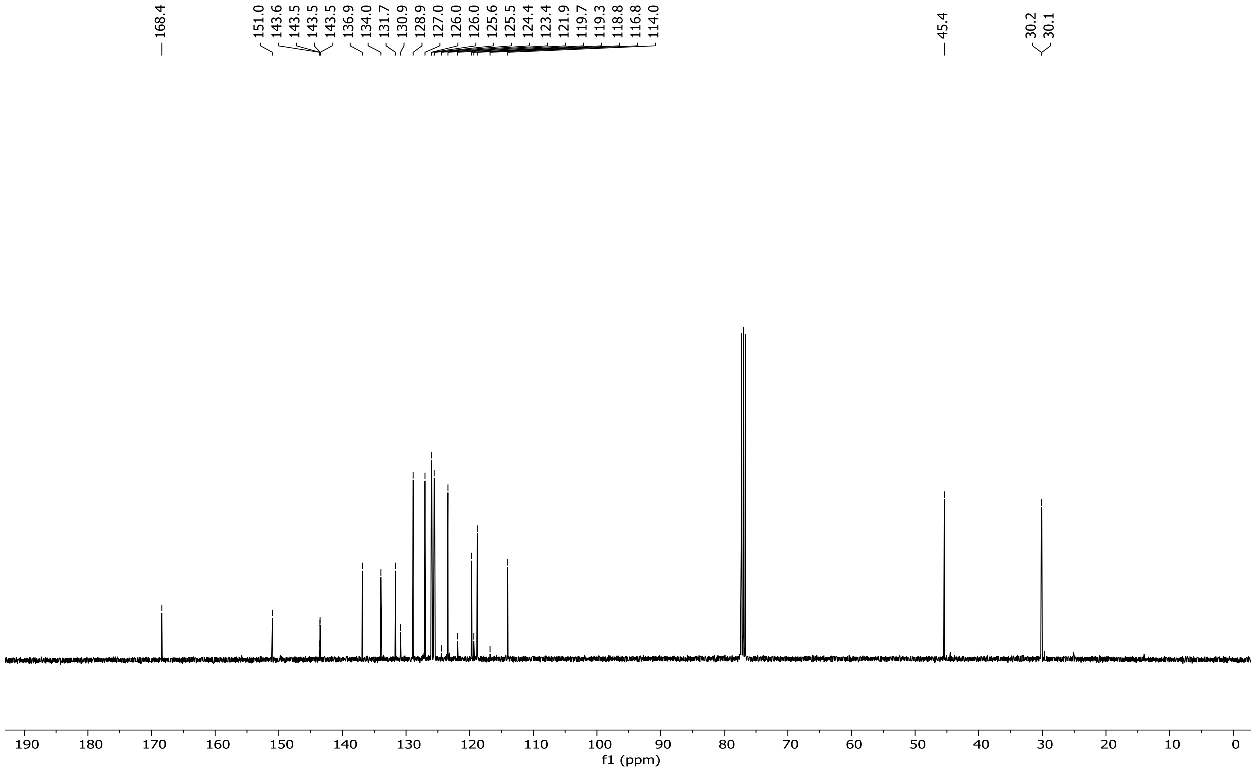

^{13}C NMR spectrum of **19h**.

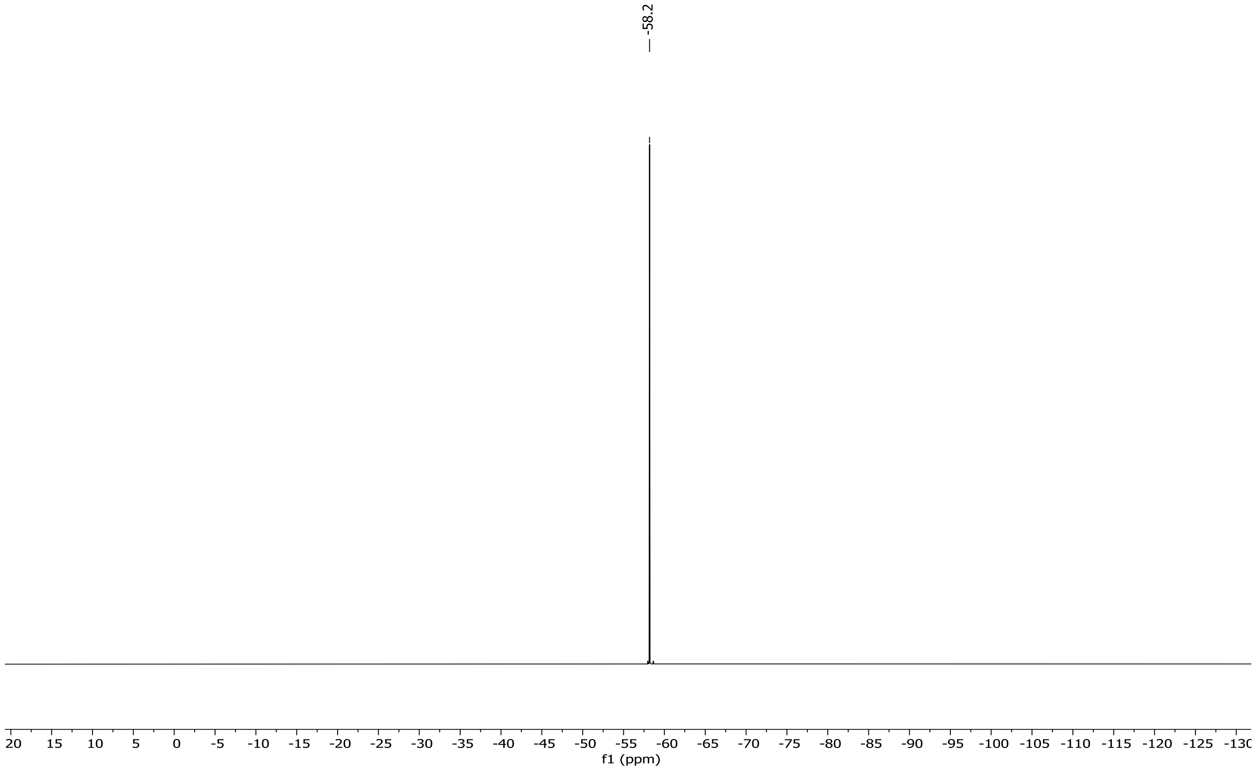

^{19}F NMR spectrum of **19h**.

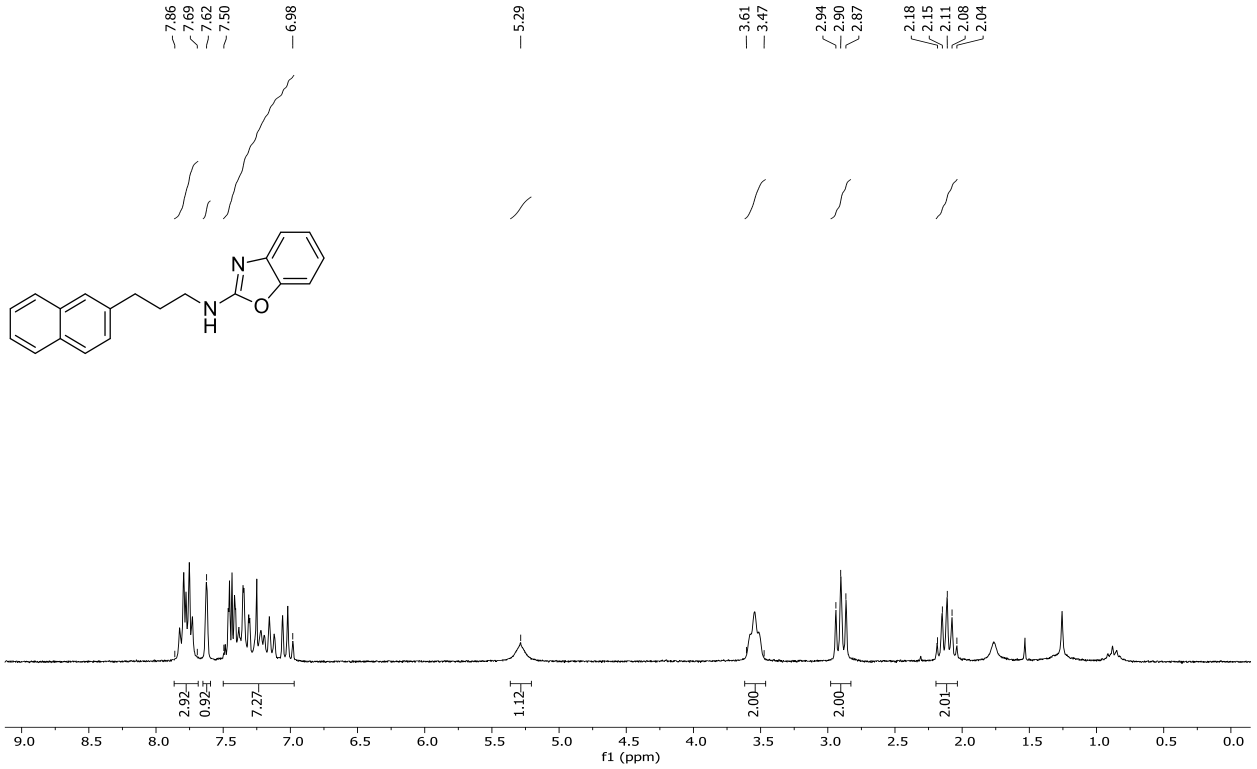

^{1}H NMR spectrum of **23a**.

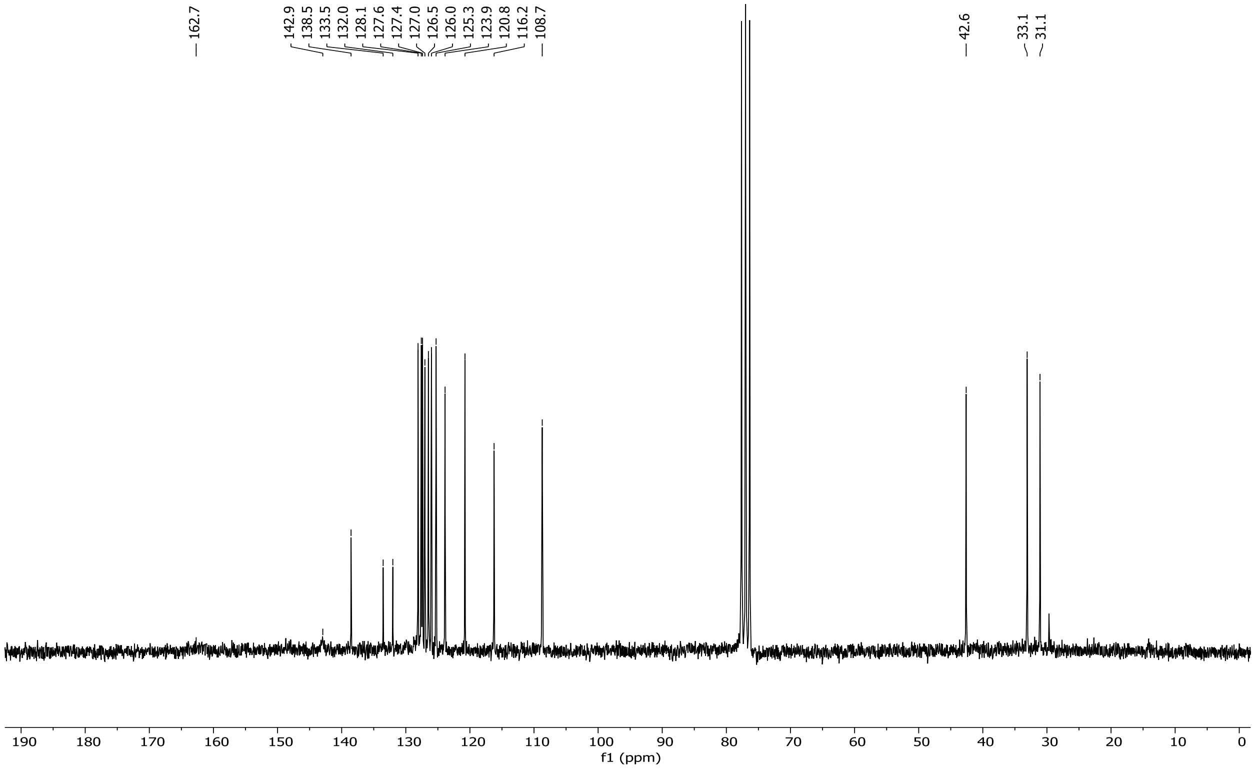

^{13}C NMR spectrum of **23a**.

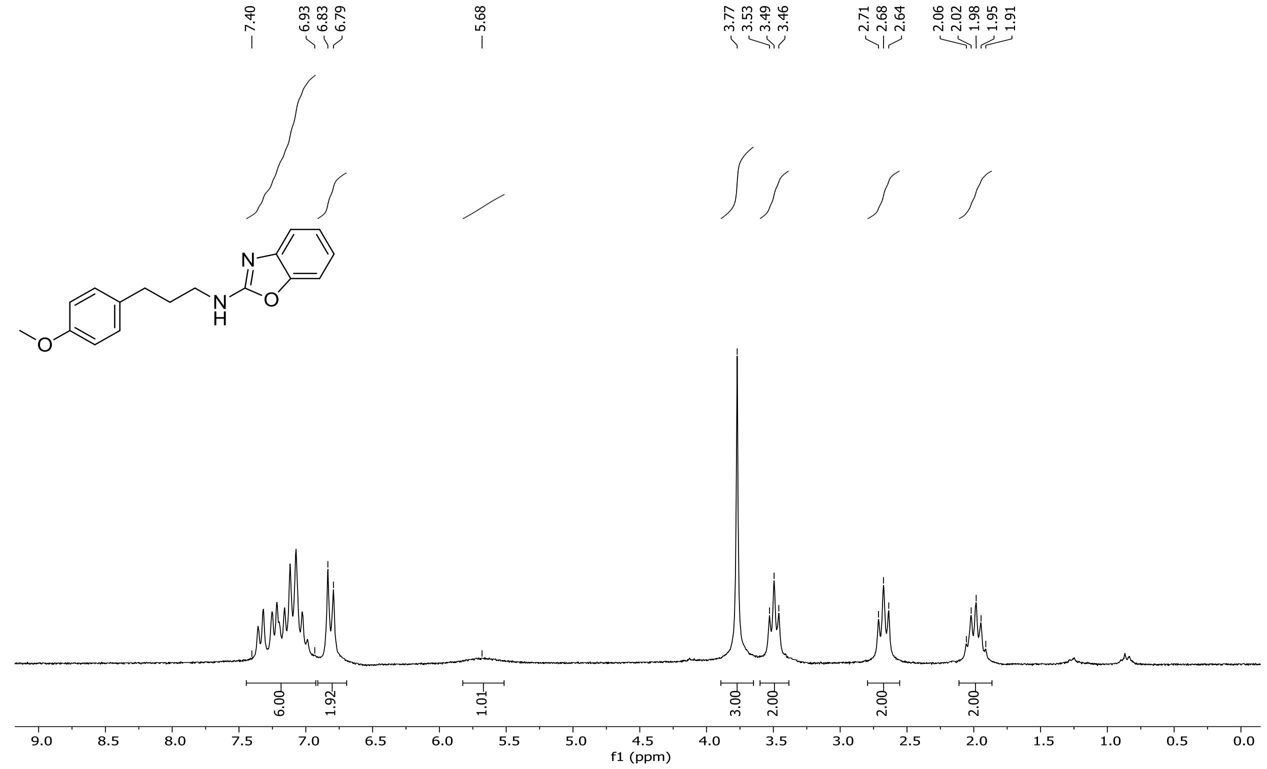

^{1}H NMR spectrum of **23b**.

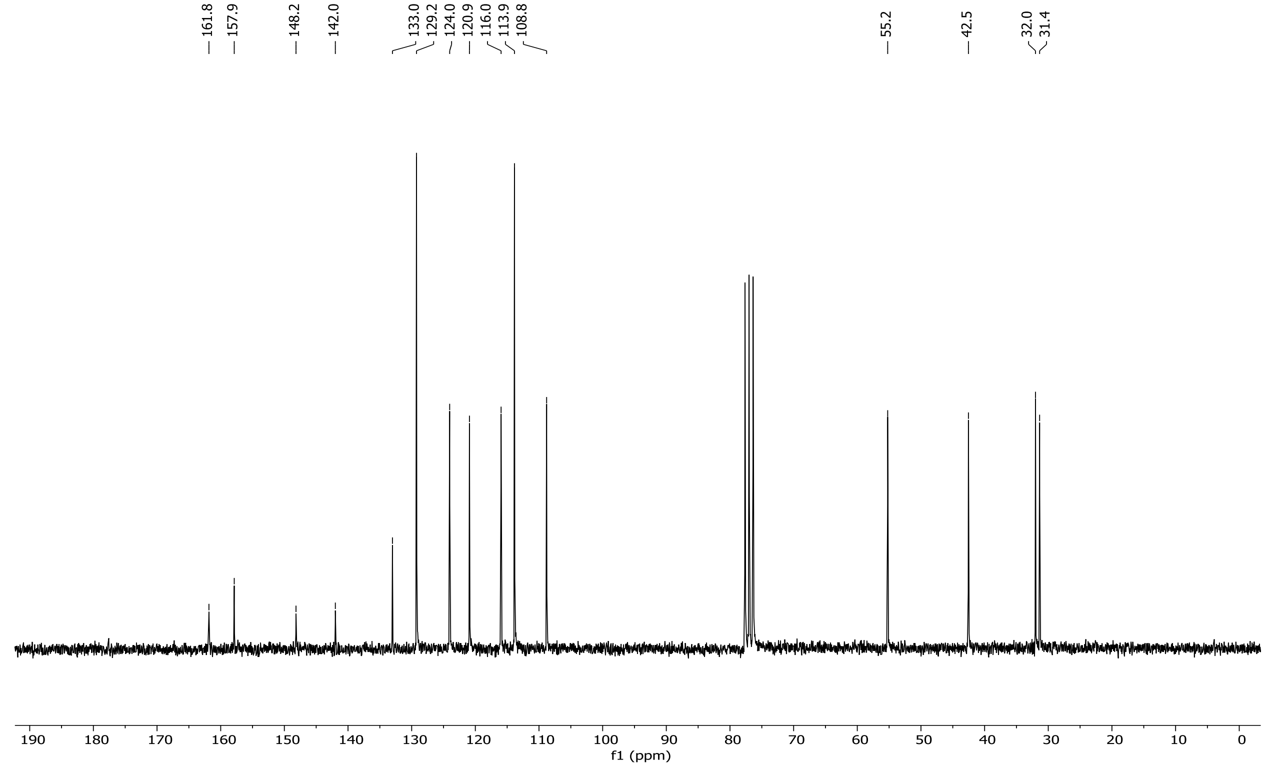

^{13}C NMR spectrum of **23b**.

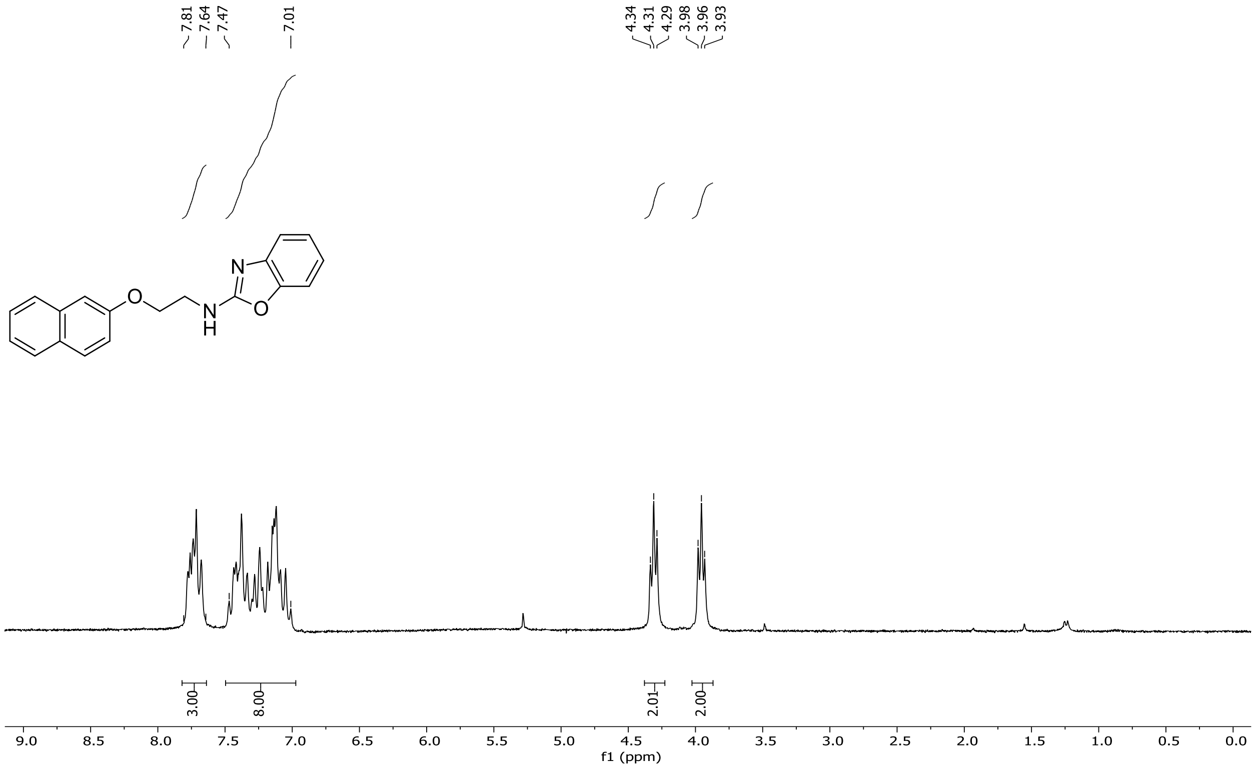

^{1}H NMR spectrum of **23c**.

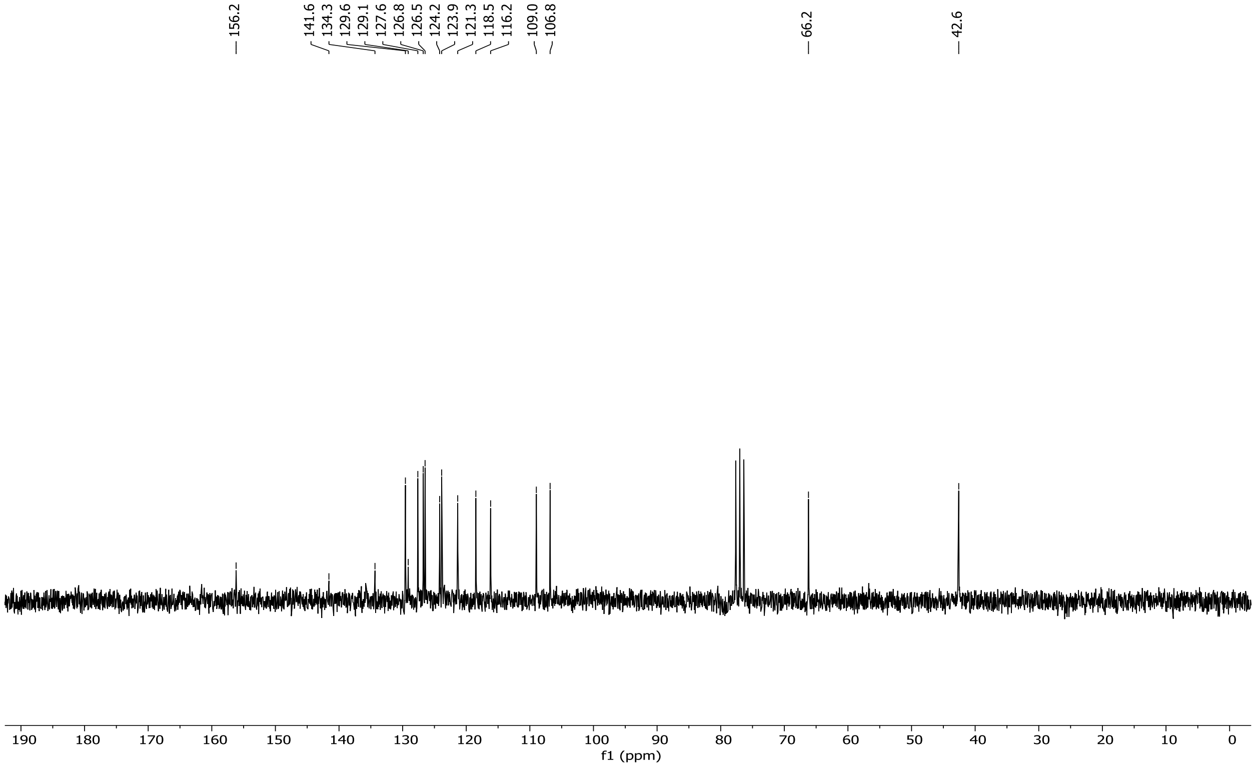

^{13}C NMR spectrum of **23c**.

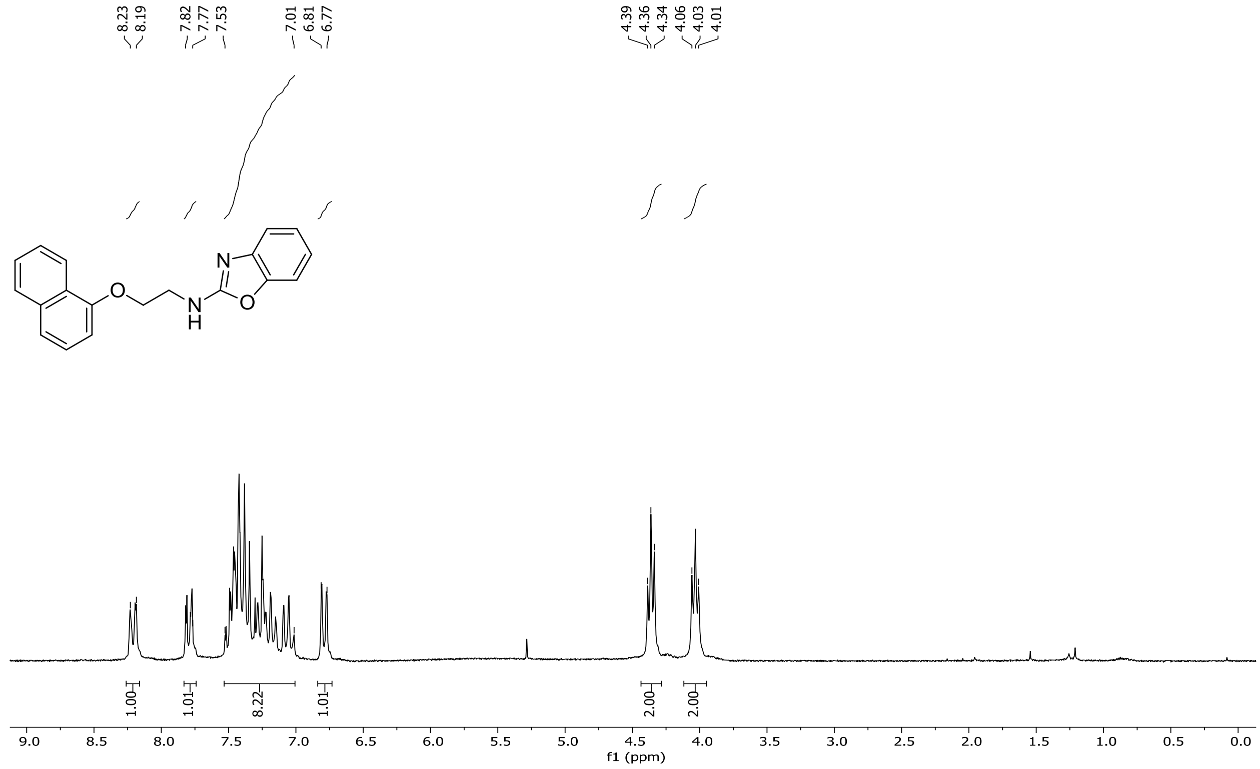

^{1}H NMR spectrum of **23d**.

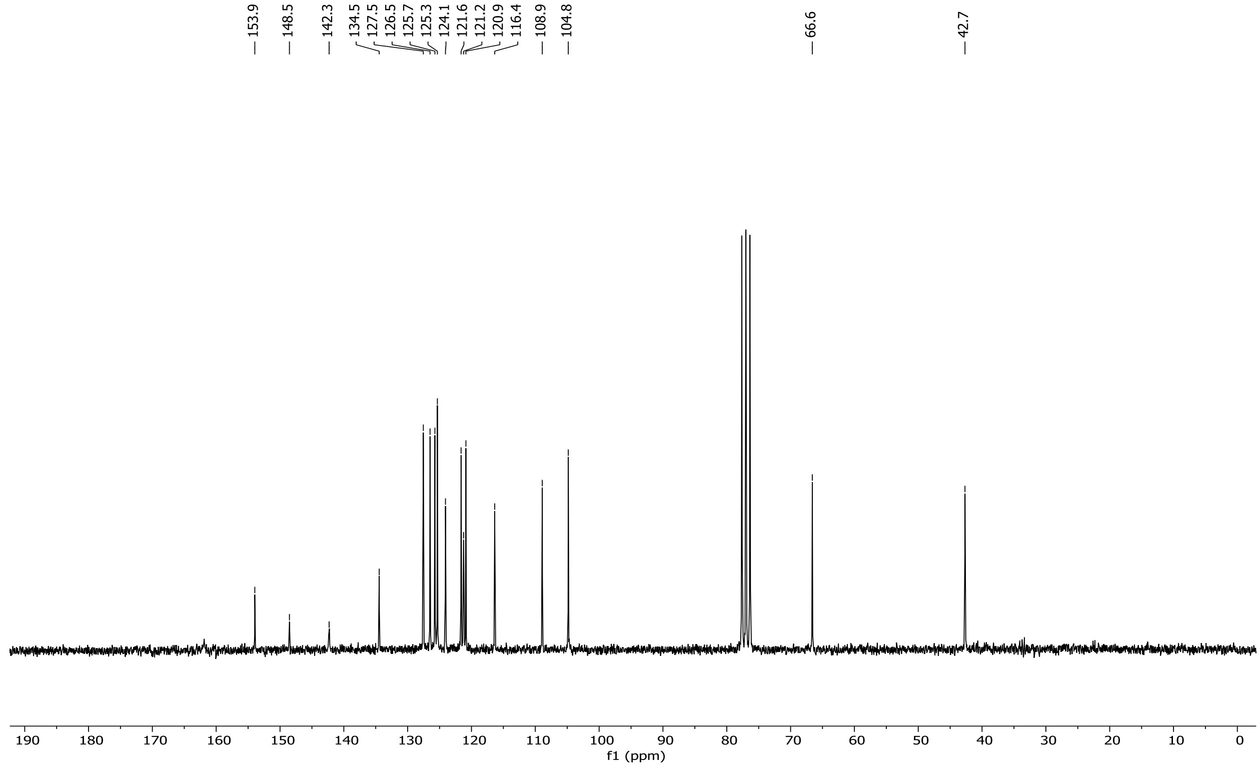

^{13}C NMR spectrum of **23d**.

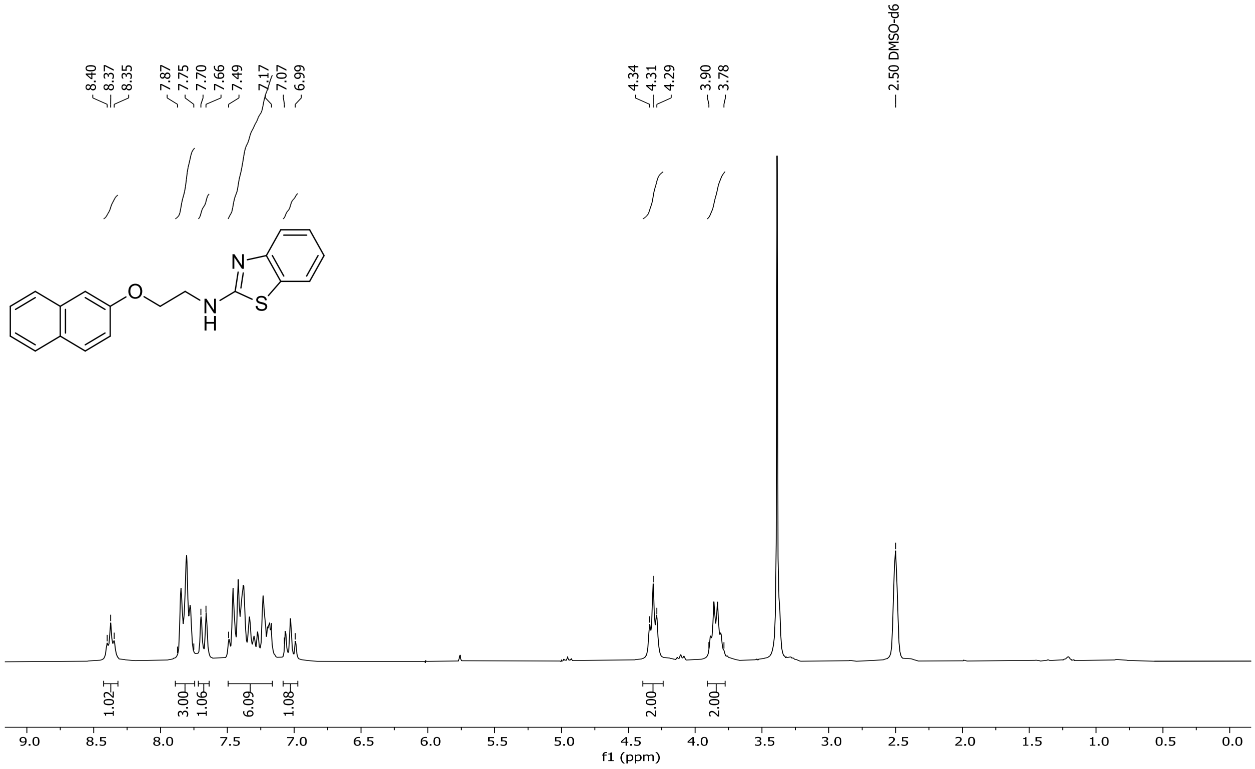

^{1}H NMR spectrum of **25a**.

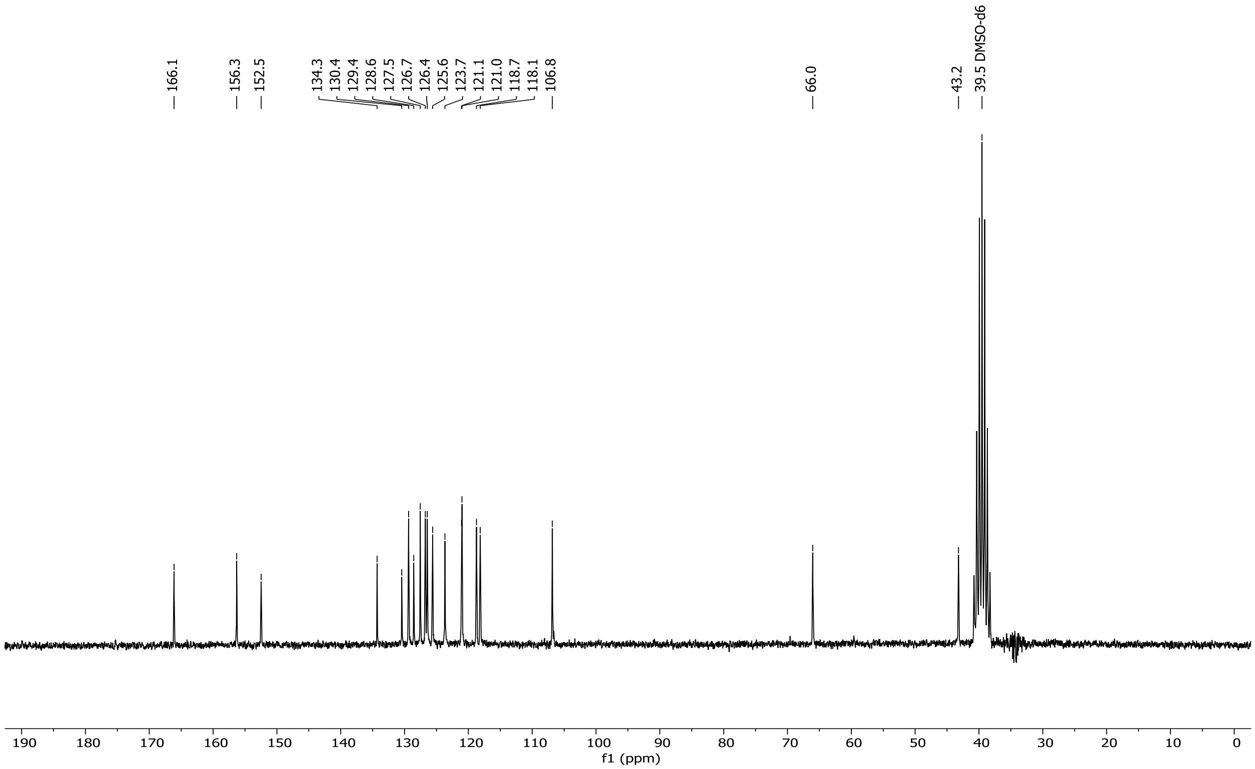

^{13}C NMR spectrum of **25a**.

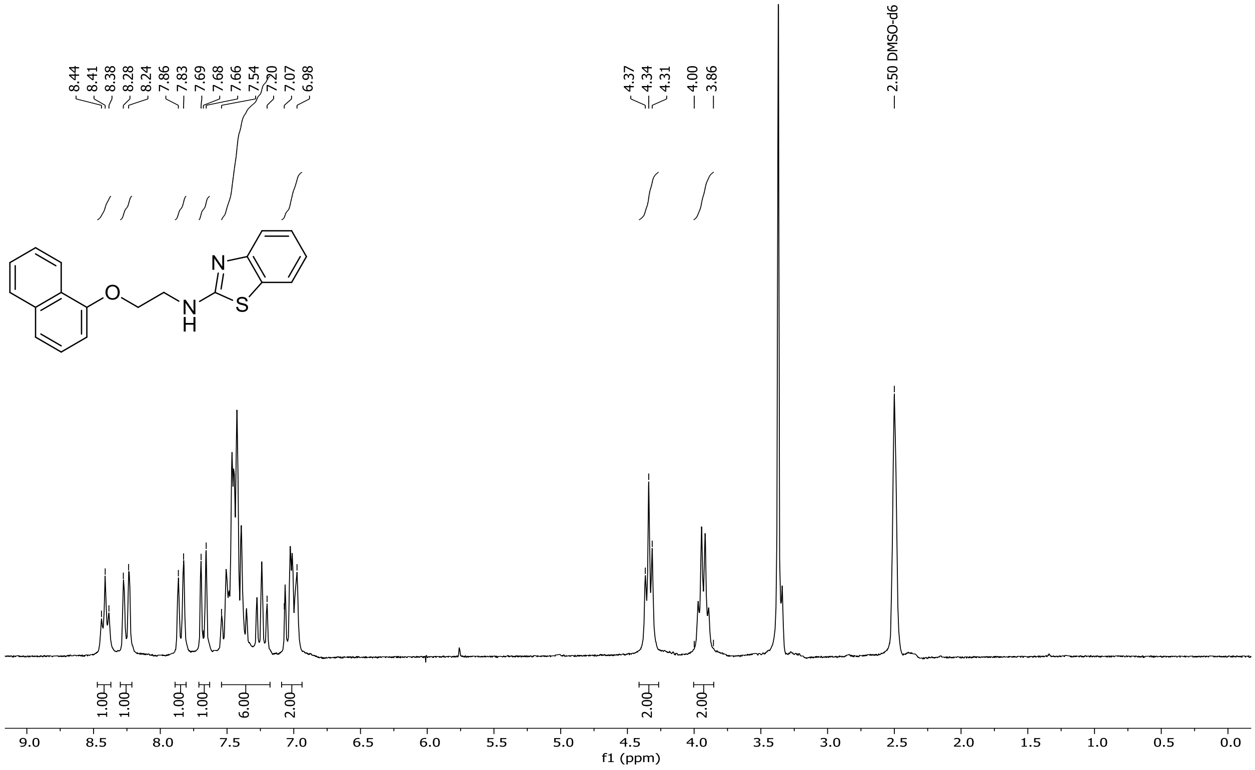

^{1}H NMR spectrum of **25b**.

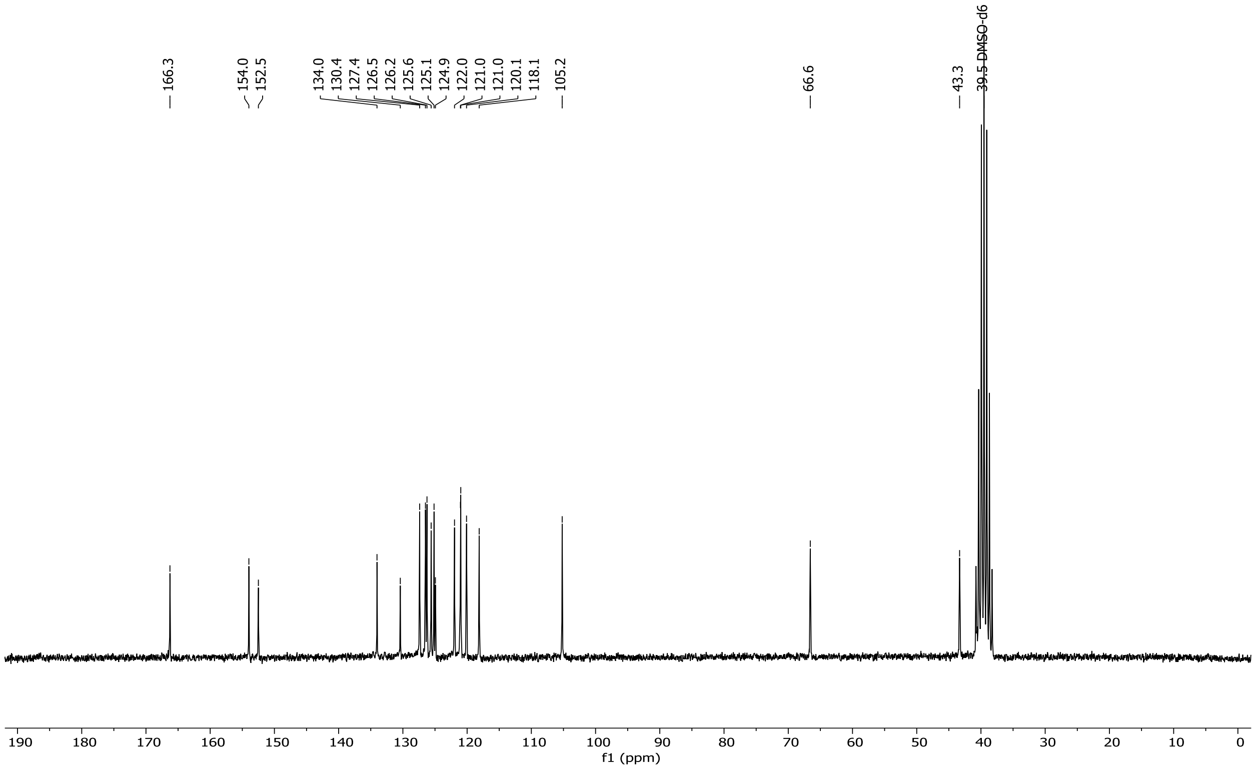

^{13}C NMR spectrum of **25b**.

HPLC traces

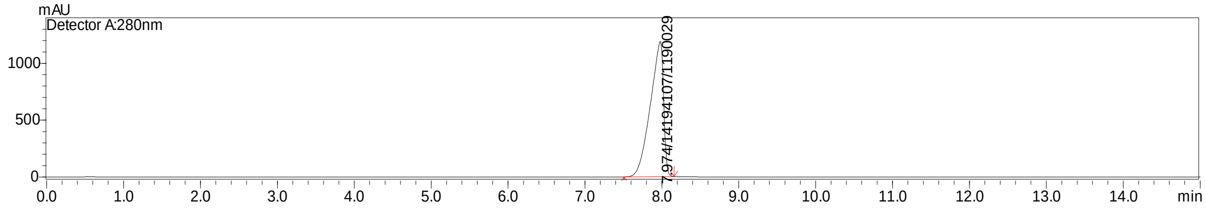

Peak#	Ret. Time	Conc.	Area	Height	Peak Start	Peak End	Area%	Resolution	Tailing F.
1	7.974	0.00000	14194107	1190029	7.508	8.158	100.0000	--	0.674

Figure S2: HPLC chromatogram of inhibitor **19d** (GK593) [500 ppm, H_2O/acetonitrile 65/35 v/v, gradient to 40:60 v/v for 15 min, 35 ^oC, 1 mL/min, Detector: 280 nm, Column: Thermo Scientific, Hypersil Silica (250 × 4.6 mm, 5 μm)].

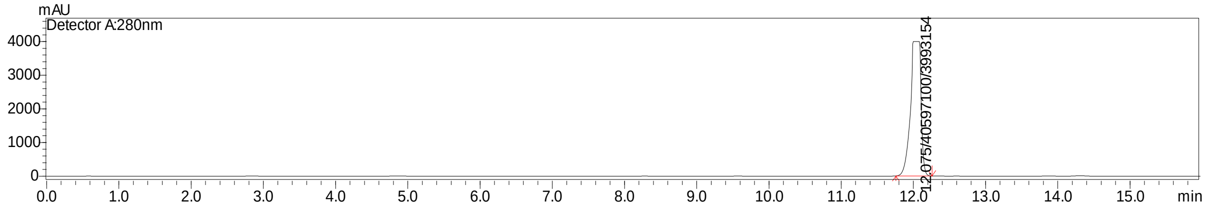

Peak#	Ret. Time	Conc.	Area	Height	Peak Start	Peak End	Area%	Resolution	Tailing F.
1	12.075	0.00000	40597100	3993154	11.758	12.258	100.0000	--	0.699

Figure S3: HPLC chromatogram of inhibitor **5b** (GK510) [500 ppm, H_2O/acetonitrile 70/30 v/v, gradient to 40:60 v/v for 15 min, 35 ^oC, 1 mL/min, Detector: 280 nm, Column: Thermo Scientific, Hypersil Silica (250 × 4.6 mm, 5 μm)].

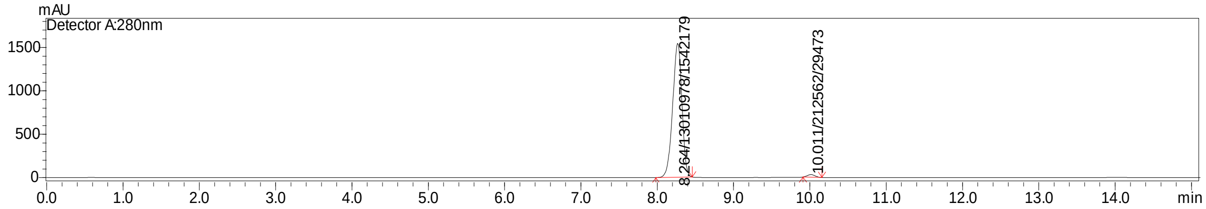

Peak#	Ret. Time	Conc.	Area	Height	Peak Start	Peak End	Area%	Resolution	Tailing F.
1	8.264	0.00000	13010978	1542179	7.983	8.458	98.3925	--	0.941
2	10.011	0.00000	212562	29473	9.908	10.158	1.6075	7.955	1.149

Figure S4: HPLC chromatogram of inhibitor **11** (GK521) [500 ppm, H_2O/acetonitrile 70/30 v/v, gradient to 40:60 v/v for 15 min, 35 ^oC, 1 mL/min, Detector: 280 nm, Column: Thermo Scientific, Hypersil Silica (250 × 4.6 mm, 5 μm)].

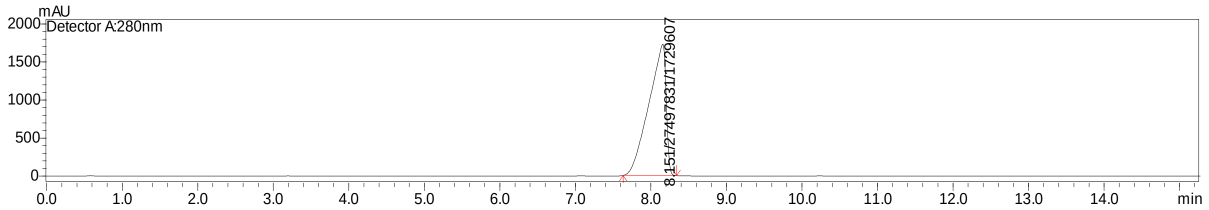

Peak#	Ret. Time	Conc.	Area	Height	Peak Start	Peak End	Area%	Resolution	Tailing F.
1	8.151	0.00000	27497831	1729607	7.633	8.342	100.0000	--	0.628

Figure S5: HPLC chromatogram of inhibitor **19a** (GK543) [500 ppm, H_2O/acetonitrile 65/35 v/v, gradient to 40:60 v/v for 15 min, 35 ^oC, 1 mL/min, Detector: 280 nm, Column: Thermo Scientific, Hypersil Silica (250 × 4.6 mm, 5 μm)].

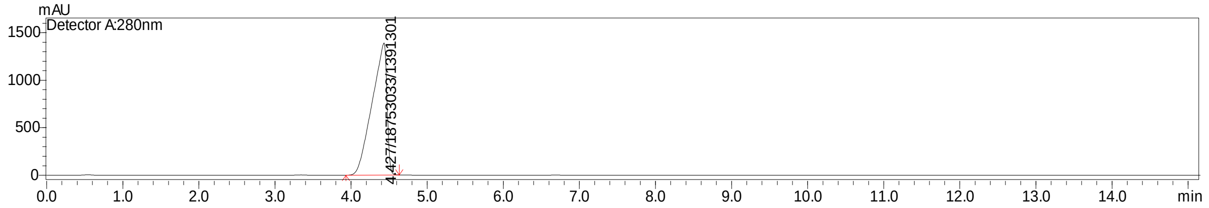

Peak#	Ret. Time	Conc.	Area	Height	Peak Start	Peak End	Area%	Resolution	Tailing F.
1	4.427	0.00000	18753033	1391301	3.933	4.633	100.0000	--	0.641

Figure S6: HPLC chromatogram of inhibitor **19c** (GK560) [500 ppm, H_2O/acetonitrile 65/35 v/v, gradient to 40:60 v/v for 15 min, 35 ^oC, 1 mL/min, Detector: 280 nm, Column: Thermo Scientific, Hypersil Silica (250 × 4.6 mm, 5 μm)].

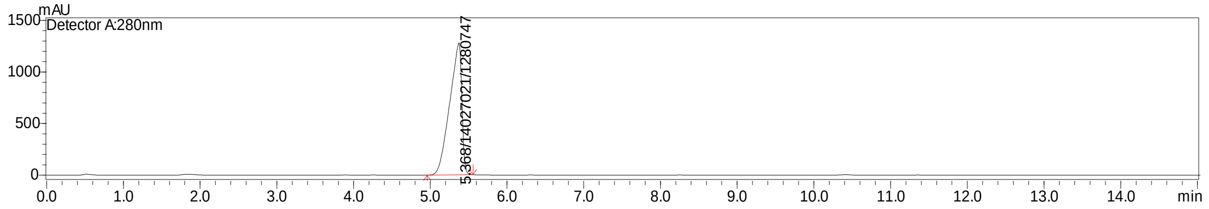

Peak#	Ret. Time	Conc.	Area	Height	Peak Start	Peak End	Area%	Resolution	Tailing F.
1	5.368	0.00000	14027021	1280747	4.958	5.558	100.0000	--	0.679

Figure S7: HPLC chromatogram of inhibitor **19b** (GK562) [500 ppm, H_2O/acetonitrile 70/30 v/v, gradient to 40:60 v/v for 15 min, 35 ^oC, 1 mL/min, Detector: 280 nm, Column: Thermo Scientific, Hypersil Silica (250 × 4.6 mm, 5 μm)].

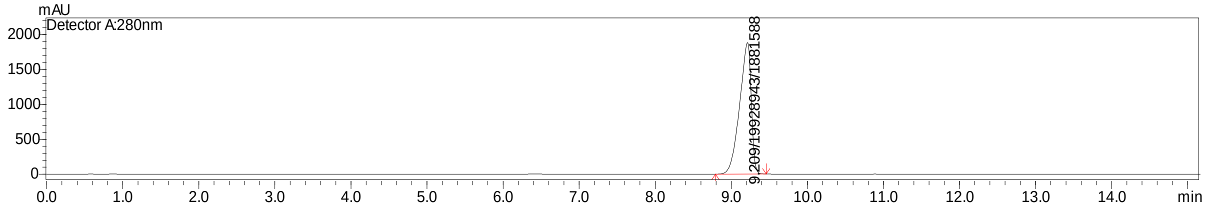

Peak#	Ret. Time	Conc.	Area	Height	Peak Start	Peak End	Area%	Resolution	Tailing F.
1	9.209	0.00000	19928943	1881588	8.792	9.458	100.0000	--	0.741

Figure S8: HPLC chromatogram of inhibitor **23a** (GK566) [500 ppm, H_2O/acetonitrile 65/35 v/v, gradient to 40:60 v/v for 15 min, 35 ^oC, 1 mL/min, Detector: 280 nm, Column: Thermo Scientific, Hypersil Silica (250 × 4.6 mm, 5 μm)].

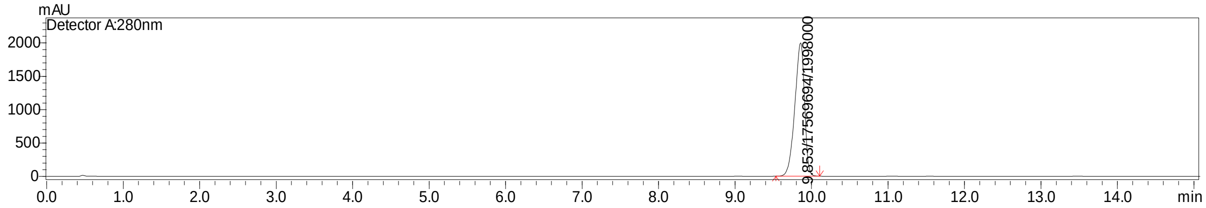

Peak#	Ret. Time	Conc.	Area	Height	Peak Start	Peak End	Area%	Resolution	Tailing F.
1	9.853	0.00000	17569694	1998000	9.533	10.108	100.0000	--	0.854

Figure S9: HPLC chromatogram of inhibitor **23c** (GK592) [500 ppm, H_2O/acetonitrile 70/30 v/v, gradient to 40:60 v/v for 15 min, 35 ^oC, 1 mL/min, Detector: 280 nm, Column: Thermo Scientific, Hypersil Silica (250 × 4.6 mm, 5 μm)].

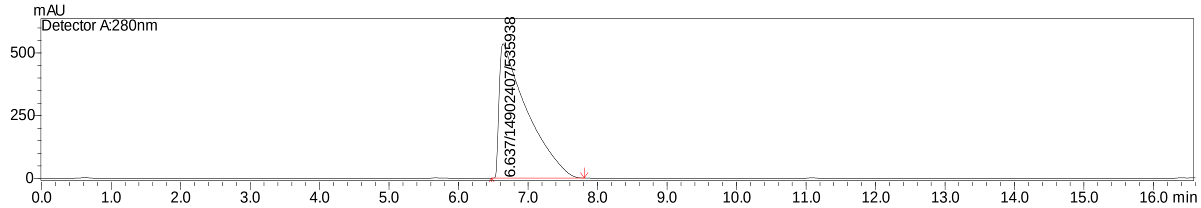

Peak#	Ret. Time	Conc.	Area	Height	Peak Start	Peak End	Area%	Resolution	Tailing F.
1	6.637	0.00000	14902407	535938	6.475	7.808	100.0000	--	5.189

Figure S10: HPLC chromatogram of inhibitor **19e** (GK610) [500 ppm, H_2O/acetonitrile 85/15 v/v, gradient to 55:45 v/v for 15 min, 35 ^oC, 1 mL/min, Detector: 280 nm, Column: Thermo Scientific, Hypersil Silica (250 × 4.6 mm, 5 μm)].
